# Supplementary material for: Multimodal profiling of pancreatic cancer reveals a TIMP-1-dominated secretory profile determining pro-tumor immunoinstruction in human cancers
Source: Cell Rep Med. 2026 Jan 20;7(1):102546. doi: 10.1016/j.xcrm.2025.102546 (PMC12866174; doi:10.1016/j.xcrm.2025.102546)
Supplement: Document S1. Figures S1–S7 [file mmc1.pdf]

Cell Reports Medicine, Volume *7*

Supplemental information

Multimodal profiling of pancreatic cancer reveals a TIMP-1-dominated secretory profile determining pro-tumor immunoinstruction in human cancers

Julian Frädrich, Carmen Mota Reyes, Michel Hendel, Vanessa Brunner, Batu Toledo, Damjan Manevski, Alexander Sommer, Daniel Häußler, Dominik Beck, Daniele Lucarelli, Jaime Martínez de Villareal, Lennard Halle, Raphael Kfuri-Rubens, Kaan Çifcibaşı, Anna Hirschberger, Rupert Öllinger, Percy A. Knolle, Katja Steiger, Roland Rad, Fabian J. Theis, Francisco X. Real, Stefanie Bärthel, Jan P. Böttcher, Dieter Saur, Ihsan Ekin Demir, and Achim Krüger

Fig. S1

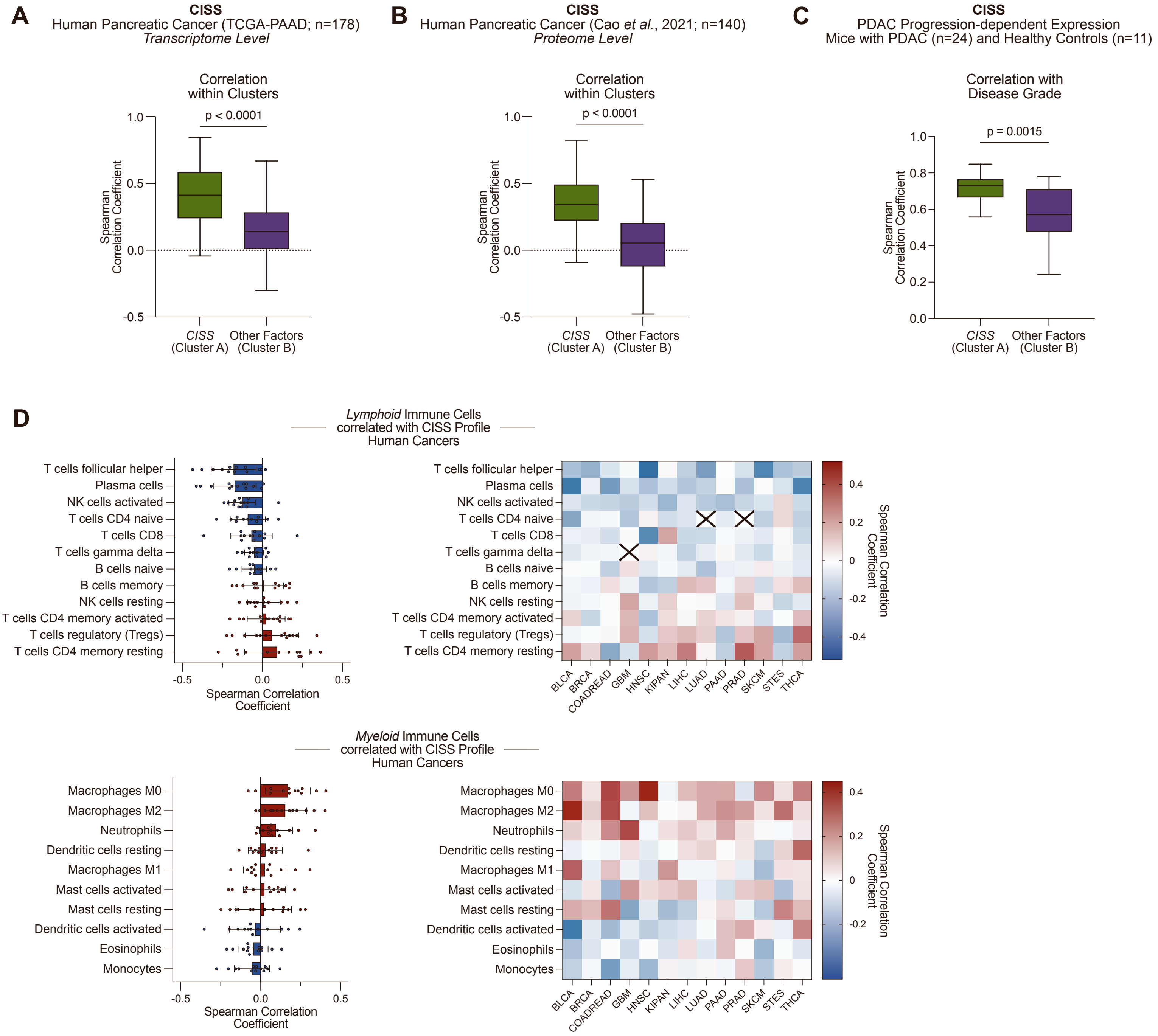

A
CISS
Human Pancreatic Cancer (TCGA-PAAD; n=178)
Transcriptome Level
Correlation within Clusters
p < 0.0001
Spearman Correlation Coefficient
CISS (Cluster A)
Other Factors (Cluster B)
B
CISS
Human Pancreatic Cancer (Cao et al., 2021; n=140)
Proteome Level
Correlation within Clusters
p < 0.0001
Spearman Correlation Coefficient
CISS (Cluster A)
Other Factors (Cluster B)
C
CISS
PDAC Progression-dependent Expression
Mice with PDAC (n=24) and Healthy Controls (n=11)
Correlation with Disease Grade
p = 0.0015
Spearman Correlation Coefficient
CISS (Cluster A)
Other Factors (Cluster B)
D
Lymphoid Immune Cells correlated with CISS Profile Human Cancers
T cells follicular helper
Plasma cells
NK cells activated
T cells CD4 naive
T cells CD8
T cells gamma delta
B cells naive
B cells memory
NK cells resting
T cells CD4 memory activated
T cells regulatory (Tregs)
T cells CD4 memory resting
Spearman Correlation Coefficient
BLCA
BRCA
COADREAD
GBM
HNSC
KIPAN
LIHC
LUAD
PAAD
PRAD
SKCM
STES
THCA
Myeloid Immune Cells correlated with CISS Profile Human Cancers
Macrophages M0
Macrophages M2
Neutrophils
Dendritic cells resting
Macrophages M1
Mast cells activated
Mast cells resting
Dendritic cells activated
Eosinophils
Monocytes
Spearman Correlation Coefficient

Fig. S1 Statistical evaluation of CISS in human PDAC and its correlation with PDAC progression in mice, related to Fig. 2. (A) Statistical comparison of Spearman correlation factors between CISS (Cluster A) and the other 17 factors (Cluster B) from TCGA-PAAD RNA-seq data (see *Fig. 2A*). Statistical comparison was performed using Mann-Whitney test. **(B)** Statistical comparison of Spearman correlation factors between CISS (Cluster A) and the other 17 factors (Cluster B) from PDAC tumor proteomics data (Cao cohort[S1]; see *Fig. 2B*). Statistical comparison was performed using Mann-Whitney test. **(C)** The 36 secreted factors (including the 19 CISS factors) in murine KPC tumors and control pancreata from Fig. 1 were correlated with disease grades (replaced by numerical values: 0 = control pancreas; 1 = early PDAC; 2 = grade 2; 3 = grade 3; 4 = grade 4) using Spearman correlation. For statistics, Spearman correlation coefficients were compared between CISS factors and the other 17 factors using unpaired Student's t test. Data represented as box and whiskers plots. **(D, Left)** Spearman correlation coefficient for each immune cell subtype in all entities (dots), separated by lymphoid and myeloid cells. Data showing all individual points. **(D, Right)** Heatmap visualization of left panels across human cancer entities. X indicates cell type was not detected. Also see *Fig. 2I.*

Fig. S2

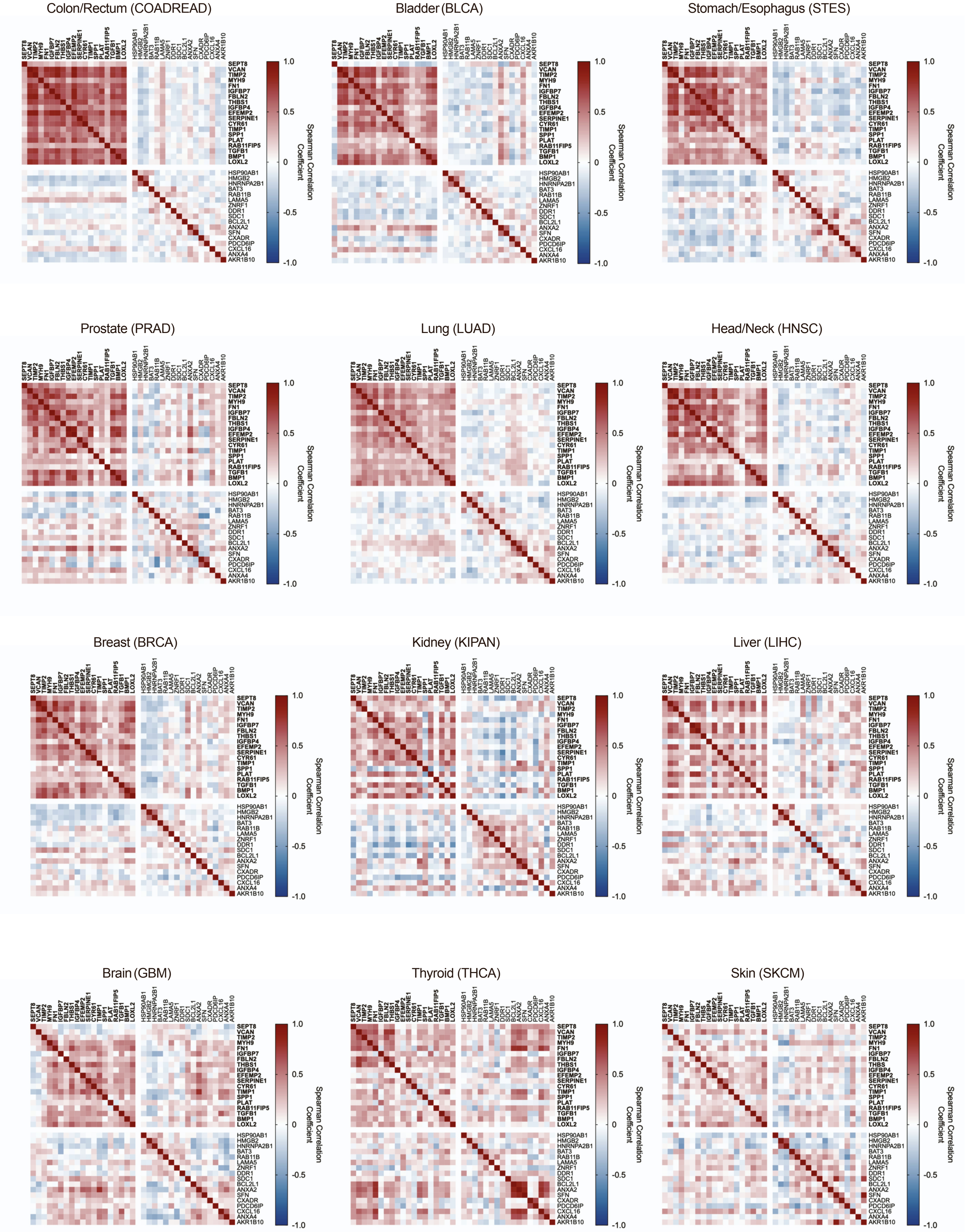
Colon/Rectum (COADREAD)
Bladder (BLCA)
Stomach/Esophagus (STES)
Prostate (PRAD)
Lung (LUAD)
Head/Neck (HNSC)
Breast (BRCA)
Kidney (KIPAN)
Liver (LIHC)
Brain (GBM)
Thyroid (THCA)
Skin (SKCM)
SEPT8
VCAN
TIMP2
MYH9
FN1
IGFBP7
FBLN2
THBS1
IGFBP4
EFEMP2
SERPINE1
CYR61
TIMP1
SPP1
PLAT
RAB11FIP5
TGFB1
BMP1
LOXL2
HSP90AB1
HMGB2
HNRNPA2B1
BAT3
RAB11B
LAMA5
ZNRF1
DDR1
SDC1
BCL2L1
ANXA2
SFN
CXADR
PDCD6IP
CXCL16
ANXA4
AKR1B10
1.0
0.5
0
-0.5
-1.0
Spearman Correlation Coefficient

Fig. S2 Pan-Cancer validation of CISS across human solid cancers, related to Fig. 2. Correlation analysis of mRNA expression levels of the 36 secreted factors (see *Fig. 2A*) in bulk tumor RNA-seq data from TCGA cohorts (see *Fig. 2G,H*). Order of genes (including the CISS factors in the upper left cluster) are aligned with the TCGA-PAAD reference cohort (see *Fig. 2A*). BLCA, bladder urothelial carcinoma (n = 408); BRCA, breast invasive carcinoma (n = 1,093); COADREAD, colorectal adenocarcinoma (n = 379); GBM, glioblastoma multiforme (n = 152); HNSC, head and neck squamous cell carcinoma (n = 520); KIPAN, Pan-kidney cancer cohort (n = 889); LIHC, liver hepatocellular carcinoma (n = 371); LUAD, lung adenocarcinoma (n = 515); PAAD, pancreatic adenocarcinoma (n = 178); PRAD, prostate adenocarcinoma (n = 497); SKCM, skin cutaneous melanoma (n = 103); STES, stomach and esophageal carcinoma (n = 599); THCA, thyroid carcinoma (n = 501).

Fig. S3

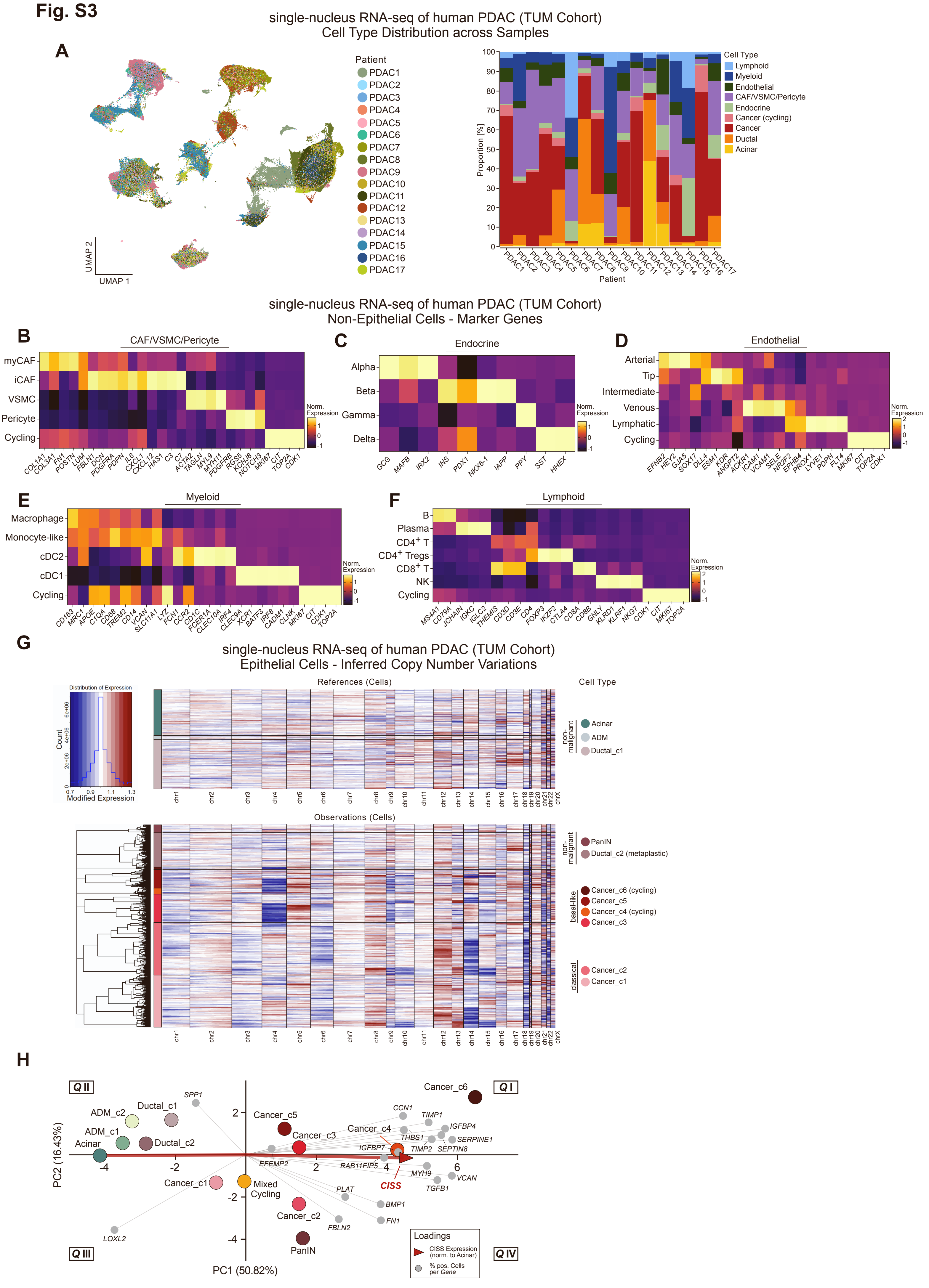
single-nucleus RNA-seq of human PDAC (TUM Cohort)
Cell Type Distribution across Samples
A
UMAP 1
UMAP 2
Patient
PDAC1
PDAC2
PDAC3
PDAC4
PDAC5
PDAC6
PDAC7
PDAC8
PDAC9
PDAC10
PDAC11
PDAC12
PDAC13
PDAC14
PDAC15
PDAC16
PDAC17
Proportion [%]
Patient
Cell Type
Lymphoid
Myeloid
Endothelial
CAF/VSMC/Pericyte
Endocrine
Cancer (cycling)
Cancer
Ductal
Acinar
single-nucleus RNA-seq of human PDAC (TUM Cohort)
Non-Epithelial Cells - Marker Genes
B
CAF/VSMC/Pericyte
myCAF
iCAF
VSMC
Pericyte
Cycling
COL1A1 COL3A1 FN1 POSTN LUM FBLN1 DCN PDGFRA PDPN IL6 CXCL1 CXCL12 HAS1 C3 C7 ACTA2 TAGLN MYL9 MYH11 PDGFRB RGS5 KCNJ8 NOTCH3 MKI67 CIT TOP2A CDK1
Norm. Expression
C
Endocrine
Alpha
Beta
Gamma
Delta
GCG MAFB IRX2 INS PDX1 NKX6-1 IAPP PPY SST HHEX
D
Endothelial
Arterial
Tip
Intermediate
Venous
Lymphatic
Cycling
EFNB2 HEY2 GJA5 SOX17 DLL4 ESM1 KDR ANGPT2 ACKR1 ICAM1 VCAM1 SELE NR2F2 EPHB4 PROX1 LYVE1 PDPN FLT4 MKI67 CIT TOP2A CDK1
E
Myeloid
Macrophage
Monocyte-like
cDC2
cDC1
Cycling
CD163 MRC1 APOE C1QA CD68 TREM2 CD14 VCAN SLC11A1 LYZ FCN1 CCR2 CD1C FCER1A CLEC10A IRF4 CLEC9A XCR1 BATF3 IRF8 CADM1 CLNK MKI67 CIT CDK1 TOP2A
F
Lymphoid
B
Plasma
CD4+ T
CD4+ Tregs
CD8+ T
NK
Cycling
MS4A1 CD79A JCHAIN IGKC IGLC2 THEMIS CD3D CD3E CD4 FOXP3 IKZF2 CTLA4 CD8A CD8B GNLY KLRD1 KLRF1 NKG7 CDK1 CIT MKI67 TOP2A
G
single-nucleus RNA-seq of human PDAC (TUM Cohort)
Epithelial Cells - Inferred Copy Number Variations
Distribution of Expression
Count
Modified Expression
References (Cells)
Cell Type
non-malignant
Acinar
ADM
Ductal_c1
Observations (Cells)
PanIN
Ductal_c2 (metaplastic)
basal-like
Cancer_c6 (cycling)
Cancer_c5
Cancer_c4 (cycling)
Cancer_c3
classical
Cancer_c2
Cancer_c1
chr1 chr2 chr3 chr4 chr5 chr6 chr7 chr8 chr9 chr10 chr11 chr12 chr13 chr14 chr15 chr16 chr17 chr18 chr19 chr20 chr21 chr22 chrX
H
Q II
Q I
Q III
Q IV
Cancer_c6
SPP1
ADM_c2
Ductal_c1
Cancer_c5
CCN1
TIMP1
IGFBP4
ADM_c1
Acinar
Ductal_c2
Cancer_c3
Cancer_c4
THBS1
SERPINE1
IGFBP7
TIMP2
SEPTIN8
EFEMP2
RAB11FIP5
MYH9
VCAN
Cancer_c1
Mixed Cycling
PLAT
CISS
TGFB1
BMP1
Cancer_c2
FBLN2
FN1
LOXL2
PanIN
Loadings
CISS Expression (norm. to Acinar)
% pos. Cells per Gene
PC1 (50.82%)
PC2 (16.43%)

Fig. S3 Cell types, inferred CNVs, and CISS expression across PDAC patient tumors, related to Fig. 3. (A) (Left) UMAP embedding of single-nucleus profiles (dots) derived from snRNA-seq of treatment-naïve PDAC patient tumors (n = 17) colored by patients (color legend, right). Post-hoc cell-type annotations are shown in *Fig. 3A*. (Right) Cell type distributions shown by proportions (y axis) of each cell type (color legend, right) across patient tumor samples (columns, x axis). **(B-F)** snRNA-seq captured heterogenous stromal (B), endocrine (C), endothelial (D), myeloid immune (E), and lymphoid immune (F) cell subsets in PDAC patient tumors (see *Fig. 3C*). Heatmap visualization of selected marker gene expression (z-scores) across identified cell subsets. **(G)** Inferred copy number variations (CNVs) by inferCNV analysis of epithelial subsets as annotated in *Fig. 3D,E*. Inferred amplifications (red) and deletions (blue) based on gene expression (color bar) are shown in each chromosomal locus (columns) from each cell (rows), clustered by annotated epithelial cell types (color legend, right). Putatively malignant, Ductal_c2, PanIN-like cells were used as observational groups (lower panel) and compared to Acinar, ADM, Ductal_c1 cells as reference cells (upper panel). **(H)** Principal component analysis (PCA) of intratumoral epithelial subsets (see *Fig. 3D-H*), based on total CISS (red triangle) and individual CISS factors (grey dots). PC scores of each cell subtype (colored dots) are indicated.

Fig. S4

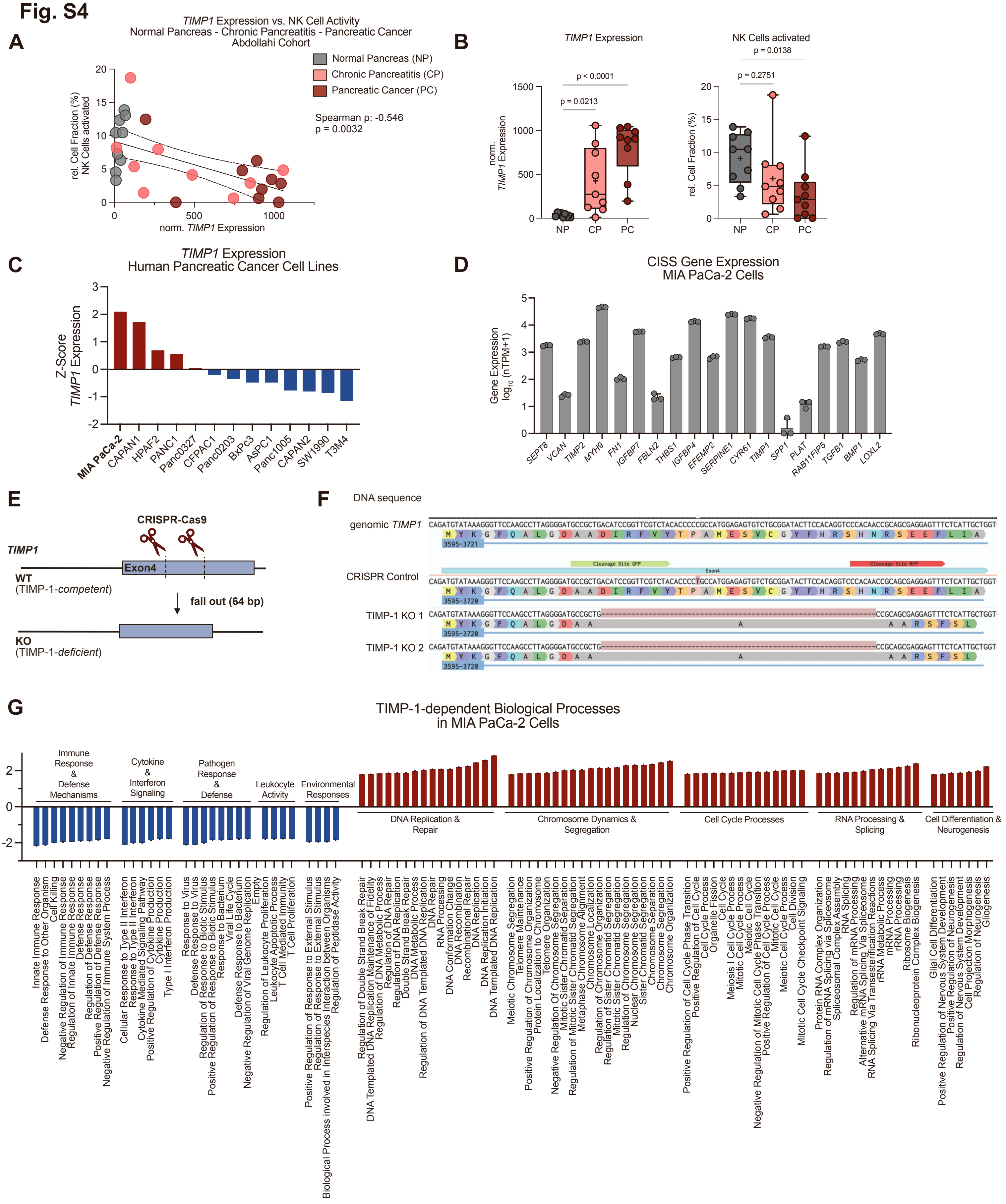
A
TIMP1 Expression vs. NK Cell Activity
Normal Pancreas - Chronic Pancreatitis - Pancreatic Cancer
Abdollahi Cohort
Normal Pancreas (NP)
Chronic Pancreatitis (CP)
Pancreatic Cancer (PC)
Spearman ρ: -0.546
p = 0.0032
rel. Cell Fraction (%)
NK Cells activated
norm. TIMP1 Expression
B
TIMP1 Expression
p < 0.0001
p = 0.0213
norm. TIMP1 Expression
NK Cells activated
p = 0.0138
p = 0.2751
rel. Cell Fraction (%)
NP
CP
PC
C
TIMP1 Expression
Human Pancreatic Cancer Cell Lines
Z-Score
TIMP1 Expression
MIA PaCa-2
CAPAN1
HPAF2
PANC1
Panc0327
CFPAC1
Panc0203
BxPc3
AsPC1
Panc1005
CAPAN2
SW1990
T3M4
D
CISS Gene Expression
MIA PaCa-2 Cells
Gene Expression
log10 (nTPM+1)
SEPT8
VCAN
TIMP2
MYH9
FN1
IGFBP7
FBLN2
THBS1
IGFBP4
EFEMP2
SERPINE1
CYR61
TIMP1
SPP1
PLAT
RAB11FIP5
TGFB1
BMP1
LOXL2
E
CRISPR-Cas9
TIMP1
Exon4
WT
(TIMP-1-competent)
fall out (64 bp)
KO
(TIMP-1-deficient)
F
DNA sequence
genomic TIMP1
CRISPR Control
TIMP-1 KO 1
TIMP-1 KO 2
Cleavage Site GFP
Cleavage Site RFP
Exon4
3595-3721
3595-3720
G
TIMP-1-dependent Biological Processes
in MIA PaCa-2 Cells
NES
Immune Response & Defense Mechanisms
Cytokine & Interferon Signaling
Pathogen Response & Defense
Leukocyte Activity
Environmental Responses
DNA Replication & Repair
Chromosome Dynamics & Segregation
Cell Cycle Processes
RNA Processing & Splicing
Cell Differentiation & Neurogenesis
Innate Immune Response
Defense Response to Other Organism
Cell Killing
Negative Regulation of Immune Response
Regulation of Innate Immune Response
Defense Response
Regulation of Defense Response
Positive Regulation of Defense Response
Negative Regulation of Immune System Process
Cellular Response to Type II Interferon
Response to Type II Interferon
Cytokine Mediated Signaling Pathway
Positive Regulation of Cytokine Production
Cytokine Production
Type I Interferon Production
Response to Virus
Defense Response to Virus
Regulation of Response to Biotic Stimulus
Positive Regulation of Response to Biotic Stimulus
Response to Bacterium
Viral Life Cycle
Defense Response to Bacterium
Negative Regulation of Viral Genome Replication
Empty
Regulation of Leukocyte Proliferation
Leukocyte Apoptotic Process
T Cell Mediated Immunity
T Cell Proliferation
Positive Regulation of Response to External Stimulus
Regulation of Response to External Stimulus
Biological Process involved in Interspecies Interaction between Organisms
Regulation of Peptidase Activity
Regulation of Double Strand Break Repair
DNA Templated DNA Replication Maintenance of Fidelity
Regulation of DNA Metabolic Process
Regulation of DNA Repair
Regulation of Double Strand Break Repair
Double Strand Break Repair
DNA Metabolic Process
Regulation of DNA Templated DNA Replication
DNA Repair
RNA Processing
DNA Conformation Change
DNA Recombination
Recombinational Repair
DNA Replication
DNA Replication Initiation
DNA Templated DNA Replication
Meiotic Chromosome Segregation
Telomere Maintenance
Regulation of Chromosome Organization
Positive Regulation of Chromosome Organization
Protein Localization to Chromosome
Telomere Organization
Negative Regulation Of Chromosome Segregation
Mitotic Sister Chromatid Separation
Regulation of Mitotic Sister Chromatid Segregation
Metaphase Chromosome Alignment
Chromosome Localization
Regulation of Chromosome Organization
Regulation of Sister Chromatid Segregation
Mitotic Sister Chromatid Segregation
Regulation of Chromosome Segregation
Nuclear Chromosome Segregation
Sister Chromatid Segregation
Chromosome Segregation
Chromosome Organization
Positive Regulation of Cell Cycle Phase Transition
Positive Regulation of Cell Cycle
Cell Cycle Process
Organelle Fission
Cell Cycle
Meiosis I Cell Cycle Process
Mitotic Cell Cycle Process
Meiotic Cell Cycle
Negative Regulation of Mitotic Cell Cycle Phase Transition
Positive Regulation of Cell Cycle Process
Mitotic Cell Cycle
Meiotic Cell Cycle Process
Cell Division
Mitotic Cell Cycle Checkpoint Signaling
Protein RNA Complex Organization
Regulation of mRNA Splicing via Spliceosome
Spliceosomal Complex Assembly
RNA Splicing
Regulation of mRNA Processing
Alternative mRNA Splicing Via Spliceosome
RNA Splicing Via Transesterification Reactions
rRNA Metabolic Process
mRNA Processing
rRNA Processing
Ribosome Biogenesis
Ribonucleoprotein Complex Biogenesis
Glial Cell Differentiation
Positive Regulation of Nervous System Development
Regulation of Nervous System Development
Cell Projection Morphogenesis
Regulation of Neurogenesis
Gliogenesis

Fig. S4 Functional role of TIMP-1 in TIMP-1^{hi} basal-like MIA PaCa-2 PC cell line, related to Fig. 4. **(A)** Validation of the correlation between *TIMP1* expression and reduced NK cell activity (see *Fig. 4D-F*) in an external patient cohort (Abdollahi *et al.*[S2]) comprising normal pancreas controls (n = 9), chronic pancreatitis (n = 9), and PC patients (n = 9). **(B)** *TIMP1* expression (left) and CIBERSORTx-determined NK cell activity (right) from (A). Significance was calculated by Kruskal-Wallis test and Dunn's test for multiple comparisons correction. **(C)** Screening of *TIMP1* expression (z-scores) data from two previously published RNA-seq datasets of PC cell lines (Moffitt *et al.*[S3], Diaferia *et al.*[S4]) revealed mesenchymal/basal-like MIA PaCa-2 cell line as model system for TIMP-1^{hi} basal-like cancer cells. **(D)** RNA-seq (see *Fig. 4J*) showed expression of all CISS factors in MIA PaCa-2 cell line. **(E)** Graphical scheme of *TIMP1* sites targeted by single-guide RNAs to generate MIA PaCa-2 TIMP-1 KO cells. bp; base pairs. **(F)** Sanger sequencing for validation of CRISPR-Cas9-induced frame-shift mutation in TIMP-1 KO 1 and KO 2 cells and absence of altered amino acid sequence in CRISPR Control cells (silent point mutation g.64C>T). **(G)** TIMP-1-dependent biological processes in MIA PaCa-2 cells revealed by GSEA (see *Fig. 4M*). NES, normalized enrichment score. Data in (A,B,D) showing all individual points and represented as z-score bars (C), mean ± s.d. (D), and NES bars (G).

Fig. S5

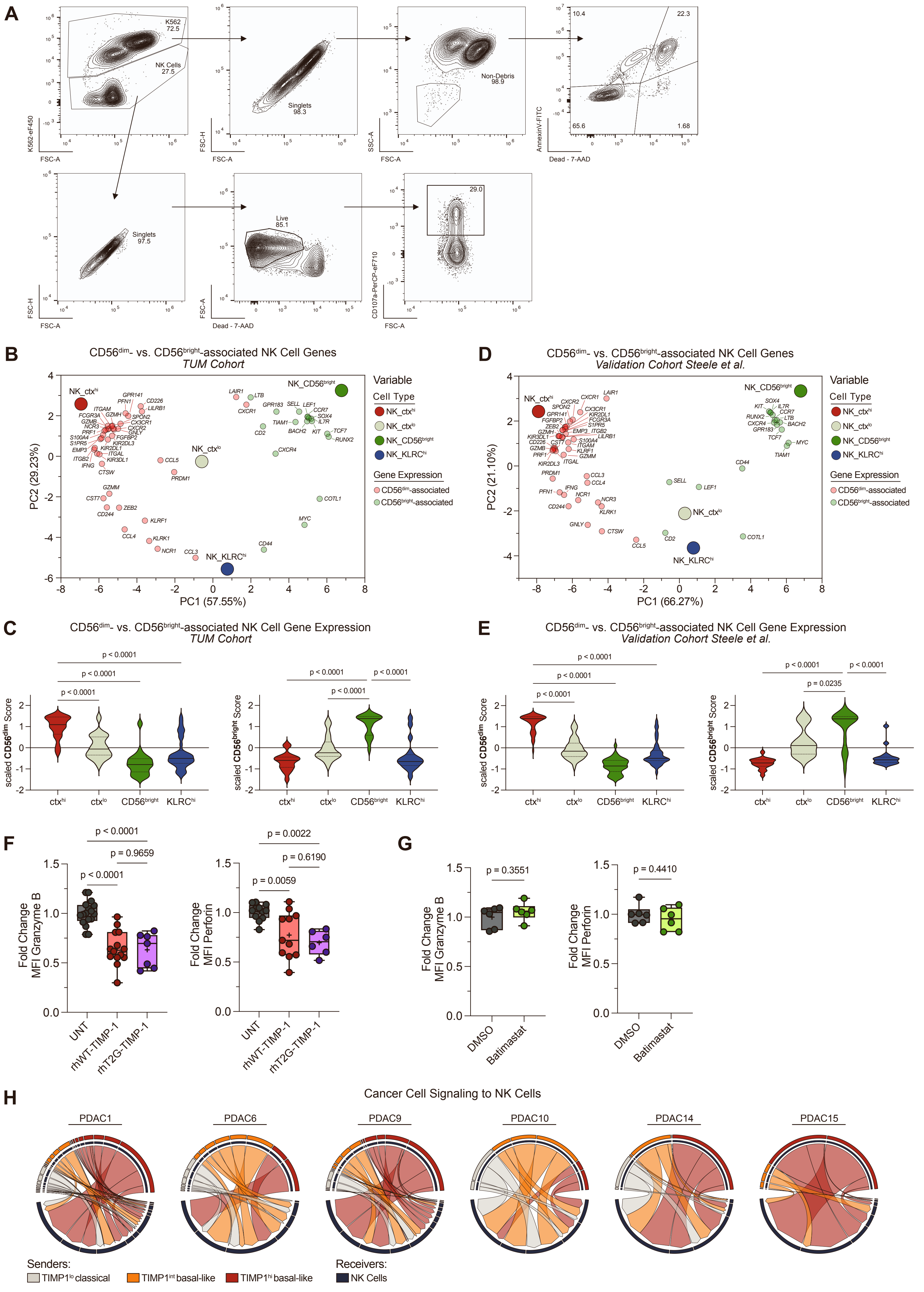

A
K562 72.5
NK Cells 27.5
Singlets 98.3
Non-Debris 98.9
10.4
22.3
65.6
1.68
Singlets 97.5
Live 85.1
29.0
K562-eF450
FSC-A
FSC-H
SSC-A
AnnexinV-FITC
Dead - 7-AAD
CD107a-PerCP-eF710
B
CD56dim- vs. CD56bright-associated NK Cell Genes
TUM Cohort
PC1 (57.55%)
PC2 (29.23%)
D
CD56dim- vs. CD56bright-associated NK Cell Genes
Validation Cohort Steele et al.
PC1 (66.27%)
PC2 (21.10%)
Variable
Cell Type
NK_ctxhi
NK_ctxlo
NK_CD56bright
NK_KLRChi
Gene Expression
CD56dim-associated
CD56bright-associated
C
CD56dim- vs. CD56bright-associated NK Cell Gene Expression
TUM Cohort
E
CD56dim- vs. CD56bright-associated NK Cell Gene Expression
Validation Cohort Steele et al.
scaled CD56dim Score
scaled CD56bright Score
ctxhi
ctxlo
CD56bright
KLRChi
p < 0.0001
p = 0.0235
F
Fold Change MFI Granzyme B
Fold Change MFI Perforin
UNT
rhWT-TIMP-1
rhT2G-TIMP-1
p = 0.9659
p = 0.0022
p = 0.6190
p = 0.0059
G
DMSO
Batimastat
p = 0.3551
p = 0.4410
H
Cancer Cell Signaling to NK Cells
PDAC1
PDAC6
PDAC9
PDAC10
PDAC14
PDAC15
Senders:
TIMP1lo classical
TIMP1int basal-like
TIMP1hi basal-like
Receivers:
NK Cells

Fig. S5 TIMP-1-dependent regulation of NK cell cytotoxic capacity, related to Fig. 4,5,6. (A) Representative flow cytometry plots showing the gating strategy for NK cell cytotoxicity assays (see *Fig. 4*, *Fig. 6*). **(B-E)** Classification of intratumoral NK cell clusters based on $CD56^{dim}$ and $CD56^{bright}$ NK cell gene expression signatures in our cohort (B,C) or Steele cohort[S5] (D,E) (see Fig. *5A-F*). $CD56^{dim}$ and $CD56^{bright}$ NK cell marker genes were curated from previously published literature[S6,S7,S8,S9], and their expression was associated with intratumoral NK cell clusters (see Fig. 5B,D) using PCA for individual genes (B,D) or as grouped expression scores (C,E). Significance (C,E) was calculated using one-way ANOVA for matched data (genes) and Dunnett test for multiple comparisons correction between indicated groups. **(F)** NK cell granzyme B and perforin expression upon exposure to 500 ng/mL recombinant human (rh) WT-TIMP-1 vs. 500 ng/mL T2G-TIMP-1 (granzyme B: UNT, n = 17; WT-TIMP-1, n = 14; T2G-TIMP-1, n = 7; perforin: UNT, n = 12; WT-TIMP-1, n = 11; T2G-TIMP-1, n = 6). UNT and WT-TIMP-1 shared with *Fig. 5G*. **(G)** NK cell granzyme B and perforin expression upon exposure to batimastat or DMSO control (granzyme B and perforin: DMSO, n = 6; Batimastat, n = 6). Significance was calculated by (F) one-way ANOVA and Tukey test for multiple comparisons correction or (G) unpaired Student's t test. **(H)** Inferred cancer cell-to-NK cell signaling using CellChat individually for each patient (also see *Fig. 5N*). Data represented as violin plots (C,E) or showing biological replicates from 3 independent experiments and represented as box and whiskers plots (F,G).

Fig. S6

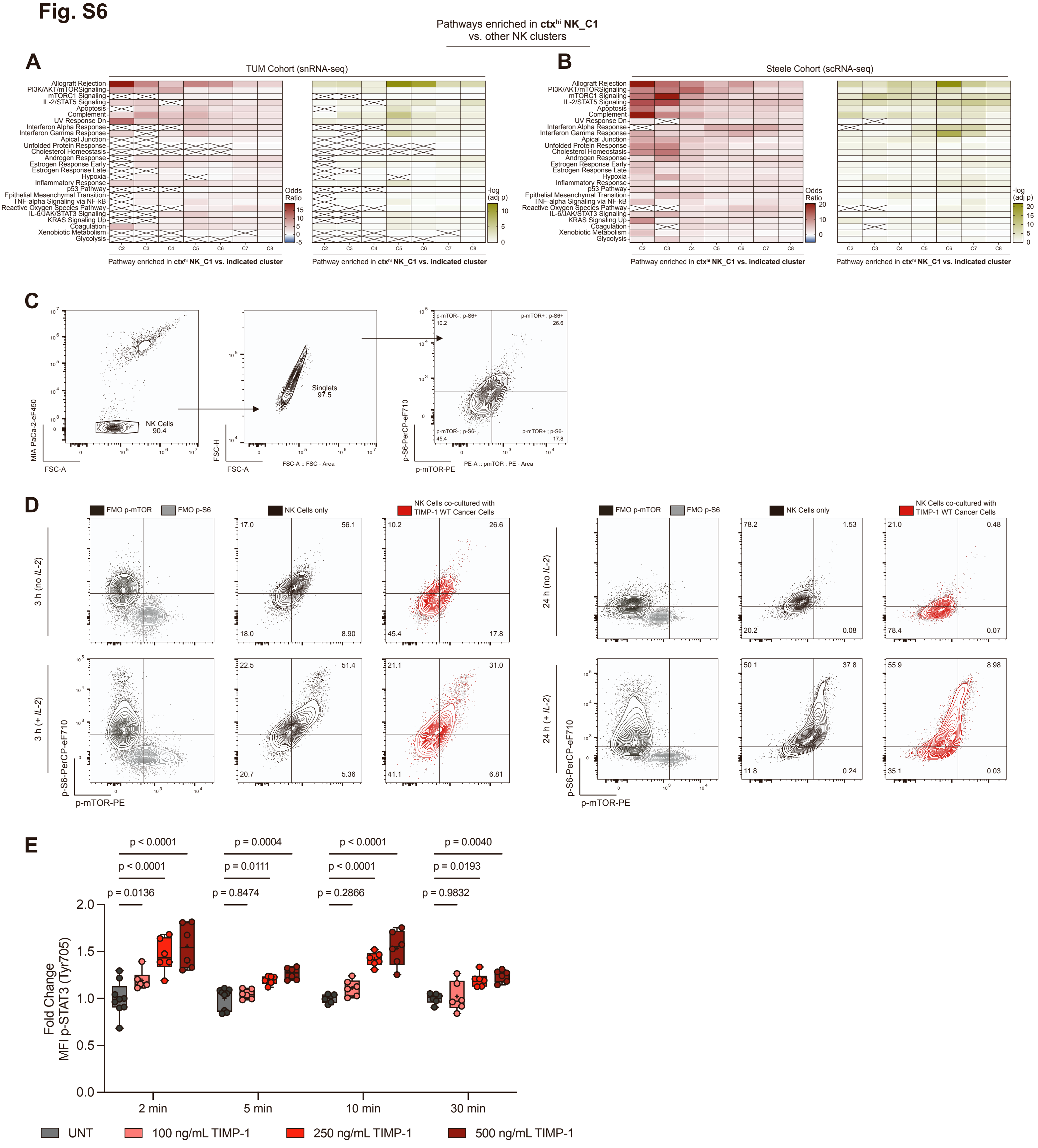

Pathways enriched in ctxhi NK_C1
vs. other NK clusters
A
TUM Cohort (snRNA-seq)
B
Steele Cohort (scRNA-seq)
Allograft Rejection
PI3K/AKT/mTORSignaling
mTORC1 Signaling
IL-2/STAT5 Signaling
Apoptosis
Complement
UV Response Dn
Interferon Alpha Response
Interferon Gamma Response
Apical Junction
Unfolded Protein Response
Cholesterol Homeostasis
Androgen Response
Estrogen Response Early
Estrogen Response Late
Hypoxia
Inflammatory Response
p53 Pathway
Epithelial Mesenchymal Transition
TNF-alpha Signaling via NF-kB
Reactive Oxygen Species Pathway
IL-6/JAK/STAT3 Signaling
KRAS Signaling Up
Coagulation
Xenobiotic Metabolism
Glycolysis
C2 C3 C4 C5 C6 C7 C8
Odds Ratio
-log (adj p)
Pathway enriched in ctxhi NK_C1 vs. indicated cluster
C
MIA PaCa-2-eF450
FSC-A
NK Cells 90.4
FSC-H
Singlets 97.5
FSC-A :: FSC - Area
p-S6-PerCP-eF710
p-mTOR-PE
PE-A :: pmTOR : PE - Area
p-mTOR- ; p-S6+ 10.2
p-mTOR+ ; p-S6+ 26.6
p-mTOR- ; p-S6- 45.4
p-mTOR+ ; p-S6- 17.8
D
FMO p-mTOR
FMO p-S6
NK Cells only
NK Cells co-cultured with TIMP-1 WT Cancer Cells
3 h (no IL-2)
3 h (+ IL-2)
24 h (no IL-2)
24 h (+ IL-2)
17.0 56.1 18.0 8.90
10.2 26.6 45.4 17.8
22.5 51.4 20.7 5.36
21.1 31.0 41.1 6.81
78.2 1.53 20.2 0.08
21.0 0.48 78.4 0.07
50.1 37.8 11.8 0.24
55.9 8.98 35.1 0.03
p-S6-PerCP-eF710
p-mTOR-PE
E
p < 0.0001
p < 0.0001
p = 0.0136
p = 0.0004
p = 0.0111
p = 0.8474
p < 0.0001
p < 0.0001
p = 0.2866
p = 0.0040
p = 0.0193
p = 0.9832
Fold Change
MFI p-STAT3 (Tyr705)
2 min
5 min
10 min
30 min
UNT
100 ng/mL TIMP-1
250 ng/mL TIMP-1
500 ng/mL TIMP-1

Fig. S6 TIMP-1-dependent regulation of signaling pathways in primary NK cells, related to Fig. 6. (A,B) Pathways enriched in highly cytotoxic intratumoral NK cells in our snRNA-seq dataset (A) and scRNA-seq Steele *et al.* dataset (B) (see *Fig. 6A-C*), shown by Odds ratios (left heatmaps) and significance (right heatmaps; Fisher exact test). DEGs between NK1_c1 and indicated other clusters (columns) were calculated using the presto implementation of the Wilcoxon rank sum test and auROC analysis. Significantly (adj. p val < 0.05) enriched genes were investigated for pathway enrichment by Enrichr using the Hallmark reference gene sets. X indicates pathway was not enriched. AKT, protein kinase B; IL-2, interleukin-2; mTOR(C1), mechanistic target of rapamycin (complex 1); PI3K, phosphoinositide 3-kinase; STAT5, signal transducer and activator of transcription 5; UV, ultraviolet. **(C)** Representative flow cytometry plots showing the gating strategy for NK cell signaling assays in co-culture experiments with MIA PaCa-2 cell lines (see *Fig. 6D,F,G*) **(D)** Representative flow cytometry plots showing gating strategy to analyze intracellular p-mTOR (Ser2448) and p-S6 (Ser235/236) levels in NK cells after co-culture with MIA PaCa-2 cell lines for indicated timepoints, in the presence or absence of IL-2 (see *Fig. 6F,G*). **(E)** Quantification of NK cell p-STAT3 (Tyr705) levels upon exposure to different concentrations of recombinant human (rh) WT-TIMP-1 for different time points as indicated (2 and 5 min: UNT, n = 9; all TIMP-1 concentrations, n = 6; 10 and 30 min: all conditions, n = 6). For MFI calculations in (E), background signals of FMO controls were subtracted and fold-changes were calculated by normalizing to the UNT controls. Statistical significance was calculated compared to respective UNT controls by two-way ANOVA and Dunnett test for multiple comparison correction. STAT3, signal transducer and activator of transcription 3; UNT, untreated. Data in (E) showing biological replicates and represented as box and whiskers plots.

Fig. S7

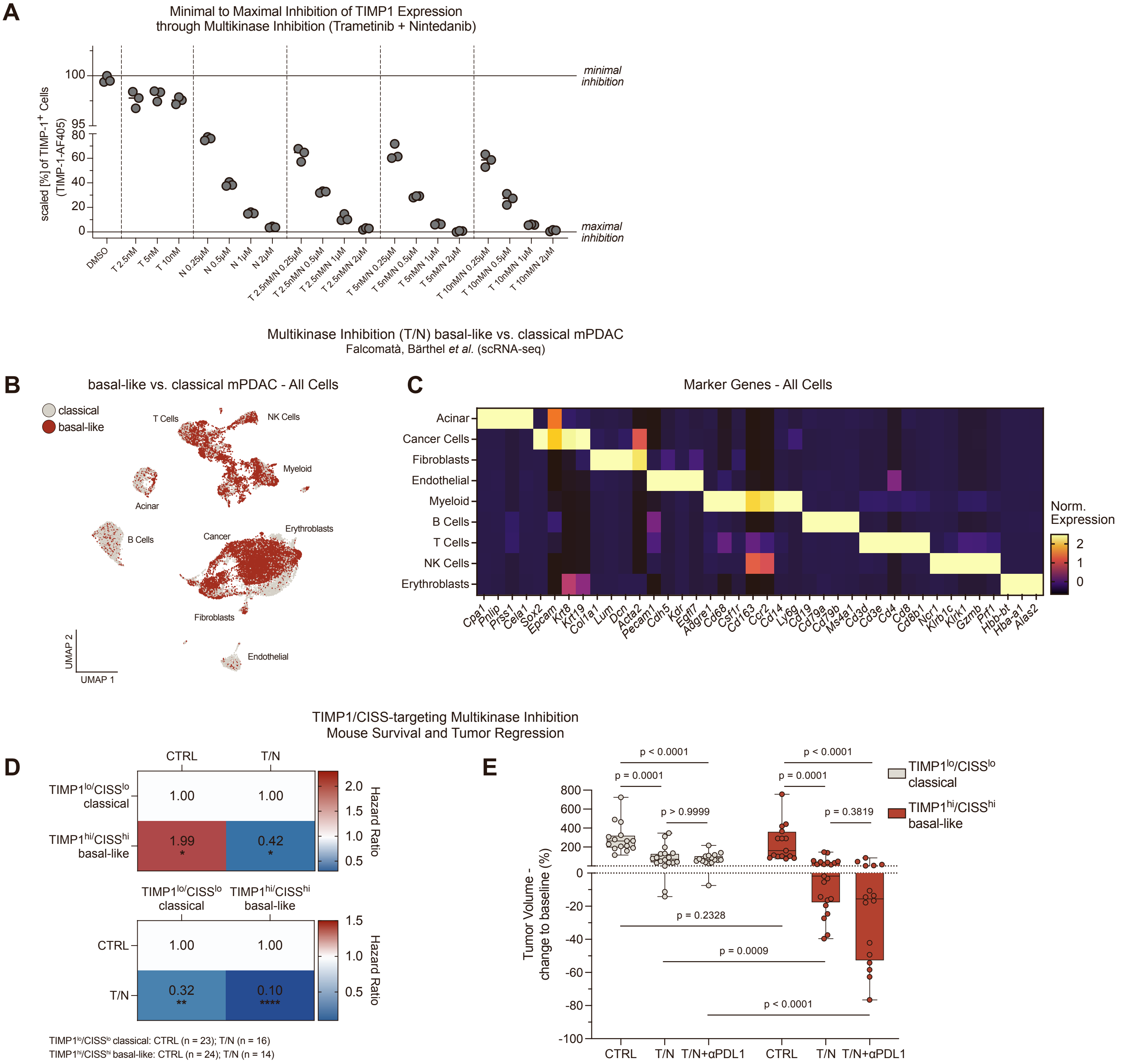
A
Minimal to Maximal Inhibition of TIMP1 Expression through Multikinase Inhibition (Trametinib + Nintedanib)
scaled [%] of TIMP-1+ Cells (TIMP-1-AF405)
minimal inhibition
maximal inhibition
DMSO
T 2.5nM
T 5nM
T 10nM
N 0.25µM
N 0.5µM
N 1µM
N 2µM
T 2.5nM/N 0.25µM
T 2.5nM/N 0.5µM
T 2.5nM/N 1µM
T 2.5nM/N 2µM
T 5nM/N 0.25µM
T 5nM/N 0.5µM
T 5nM/N 1µM
T 5nM/N 2µM
T 10nM/N 0.25µM
T 10nM/N 0.5µM
T 10nM/N 1µM
T 10nM/N 2µM
Multikinase Inhibition (T/N) basal-like vs. classical mPDAC
Falcomatà, Bärthel et al. (scRNA-seq)
B
basal-like vs. classical mPDAC - All Cells
classical
basal-like
T Cells
NK Cells
Myeloid
Acinar
B Cells
Cancer
Erythroblasts
Fibroblasts
Endothelial
UMAP 1
UMAP 2
C
Marker Genes - All Cells
Acinar
Cancer Cells
Fibroblasts
Endothelial
Myeloid
B Cells
T Cells
NK Cells
Erythroblasts
Norm. Expression
Cpa1 Pnlip Prss1 Cela1 Sox2 Epcam Krt8 Krt19 Col1a1 Lum Dcn Acta2 Pecam1 Cdh5 Kdr Egfl7 Adgre1 Cd68 Csf1r Cd163 Ccr2 Cd14 Ly6g Cd19 Cd79a Cd79b Ms4a1 Cd3d Cd3e Cd4 Cd8 Cd8b1 Ncr1 Klrb1c Klrk1 Gzmb Prf1 Hbb-bt Hba-a1 Alas2
TIMP1/CISS-targeting Multikinase Inhibition
Mouse Survival and Tumor Regression
D
CTRL
T/N
TIMP1lo/CISSlo classical
TIMP1hi/CISShi basal-like
1.00
1.00
1.99 *
0.42 *
Hazard Ratio
CTRL
T/N
1.00
1.00
0.32 **
0.10 ****
TIMP1lo/CISSlo classical: CTRL (n = 23); T/N (n = 16)
TIMP1hi/CISShi basal-like: CTRL (n = 24); T/N (n = 14)
E
Tumor Volume - change to baseline (%)
p < 0.0001
p = 0.0001
p > 0.9999
p < 0.0001
p = 0.0001
p = 0.3819
p = 0.2328
p = 0.0009
p < 0.0001
TIMP1lo/CISSlo classical
TIMP1hi/CISShi basal-like
CTRL
T/N
T/N+αPDL1
CTRL
T/N
T/N+αPDL1

Fig. S7 Multikinase Inhibition using Trametinib and Nintedanib in PDAC *in vitro* and *in vivo*, related to Fig. 7. (A) Intracellular TIMP-1 levels in MIA PaCa-2 cells upon treatment with indicated concentrations of MEK inhibitor Trametinib (T) and RTK inhibitor Nintedanib (N), or DMSO control, using flow cytometry (n = 3; biological replicates). [%] of TIMP-1$^+$ cells identified by comparison to respective unstained controls and scaled from minimal to maximal inhibition for identification of drug synergy (see *Fig. 7E*). **(B,C)** UMAP embedding of all cells (B) from mouse pancreas scRNA-seq data ([S10]; see *Fig. 7F,G*), colored by post-hoc cell-type annotations, and selected marker gene expression (C) for annotated cell types. **(D,E)** *In vivo* responses to treatment on mouse survival (D) and tumor regression (E; biological individuals) ([S10]; see *Fig. 7F*). For survival data (D), statistics were performed using logrank Mantel-Cox test of Kaplan-Meier survival curves for indicated comparisons (lower rows vs. upper rows). For tumor regression (E), indicated groups were compared using Kruskal-Wallis tests with Dunn's test (upper) or Mann-Whitney tests (lower).

Supplemental References

S1: Cao, L., Huang, C., Cui Zhou, D., Hu, Y., Lih, T.M., Savage, S.R., Krug, K., Clark, D.J., Schnaubelt, M., Chen, L., et al. (2021). Proteogenomic characterization of pancreatic ductal adenocarcinoma. Cell 184, 5031–5052 e5026. 10.1016/j.cell.2021.08.023.

S2: Abdollahi, A., Schwager, C., Kleeff, J., Esposito, I., Domhan, S., Peschke, P., Hauser, K., Hahnfeldt, P., Hlatky, L., Debus, J., et al. (2007). Transcriptional network governing the angiogenic switch in human pancreatic cancer. Proc Natl Acad Sci U S A 104, 12890–12895. 10.1073/pnas.0705505104.

S3: Moffitt, R.A., Marayati, R., Flate, E.L., Volmar, K.E., Loeza, S.G., Hoadley, K.A., Rashid, N.U., Williams, L.A., Eaton, S.C., Chung, A.H., et al. (2015). Virtual microdissection identifies distinct tumor- and stroma-specific subtypes of pancreatic ductal adenocarcinoma. Nat Genet 47, 1168–1178. 10.1038/ng.3398.

S4: Diaferia, G.R., Balestrieri, C., Prosperini, E., Nicoli, P., Spaggiari, P., Zerbi, A., and Natoli, G. (2016). Dissection of transcriptional and cis-regulatory control of differentiation in human pancreatic cancer. EMBO J 35, 595–617. 10.15252/embj.201592404.

S5: Steele, N.G., Carpenter, E.S., Kemp, S.B., Sirihorachai, V.R., The, S., Delrosario, L., Lazarus, J., Amir, E.D., Gunchick, V., Espinoza, C., et al. (2020). Multimodal Mapping of the Tumor and Peripheral Blood Immune Landscape in Human Pancreatic Cancer. Nat Cancer 1, 1097–1112. 10.1038/s43018-020-00121-4.

S6: Netskar, H., Pfefferle, A., Goodridge, J.P., Sohlberg, E., Dufva, O., Teichmann, S.A., Brownlie, D., Michaelsson, J., Marquardt, N., Clancy, T., et al. (2024). Pan-cancer profiling of tumor-infiltrating natural killer cells through transcriptional reference mapping. Nat Immunol 25, 1445–1459. 10.1038/s41590-024-01884-z.

S7: Crinier, A., Milpied, P., Escaliere, B., Piperoglou, C., Galluso, J., Balsamo, A., Spinelli, L., Cervera-Marzal, I., Ebbo, M., Girard-Madoux, M., et al. (2018). High-Dimensional Single-Cell Analysis Identifies Organ-Specific Signatures and Conserved NK Cell Subsets in Humans and Mice. Immunity 49, 971–986 e975. 10.1016/j.immuni.2018.09.009.

S8: Collins, P.L., Cella, M., Porter, S.I., Li, S., Gurewitz, G.L., Hong, H.S., Johnson, R.P., Oltz, E.M., and Colonna, M. (2019). Gene Regulatory Programs Conferring Phenotypic Identities to Human NK Cells. Cell 176, 348–360 e312. 10.1016/j.cell.2018.11.045.

S9: Smith, S.L., Kennedy, P.R., Stacey, K.B., Worboys, J.D., Yarwood, A., Seo, S., Solloa, E.H., Mistretta, B., Chatterjee, S.S., Gunaratne, P., et al. (2020). Diversity of peripheral blood human NK cells identified by single-cell RNA sequencing. Blood Adv 4, 1388–1406. 10.1182/bloodadvances.2019000699.

S10: Falcomatà, C., Bärthel, S., Widholz, S.A., Schneeweis, C., Montero, J.J., Toska, A., Mir, J., Kaltenbacher, T., Heetmeyer, J., Swietlik, J.J., et al. (2022). Selective multi-kinase inhibition sensitizes mesenchymal pancreatic cancer to immune checkpoint blockade by remodeling the tumor microenvironment. Nat Cancer 3, 318–336. 10.1038/s43018-021-00326-1.
