# Supplementary material for: Multimodal profiling of pancreatic cancer reveals a TIMP-1-dominated secretory profile determining pro-tumor immunoinstruction in human cancers
Source: Cell Rep Med. 2026 Jan 20;7(1):102546. doi: 10.1016/j.xcrm.2025.102546 (PMC12866174; doi:10.1016/j.xcrm.2025.102546)
Supplement: Document S2. Article plus supplemental information [file mmc5.pdf]

Cell Reports
Medicine

Article

Multimodal profiling of pancreatic cancer reveals a TIMP-1-dominated secretory profile determining pro-tumor immunoinstruction in human cancers

Graphical abstract

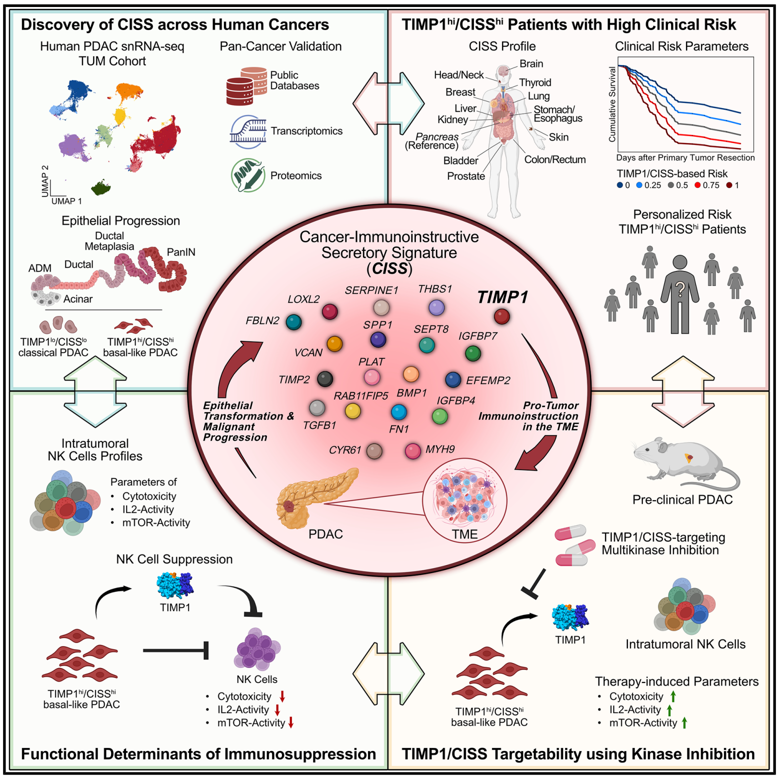

Highlights

- A secretory profile (CISS) links malignant evolution to immunosuppression in cancer
- In PDAC, CISS arises in pre-malignancy and culminates in TIMP-1^{hi} basal-like cancer
- PDAC-cell-mediated NK cell suppression is TIMP-1-dependent
- TIMP1/CISS identify high-risk patients and prove targetable in pre-clinical PDAC

Authors

Julian Frädrich, Carmen Mota Reyes, Michel Hendel, ..., Dieter Saur, Ihsan Ekin Demir, Achim Krüger

Correspondence

achim.krueger@tum.de

In brief

By multiomic profiling across human cancers, Frädrich et al. discover a secretory profile linking malignant evolution to immunosuppression and poor patient survival. In pancreatic cancer, this profile is shaped by TIMP-1-dependent NK cell suppression and proves targetable through clinically approved kinase inhibitors, highlighting exploitable prognostic and therapeutic potential.

Frädrich et al., 2026, Cell Reports Medicine 7, 102546
January 20, 2026 © 2025 The Author(s). Published by Elsevier Inc.
https://doi.org/10.1016/j.xcrm.2025.102546

CellPress

Cell Reports Medicine

CellPress
OPEN ACCESS

Article

Multimodal profiling of pancreatic cancer reveals a TIMP-1-dominated secretory profile determining pro-tumor immunoinstruction in human cancers

Julian Frädrich,[1] Carmen Mota Reyes,[2,3,4,5] Michel Hendel,[1] Vanessa Brunner,[1] Batu Toledo,[1] Damjan Manevski,[1] Alexander Sommer,[1] Daniel Häußler,[1] Dominik Beck,[1] Daniele Lucarelli,[6,7] Jaime Martínez de Villareal,[8,9] Lennard Halle,[7] Raphael Kfuri-Rubens,[7] Kaan Çifcibaşı,[2] Anna Hirschberger,[10] Rupert Öllinger,[11] Percy A. Knolle,[10] Katja Steiger,[12] Roland Rad,[11] Fabian J. Theis,[7,13,14] Francisco X. Real,[8,9,15] Stefanie Bärthel,[6,16,17] Jan P. Böttcher,[10,18,19] Dieter Saur,[3,6,16] Ihsan Ekin Demir,[2,3,4,20] and Achim Krüger[1,21,*]

[1]TUM School of Medicine and Health, Institute of Experimental Oncology and Therapy Research, Technical University of Munich, Munich, Germany
[2]TUM School of Medicine and Health, Department of Surgery, Technical University of Munich, Munich, Germany
[3]German Cancer Consortium (DKTK), Partner Site Munich, Munich, Germany
[4]Neural Influences in Cancer (NIC), International Research Consortium, Munich, Germany
[5]Bayerisches Zentrum für Krebsforschung (BZKF), Munich, Germany
[6]TUM School of Medicine and Health, Center for Translational Cancer Research (TranslaTUM), Technical University of Munich, Munich, Germany
[7]Institute of Computational Biology, Computational Health Center, Helmholtz Munich, Neuherberg, Germany
[8]Epithelial Carcinogenesis Group, Spanish National Cancer Research Centre, Madrid, Spain
[9]Centro Nacional de Investigaciones Oncólogicas (CNIO), 28029 Madrid, Spain
[10]TUM School of Medicine and Health, Institute of Molecular Immunology, Technical University of Munich, Munich, Germany
[11]TUM School of Medicine and Health, Institute of Molecular Oncology and Functional Genomics, Technical University of Munich, Munich, Germany
[12]TUM School of Medicine and Health, Department of Pathology, Technical University of Munich, Munich, Germany
[13]TUM School of Computation, Information and Technology, Department of Mathematics, Technical University of Munich, Garching, Germany
[14]TUM School of Life Sciences, Weihenstephan, Technical University of Munich, Freising, Germany
[15]Department of Medicine and Life Sciences, Universitat Pompeu Fabra, 08003 Barcelona, Spain
[16]Division of Translational Cancer Research, German Cancer Research Center and German Cancer Consortium, Heidelberg, Germany
[17]BioMed X Institute Heidelberg, Heidelberg, Germany
[18]Department of Experimental Immunology, Institute of Immunology, University of Tübingen, Tübingen, Germany
[19]M3 Research Center, University Hospital Tübingen, University of Tübingen, Tübingen, Germany
[20]Else Kröner Clinician Scientist Professor for Translational Pancreatic Surgery, Technical University of Munich, Munich, Germany
[21]Lead contact
*Correspondence: achim.krueger@tum.de
https://doi.org/10.1016/j.xcrm.2025.102546

SUMMARY

The immunosuppressive tumor microenvironment (TME) fosters cancer progression, yet overarching determinants of cancer-borne immunoinstruction remain ill-defined. By multimodal integration of single-nucleus and bulk transcriptomics, proteomics, functional approaches, and clinical parameters, we discover a cancer-immunoinstructive secretory signature (CISS) across multiple human cancers—a set of inflammatory proteins correlated with poor prognosis and pro-tumorigenic TMEs. In pancreatic cancer (PC), CISS arises in pre-malignant epithelium, intensifies along transformation toward most malignant basal-like PC, and particularly correlates with suppressed natural killer (NK) cell activity. The CISS is quantitatively dominated by tissue inhibitor of metalloproteinases (TIMP)-1, most prevalent in TIMP-1^{hi}/CISS^{hi} basal-like PC, and causal for PC-cell-mediated NK cell suppression, reflected by impaired cytotoxicity, interleukin-2 (IL-2) responses, and mammalian target of rapamycin (mTOR) signaling. In pre-clinical PC, TIMP-1/CISS proves targetable through combined inhibition of upstream kinases with clinically approved drugs trametinib and nintedanib. Collectively, CISS represents a ubiquitous signature of pro-tumor immunoinstruction with actionable diagnostic and therapeutic potential across human cancers.

© 2025 The Author(s). Published by Elsevier Inc.
This is an open access article under the CC BY license (http://creativecommons.org/licenses/by/4.0/).

INTRODUCTION

In cancer, an immunosuppressive tumor microenvironment (TME) is central to malignant progression.[1–3] Cancer cells can profoundly reprogram tumor-infiltrating immune cells, such as cytotoxic CD8$^+$ T cells and natural killer (NK) cells, enabling evasion of tumor immune control,[4] a well-known hallmark of cancer.[5] Immunosuppression within the TME can occur through juxtacrine immune checkpoints via cell-cell contact or through paracrine signaling involving secreted factors, such as cytokines.[6,7] Despite the clinical success of immune checkpoint-blockade (ICB) in some cancers,[8] many of the deadliest malignancies, such as pancreatic cancer (PC), are markedly resistant to current, mostly T-cell-centered, immunotherapies.[8,9] Therefore, as recently emphasized by D. Hanahan et al., it is crucial to unravel immunoregulatory drivers of paracrine TME reprogramming,[7] trace their emergence during cancer progression, and determine how their expression correlates with adverse clinical outcomes and defines actionable therapeutic targets.

A cancer exemplifying this unmet clinical need is pancreatic ductal adenocarcinoma (PDAC), the most common form of PC[10] and a leading cause of cancer-related deaths.[11] The profound immunosuppression in PDAC[2,12–14] originates at early neoplastic transformation,[15] comprising acinar-to-ductal metaplasia (ADM) and pancreatic intraepithelial neoplasia (PanIN),[16] a precursor of invasive cancer. Despite its almost universal lethality,[17] emerging evidence highlight that PDAC progression and mortality gradually depend on inter- and intratumoral heterogeneity.[14] This includes heterogeneous PDAC cells,[18–20] broadly classified into two subtypes: classical epithelial-like PDAC cells retaining pancreatic lineage gene expression and basal-like PDAC cells that lose epithelial identity, adopt mesenchymal traits, and display marked aggressiveness and therapy resistance.[14,19,21] While recent studies have advanced our understanding of the cellular and molecular landscape in PDAC[22–24] and clarified the genomic basis of its heterogeneity,[25] accumulating evidence point to non-genomic, environmental cues, such as inflammation, as critical for transformation and malignant progression.[26–28] However, how these cues shape immunosuppressive factors arising during PDAC progression, how these factors depend on cancer heterogeneity, and whether they represent therapeutic targets remain poorly understood.

To address this gap, we implemented a multimodal strategy across human and murine pancreata, integrating multiomic profiling with functional approaches and clinical analyses. We discovered a cancer-immunoinstructive secretory signature (CISS) that emerged upon epithelial inflammation, intensified along transformation toward most malignant basal-like cancer, and correlated with immunosuppressive TMEs across human cancers, particularly marked by NK cell suppression. We elucidate TIMP-1 as the dominant CISS factor, functionally causal for PDAC-cell-mediated suppression of NK cell cytotoxicity, interleukin (IL)-2 responses, and mechanistic target of rapamycin (mTOR) signaling. Toward clinical translation, we show that TIMP-1 and CISS are targetable in PDAC through combined inhibition of upstream kinases with the clinically approved drugs trametinib and nintedanib *in vivo*. Our findings contribute to understanding immunosuppression as an early and evolving capacity of epithelial cells during cancer progression and establish a mechanistically defined, therapeutically actionable axis of pro-tumor immunosuppression with implications across human cancers.

RESULTS

Inflammation drives a cancer-progression-related secretory profile in the pancreatic epithelium

Given that pancreatic inflammation is crucial for PDAC initiation and progression,[26,27,29] we first investigated whether inflammation-induced transcriptional profiles in pancreatic epithelial cells are recapitulated upon PDAC development (Figures 1A–1F). Mining of RNA sequencing (RNA-seq) data[26,27] of epithelial cells isolated from pancreatitis- or PDAC-afflicted mice and respective controls identified 432 significantly and persistently upregulated genes, conserved across disease conditions (Figure 1A). Gene set enrichment analysis (GSEA) revealed that this transcriptional pattern mostly reflected processes such as wound healing, cellular development and differentiation, and tissue remodeling (Figure 1B; Table S1). Considering the importance of secreted factors in orchestrating such processes, we next focused on genes encoding secreted proteins within this profile. We identified 36 consistently upregulated secreted factors (Figure 1A)—including extracellular matrix modulators (e.g., fibronectin, fibulin-2, and versican), inflammation-associated chemokines and cytokines (e.g., CXCL16, transforming growth factor β1 [TGF-β1], and tissue inhibitor of metalloproteinase 1 [TIMP-1]), and stress-response mediators (e.g., Annexin2, and HSP90B) (Figures 1C–1F).

Addressing these changes during malignant evolution in PDAC, we then performed bulk RNA-seq on tumors (n = 24) from genetically engineered PDAC-bearing KPC (Pdx-1$^{+/Cre}$; Kras$^{+/LSL-G12D}$; Trp53$^{+/LSL-R172H}$) mice,[30] including early to advanced (grades 2, 3, and 4) PDAC, and healthy controls (n = 11) (Figure 1G; Table S2). This showed modest induction of the 36-factor pattern in early tumorigenesis and prominent, progression-dependent elevation in high-grade tumors (Figures 1H and 1I). Collectively, these analyses identified an epithelial secretory profile that stems from pancreatic inflammation and closely aligns with malignant PDAC progression.

A secretory signature in human cancers correlates with pro-tumor immune regulation and poor patient survival

Translating this mouse-model-derived profile to patients, we analyzed RNA-seq data from The Cancer Genome Atlas (TCGA) PC cohort (TCGA-PAAD, n = 178) and independently validated its protein levels (Cao Cohort,[31] n = 140). Among the 36 factors, we identified a subcluster of 19 distinctively co-expressed proteins in both cohorts (Figures 2A, 2B, S1A, and S1B), also correlating markedly stronger with PDAC progression in mice than the remaining factors (Figure S1C). Protein levels of all 19 factors were robustly enriched in patient tumor tissues compared to tumor-adjacent areas or normal control pancreata (Figure 2C). Importantly, elevation of this 19-factor signature correlated significantly with poor patient survival (Figure 2D), suggesting a central disease-promoting role. In support, GSEA and CIBERSORTx

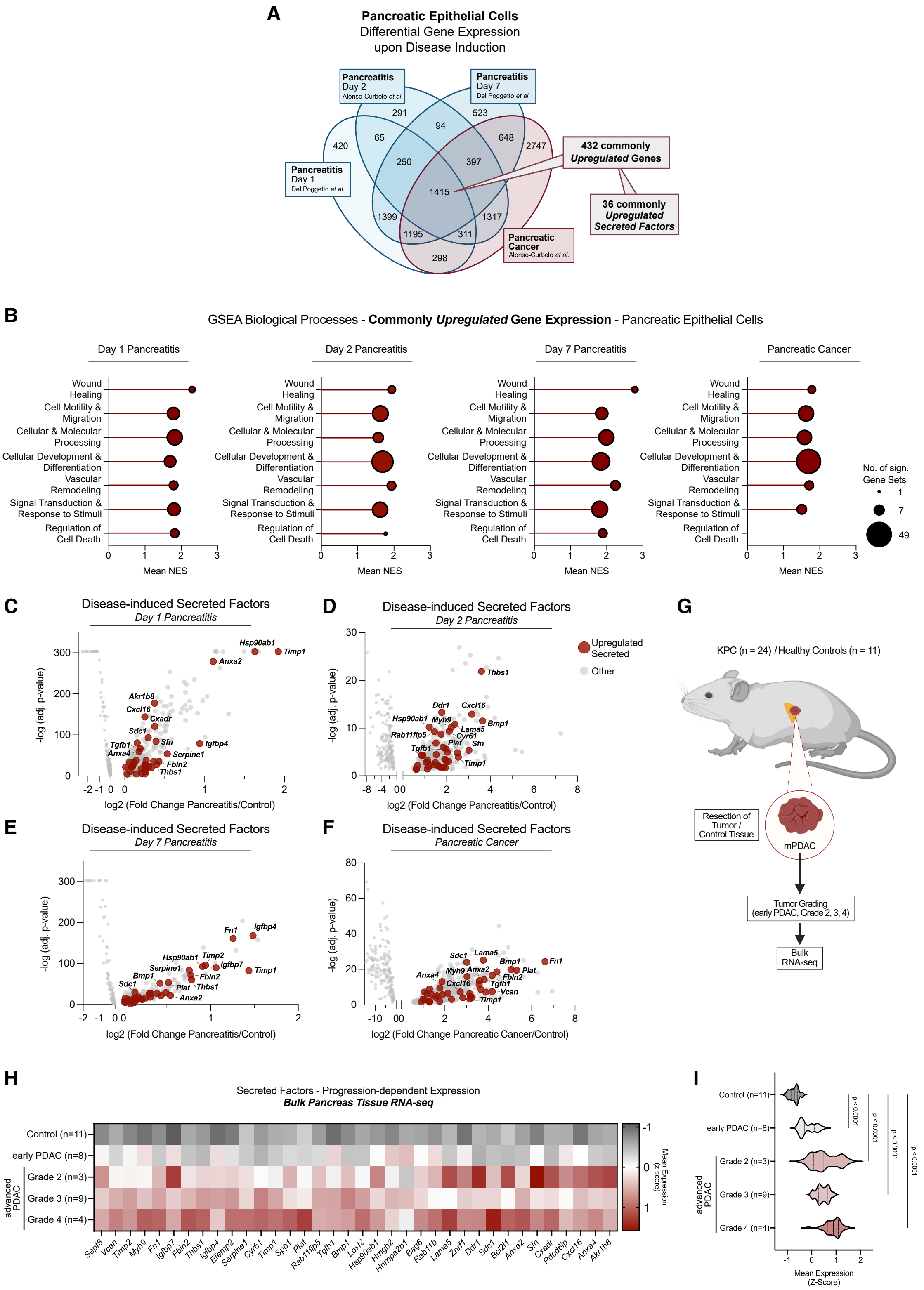

Figure 1. Inflammation drives a cancer-progression-related secretory profile in the pancreatic epithelium

(A) Venn diagram of differentially expressed genes (disease condition vs. healthy controls), commonly upregulated genes, and commonly upregulated secreted factors in pancreatic epithelial cells from pancreatitis- or PDAC-bearing mice.[26,27]

(legend continued on next page)

analysis revealed that patients with high signature expression showed transcriptional profiles associated with immune cell regulation, tissue environment remodeling (Figure 2E), and a high myeloid-to-lymphoid ratio (Figure 2F), all of which are central for the pro-tumor TME in highly aggressive PC.[2,12–14]

Toward generalizing this pattern across human cancers, we examined various entities from the TCGA cohorts, including 10 epithelial (e.g., colorectal, kidney, and breast) and 2 non-epithelial cancers (glioblastoma and melanoma) (Figure 2G). Across entities, the 19 factors correlated robustly and showed immune cell patterns similar to those in PC (Figures 2H, S1D, and S2), most significantly reflected by suppressed NK cell activity (Figures 2I and S1D), essential for cancer immune surveillance,[3,32,33] and increase in potentially pro-tumor myeloid cells, such as M2-like macrophages[34] and neutrophils[35] (Figures 2I and S1D). Importantly, elevated signature expression was associated with poor patient survival across entities, with particularly high and significant correlations in pancreatic, colorectal, kidney, and brain cancer (Figure 2J). Collectively, the secretory pattern was consistently linked to pro-tumor immunoinstruction and poor patient survival across cohorts. Thus, we designated this 19-factor profile as a CISS in human cancers.

snRNA-seq identifies epithelial-subtype-specific CISS upregulation toward TIMP-1^{hi} basal-like cancer PDAC

To further dissect the CISS during PDAC progression, we performed single-nucleus RNA-seq (snRNA-seq) on primary tumor samples from 17 treatment-naïve PDAC patients (TUM Cohort) (Figure 3A; Table S3). We recovered 183,666 high-quality nuclei, which clustered into nine major cell types: non-malignant and malignant epithelial cells, myeloid and lymphoid immune cells, endocrine cells, endothelial cells, cancer-associated fibroblasts, vascular smooth muscle cells, and pericytes (Figures 3A and 3B). These major cell types were well distributed across samples (Figure S3A) and comprised multiple subsets, including diverse stromal and immune cell populations (Figures 3C and S3B–S3F), consistent with previous single-cell reports in human PDAC.[22–24]

The largest cell-type cluster encompassed 90,343 nuclei from the epithelial compartment, including heterogeneous non-malignant epithelial subsets (Figures 3D and 3E). These ranged from acinar to metaplastic *CRP*^{hi}/*ONECUT2*^{hi} ductal cells (Ductal_c2)[24,36]—putatively a major source of malignancy in PDAC[24,37]—and PanIN-like cells.[24] We further retrieved transitional ADM clusters sharing acinar and ductal gene expression (Figure 3E), which highlighted ongoing epithelial plasticity within pancreatic tumors, as supported by RNA velocity analysis (Figure 3F). To dissect cancer cell subtypes, we confirmed malignant status by copy-number variations (Figure S3G) and grouped 13 unsupervised Louvain clusters into six distinct cancer cell clusters based on inferred cell state progression, residual pancreatic lineage genes, increasing cancer markers (*KRT7*, *KRT19*, *MUC1*, and *S100A6*), and cell-subtype-specific genes (Figures 3D–3G).[24,25,38] These clusters ranged from classical cancer (cancer clusters 1 and 2) to most progressed basal-like cancer cells (cancer clusters 3, 4, 5, and 6) (Figure 3D).

Within the heterogeneous epithelium, CISS was robustly upregulated across the entire progression spectrum and most prominently enriched in basal-like PDAC cells (Figures 3H and S3H). Moreover, we observed cluster-specific distribution of the 19 CISS factors, with distinct prevalences across epithelial subtypes (Figures 3I and S3H). While some factors, such as *SPP1*, were strongly linked to pre-malignant epithelial transitions (e.g., ADM and Ductal_c2), most were enriched in neoplastic and malignant epithelium. Within these, we found factors universally and subtype-independently elevated in cancer (e.g., *VCAN*), factors more abundant in classical cancer (e.g., *TGFB1*, *BMP1*, and *PLAT*), and those predominantly expressed in basal-like cancer (e.g., *TIMP1* and *IGFBP4*).

Among these, the immunoregulatory cytokine TIMP-1[39] stood out by strongest upregulation relative to acinar cells, significantly increased prevalence upon malignant transformation, and particularly high levels in most progressed basal-like Cancer_c5 and Cancer_c6 (hereafter referred to as TIMP-1^{hi} basal-like cancer cells) (Figures 3I–3K). Importantly, this progressive rise in *TIMP1* expression toward basal-like cancer was consistent across all patients (Figure 3L), identifying TIMP-1 as a robust marker of PDAC progression and most prevalent CISS factor.

CISS-prevalent TIMP-1 is causal for PDAC-cell-induced NK cell suppression

To establish a functional link between CISS upregulation and immunosuppression in the TME, we performed principal-component analysis (PCA) to integrate epithelial and immune cell composition with CISS and *TIMP1* expression across tumor samples (Figures 4A–4C). While most variance (PC1: 38.54%) reflected general differences in epithelial and immune composition, indicating variable immune infiltration across tumors (Figures 4A and 4B), basal-like PDAC positioned significantly closer to immune cells in the PCA and was clearly distinct from classical cancer (Figures 4A–4C). This suggested subtype-dependent interactions between epithelial and immune cells, supported by co-positioning of epithelial CISS and *TIMP1* between both compartments (Figure 4A). In fact, epithelial *TIMP1* significantly correlated with intratumoral NK cell presence (Figure 4B), indicating cancer-NK cell interactions linked to epithelial *TIMP1* upregulation (Figures 4A–4C). Supporting this

(B) GSEA of biological processes positively correlated with the 432 commonly upregulated genes across conditions. Genes were ranked by fold-changes (disease vs. control). Reference gene set: GO: Biological Processes. Positively (nom. $p < 0.05$) enriched gene sets were categorized (see Table S1).
(C–F) Volcano plots of the 36 commonly upregulated secreted factors (A) per condition.
(G) Workflow for tissue collection, tumor grading, and bulk RNA-seq of murine KPC PDAC tumors (early PDAC [$n = 8$], grade 2 [$n = 3$], grade 3 [$n = 9$], grade 4 [$n = 4$]) and controls ($n = 11$).
(H and I) Normalized gene expression of the 36 secreted factors (A, C–F) across conditions (G). Statistics in (I) by one-way ANOVA for matched data (genes [H]) and Dunnett test. Data presented as individual points (C–F) or as violin plots (I). (G) Created with BioRender.com.

CellPress
OPEN ACCESS

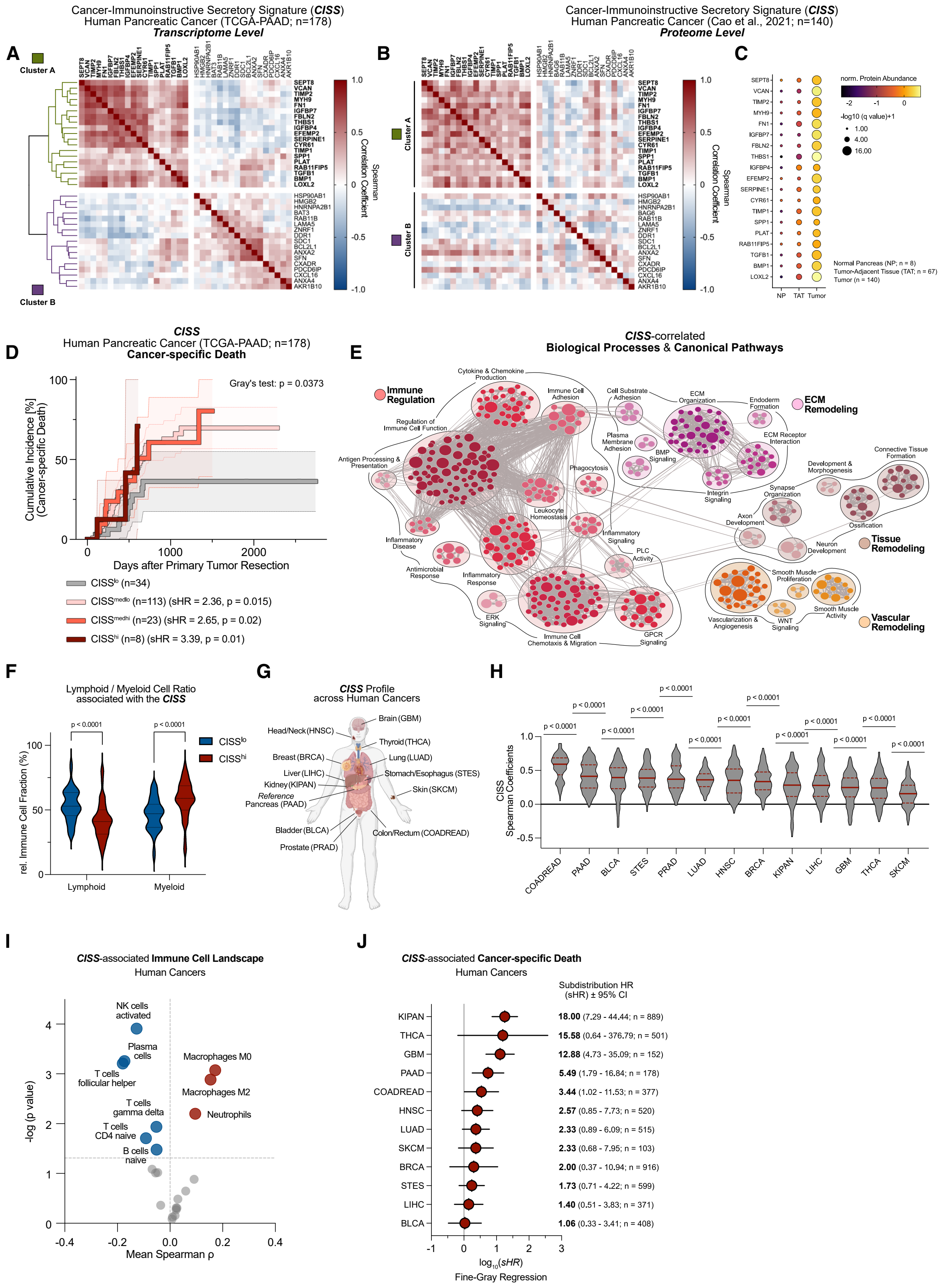

(legend on next page)

notion in TCGA-PAAD bulk RNA-seq data (Figure 4D), *TIMP1*-correlated gene expression was most strongly linked to "*immunoregulatory interactions between lymphoid and non-lymphoid cells*" (Figure 4E), and CIBERSORTx analysis showed most significant correlation with NK cell suppression in pancreatic tumors (Figure 4F). This was validated in an independent PC cohort[40] also including chronic pancreatitis patients and normal pancreas controls (Figures S4A and S4B), implying that even in chronic inflammation, which may precede PDAC,[41] *TIMP1* upregulation is linked to suppressed NK cell activity.

To test causality of TIMP-1 for PC-cell-mediated NK cell suppression, we then screened human PC cell line transcriptome data[19,42] to identify a model resembling TIMP-1^{hi} basal-like cancer (Figures 3I–3L and 4C). We identified MIA PaCa-2 cells as most suitable based on their mesenchymal/basal-like properties,[19,43] particularly high *TIMP1* (Figure S4C) and broad CISS factor expression (Figure S4D), and NK cell-suppressive activity mediated by secreted factors (Figures 4G–4I). We then generated two independent TIMP-1 knockout lines and a CRISPR control line from wild-type (WT) MIA PaCa-2 cells (Figures 4J, S4E, and S4F). RNA-seq of these cell lines and subsequent GSEA (Figures 4J–4L) revealed TIMP-1-dependent suppression of immune-defense-related genes, including innate immune responses, cytokine and interferon signaling, and cell killing (Figures 4M and S4G). Finally, primary NK cell exposure to PDAC-cell-line-derived supernatants, followed by killing assays with K562 targets (Figures 4N and S5A), showed that TIMP-1-deficient cells were significantly less NK-cell-suppressive (Figure 4O,P). Collectively, this demonstrated TIMP-1 as causal for PDAC-cell-mediated NK cell suppression, consistent with transcriptional patterns observed in human tumors.

CISS-prevalent TIMP-1 is sufficient to suppress cytotoxic capacity in NK cells via CD74 signaling

We next profiled intratumoral NK cells in our cohort and cross-validated with external human PDAC scRNA-seq data (Steele Cohort[22]) (Figures 5A–5E and S5B–S5E) to elucidate molecular mechanisms of TIMP-1-dependent NK cell suppression. In both cohorts, we identified eight unsupervised NK clusters, consolidating into four major clusters with distinct gene expression patterns. This included CD56^{dim} NK cells (ctx^{hi}) with high cytotoxic effector expression (granzyme B [*GZMB*] and perforin [*PRF1*]) and other cytotoxic markers (e.g., *FCGR3A*[44] and *TBX21*[45]) (Figures 5C–5E and S5B–S5E). Other intratumoral NK cells comprised an intermediate *EIF3G*^{hi}/*GZMK*^{hi} cluster with decreased cytotoxic markers (ctx^{lo}),[44,46] a cluster with high expression of regulatory receptors ($KLRC^{hi}$; e.g., *TIGIT*, *KLRC1*, and *KLRC2*), and a CD56^{bright} cluster (high for stem-like markers *TCF7*, *RUNX2*, *IL7R*, and *CCR7*) (Figures 5C, 5D, and S5B–S5E). RNA velocity analysis revealed that, while the $KLRC^{hi}$ cluster was transcriptionally rather unique, the $CD56^{bright}$, ctx^{hi}, and ctx^{lo} clusters showed interconnected transcriptional dynamics (Figure 5F). This included direct trajectories from the $CD56^{bright}$ cluster toward both ctx^{hi} and ctx^{lo} cells, as well as trajectories from ctx^{hi} toward ctx^{lo} NK cells, accompanied by a gradual decline in cytotoxicity markers and upregulated immune checkpoints (e.g., *PD1*, *CTLA4*, and *LAG3*) (Figures 5C–5F).

Exposing NK cells to recombinant human TIMP-1 (rhTIMP-1), we observed that rhTIMP-1 was sufficient to dose-dependently suppress cytotoxic effectors GZMB and perforin (Figure 5G). Importantly, this required pathophysiologically elevated levels of TIMP-1, as observed in human PDAC,[47,48] whereas physiological levels[47,48] were not suppressive, as reported previously.[49] Moreover, it relied on the non-canonical cytokine function of TIMP-1 rather than its canonical anti-proteolytic function, as a rhTIMP-1 variant (rhT2G-TIMP-1) deficient in anti-proteolytic activity[50] retained the NK-inhibitory effect (Figure S5F), whereas broad-spectrum matrix metalloprotease inhibitor batimastat did not (Figure S5G). To pinpoint the involved TIMP-1 receptor, we employed a rhTIMP-1 variant lacking the C-terminal domain (rhN-TIMP-1), signaling exclusively through CD74.[51,52] rhN-TIMP-1 retained suppressive activity, and CD74 blocking with the monoclonal antibody milatuzumab abolished the effect of both rhWT-TIMP-1 and rhN-TIMP-1 (Figure 5H).

Figure 2. A secretory signature in human cancers correlates with pro-tumor immune regulation and poor patient survival

(A and B) Spearman correlation of the 36 secreted factors on transcriptomic [(A), TCGA-PAAD, n = 178] and proteomic level [(B), Cao Cohort,[31] n = 140] in PDAC patient tumors. Hierarchical clustering of coefficients (A) identifies a subset of 19 factors within the 36-gene pattern.

(C) CISS protein levels in normal pancreas (n = 8), tumor-adjacent tissue (n = 67), and PDAC tumors (n = 140) by median-normalized log2 protein levels. Statistics: Mann-Whitney tests (with two-stage step-up method by Benjamini, Krieger, and Yekutieli; FDR q = 0.01) between NP vs. TAT and Tumor vs. TAT.

(D) Cancer-specific death of PC patients (TCGA-PAAD) stratified by CISS expression (0–1 by 25% increments; CISS^{lo} = 34; CISS^{medlo} = 113; CISS^{medhi} = 23; CISS^{hi} = 8). Cumulative incidences (±95% confidence interval [CI]) compared by Gray's test, subdistribution hazard ratios (sHR) vs. CISS^{lo} group by Fine-Gray regression.

(E) GSEA identified CISS correlating with immune regulation, extracellular matrix (ECM) remodeling, tissue remodeling, and vascular remodeling in TCGA-PAAD tumors. DEGs between high (top 25%; n = 45) and low CISS-expressing (bottom 25%; n = 45) tumors by Mann-Whitney tests [corrected for multiple comparisons as in (C)]. Reference gene sets: GO:BP and C2:CP. Upregulated gene sets (NES $\geq$1.75, FDR q $\leq$ 0.05) visualized by enrichment map. ('Node" sizes indicate no. of genes per gene set; "edges" indicate overlapping genes between nodes. BMP, bone morphogenic proteins; ERK, extracellular-signal regulated kinases; GPCR, G-protein-coupled receptors; PLC, phospholipase C.

(F) CIBERSORTx-based correlation between CISS and immune cell types. Statistics by Mann-Whitney tests.

(G) Analysis of CISS profile across TCGA cohorts (see Figure S2).

(H) Spearman correlation [as in (A)] of CISS factors showed significant positive correlation across entities. Statistics by Wilcoxon signed-rank test (vs. zero baseline).

(I) Volcano plot of correlations between CISS and immune cell types by one-sample t tests (vs. zero baseline; see Figure S1D).

(J) Cancer-specific death of TCGA patients stratified by CISS expression. Survival probabilities by Fine-Gray regression (log10 sHR ±95% CI). Data presented as individual points (I) or violin plots (F and H). (G) Created with BioRender.com. (*Of note: Designation of the 19 factors as the cancer-immunoinstructive secretory signature [CISS] is based on phenotypic and functional analyses in* Figures 2E–2I, 3, 4, 5, 6, and 7).

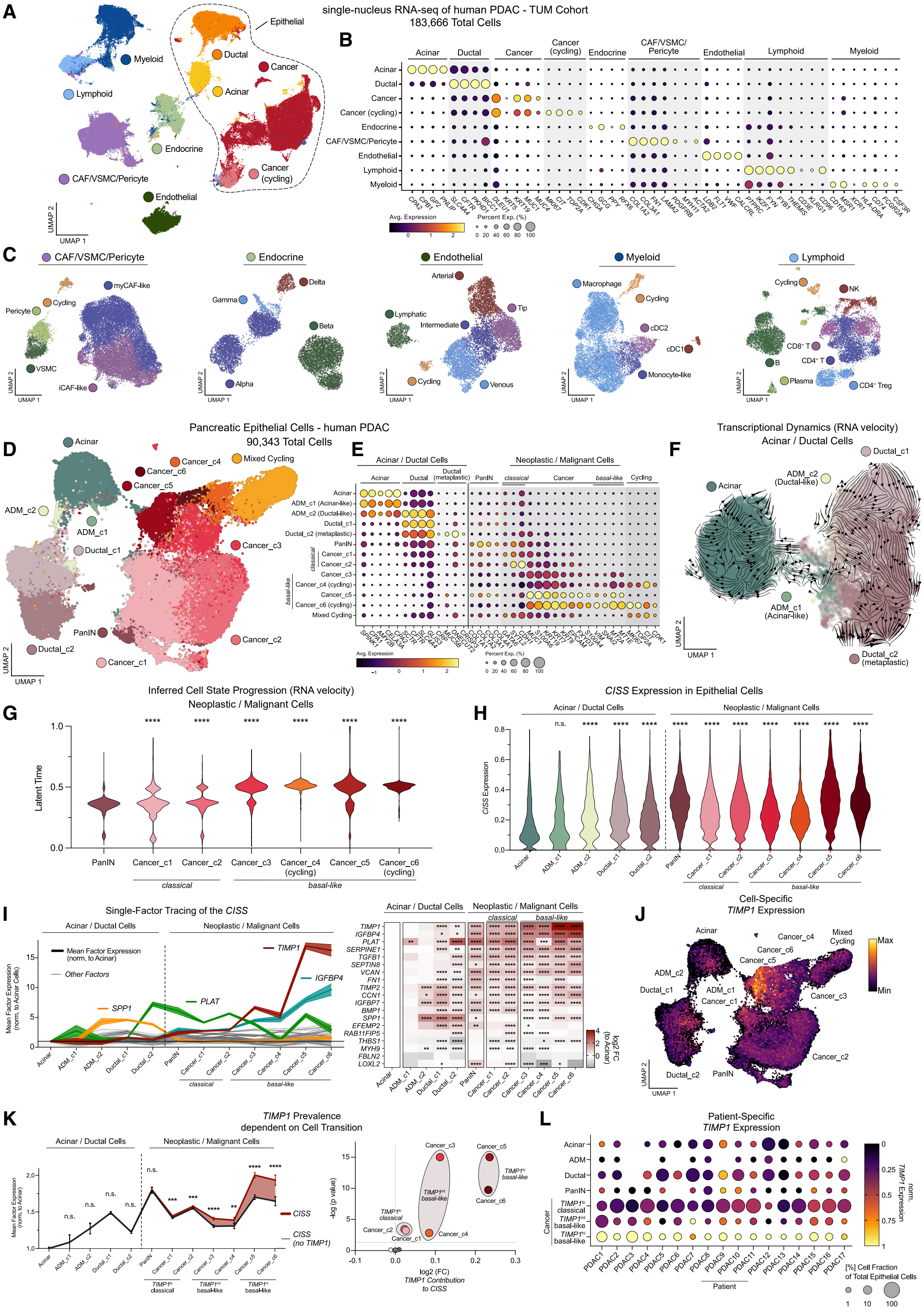

(legend on next page)

Prompted by these mechanistic insights, we examined how the different NK cell clusters relate to malignant epithelial progression in our snRNA-seq dataset. Across the six patients with matched NK and epithelial cell data, individual cell subtypes were uniformly present but varied in frequencies, reflecting marked intra- and intertumoral heterogeneity (Figures 5J–5M). This included considerable differences in ctx^{hi} NK cells, ranging from >50% in *PDAC10* and *PDAC14* to ~16% in *PDAC1* (Figure 5J). Intriguingly, presence of ctx^{hi} NK cells was inversely correlated with TIMP-1^{hi} basal-like cancer (Figure 5M), accounting for <2% of epithelial cells in *PDAC10* and *PDAC14* but up to ~24% in *PDAC1* (Figure 5K). Moreover, PCA-based integration of cell fractions with cancer cell *TIMP1* expression and NK cell *CD74* expression (Figure 5L) showed a substantial contribution of cancer cell *TIMP1* expression to overall tumor heterogeneity, largely driven by the imbalance between TIMP-1^{lo} classical and TIMP-1^{int}/TIMP-1^{hi} basal-like cancer (PC1: 42.68%). Consistently, high NK cell *CD74* expression and ctx^{lo} NK cells were clearly associated with basal-like PDAC, whereas ctx^{hi} NK cells clustered with less-progressed TIMP-1^{lo} classical cancer and pre-malignant epithelium (Figure 5L). Strikingly, CellChat-based inference of cancer cell-to-NK cell signaling activity revealed that, although TIMP-1^{lo} classical cancer cells were the most abundant epithelial cell type (Figures 3L and 5K), TIMP-1^{int} and especially TIMP-1^{hi} basal-like cancer exhibited markedly stronger signaling activity (including TIMP-1/CD74 signaling) toward NK cells (Figure 5N). This was not confined to *PDAC1* with a relatively large basal-like population (Figure 5K) but consistent across patients (Figure S5H). Together, these findings underscore CD74 signaling as essential for TIMP-1-induced suppression of NK cell cytotoxic potential, which in turn emerged as a major determinant of heterogeneous intratumoral NK cells.

TIMP-1-dependent suppression of NK cell mTOR signaling links PDAC immunosuppression to clinical risk profiles

Next, we determined signaling pathways associated with NK cell cytotoxicity in PDAC by pathway enrichment analyses (Figures 6A–6C, S6A, and S6B). In both cohorts, highly cytotoxic (ctx^{hi}) NK_C1 cells (Figures 5B–5F) were robustly enriched for several pathways, including allograft rejection, indicating intratumoral cytotoxicity, mTOR signaling, a hallmark of NK cell reactivity,[53] and IL-2 signaling, fostering NK cell cytotoxicity[54] (Figures 6B and 6C). To test whether PDAC cells directly inhibit these pathways in a TIMP-1-dependent manner, we either co-cultured NK cells with PDAC cell lines or exposed them to PDAC-cell-derived supernatants and subsequently assessed cytotoxicity, rhIL-2 responses, and mTOR activity (Figures 6D, S6C, and S6D). rhIL-2 strongly promoted NK cell killing of K562 targets, which was significantly suppressed upon PDAC cell co-culture in a TIMP-1-dependent manner (Figure 6E). Moreover, PDAC cell co-culture TIMP-1-dependently suppressed mTOR (p-Ser2448) signaling and its downstream target S6 (p-Ser235/236)[55] in NK cells (Figure 6F), which was restored by prolonged rhIL-2 exposure only for TIMP-1-deficient PDAC cells (Figure 6G). This was independent of cell-cell contact, as PDAC-cell-derived supernatants were sufficient to inhibit IL-2-mediated mTOR activity in NK cells (Figure 6H) in a TIMP-1-dependent manner, which was also evident for suppressed NK cell cytokine production (interferon gamma [IFN-γ] and tumor necrosis factor alpha [TNF-α]) (Figure 6I) and anabolic pathways (cell growth and neutral lipid content) (Figures 6J and 6K) downstream of IL-2/mTOR activation. Consistently, rhTIMP-1 rapidly and dose-dependently reduced phospho-mTOR and phospho-S6 levels in NK cells (Figures 6L and 6M) while increasing STAT3 phosphorylation (p-Tyr705) (Figure S6E), a known downstream target of TIMP-1/CD74 signaling[52] also involved in inhibiting NK cell cytotoxicity.[56] Taken together, these findings demonstrated that PDAC cells depend on TIMP-1 to modulate critical signaling pathways in NK cells, ultimately suppressing their cytotoxic capacity.

Addressing the clinical impact of the TIMP-1/NK suppression axis in PC patients (TCGA-PAAD), we determined patient-specific risk for metastasis-free and general recurrence-free survival (MFS and RFS) based on *TIMP1* expression and CIBERSORTx-derived NK cell activity as risk parameters. This revealed a highly significant inverse correlation between both risk factors, stratifying patients into four distinct risk

Figure 3. snRNA-seq identifies epithelial-subtype-specific CISS upregulation toward TIMP-1^{hi} basal-like PDAC

(A) UMAP embedding of PDAC patient tumor ($n = 17$) snRNA-seq and post-hoc cell-type annotation. CAF, cancer-associated fibroblasts; VSMC, vascular smooth muscle cells.

(B) Selected marker genes (Z scores) and proportion of positive cells in indicated cell types.

(C) Non-epithelial cell UMAP embeddings and post-hoc cell-type annotation (see Figures S3B–S3F).

(D) Epithelial cell UMAP embeddings and post-hoc cell-type annotation.

(E) Selected marker genes (Z scores) and proportion of positive cells in epithelial subsets.

(F and G) RNA velocity analysis to infer transcriptional dynamics in acinar/ductal cells (F) and cell state progression across neoplastic/malignant cells (G). Statistics (G) by Kruskal-Wallis and Dunn's test compared to PanIN.

(H) CISS expression in epithelial subsets and induction compared to acinar cells by Kruskal-Wallis and Dunn's test.

(I) (Left) CISS factor expression in epithelial subsets normalized to acinar (mean ±95% CI). (Right) Heatmap and statistics of CISS factors by Kruskal-Wallis and Dunn's test.

(J) Normalized *TIMP1* expression on the UMAP embedding.

(K) (Left) *TIMP1* prevalence within the CISS by comparing norm. CISS expression with *TIMP1* (red) or without *TIMP1* (black) (mean ±95% CI). Statistics by Mann-Whitney tests. (Right) Volcano plot of *TIMP1* prevalence across epithelial clusters identified TIMP1^{lo} classical, TIMP1^{int} basal-like, and TIMP1^{hi} basal-like cancer cells. Groups identified by log2 fold-changes and significance of *TIMP1* prevalences within CISS pattern (left).

(L) *TIMP1* expression and proportion of individual cell subtypes within epithelial compartment across patient tumors. For all statistics: n.s., non-significant; $^{*}p < 0.05$, $^{**}p < 0.01$; $^{***}p < 0.001$; $^{****}p < 0.0001$.

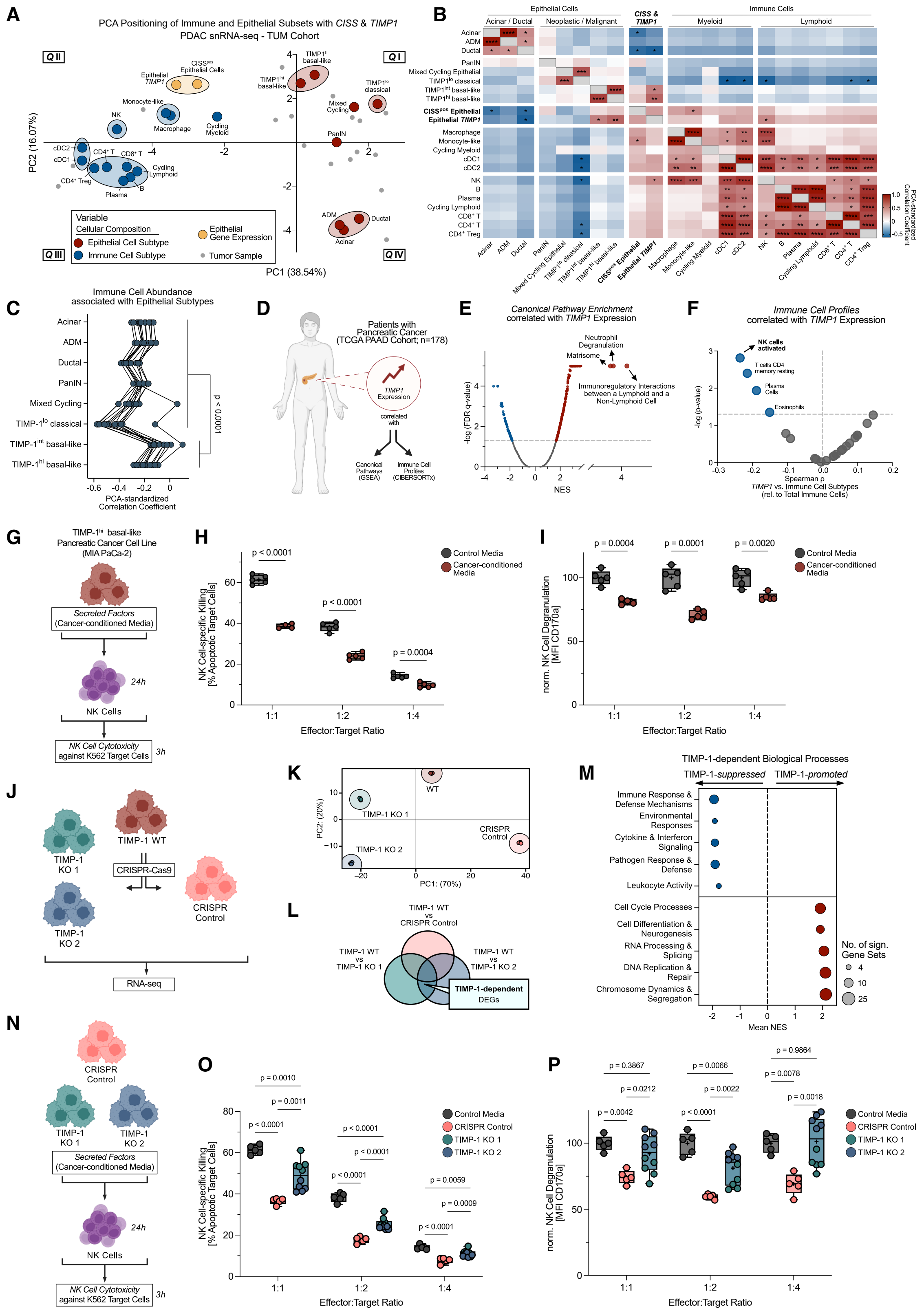

(legend on next page)

groups based on individual hazard ratios (low, moderate low, moderate high, and high) (Figures 6N and 6O). Notably, combining *TIMP1* expression and NK cell activity yielded stronger prognostic power for both MFS and RFS than each risk factor individually, which was further surpassed in significance by evaluating the complete CISS profile (Figure 6P). This demonstrated a close link between *TIMP1*/CISS upregulation and NK suppression in PDAC, directly reflected in patient-specific clinical risk.

Multikinase inhibition targets TIMP-1 and CISS and enhances NK cell cytotoxicity in TIMP-1^{hi}/CISS^{hi} basal-like PDAC *in vivo*

Toward a clinically exploitable strategy to target TIMP-1/CISS, we investigated upstream regulators of their expression in our PDAC snRNA-seq dataset (Figures 7A–7C). *TIMP1* expression and the TIMP-1^{hi}/CISS^{hi} basal-like subtype (Figure 7A) were most strongly correlated with a kinase activity pattern including MEK/ERK and receptor tyrosine kinases (RTK), such as fibroblast growth factor receptors (FGFRs) (Figures 7B and 7C). Employing the two clinically approved inhibitors trametinib (MEK) and nintedanib (multi-RTK, including FGFR), we observed a robust and, importantly, synergistic suppression of TIMP-1 expression in human PDAC cells (Figures 7D and 7E). Having previously shown that combined trametinib/nintedanib treatment re-sensitizes basal-like PDAC to ICB with α-PDL1 therapy,[43] this now prompted us to assess its TIMP-1/CISS-inhibitory effect and impact on NK cell activity in orthotopic transplantation models of classical and basal-like PDAC (Figure 7F). scRNA-seq analysis (Figure 7G) confirmed that these models closely recapitulate TIMP-1^{lo}/CISS^{lo} classical and TIMP-1^{hi}/CISS^{hi} basal-like PDAC (Figures 7H–7J) as seen in patients (Figures 3H–3L and 7A), with *Timp1* as the most basal-like PDAC-specific CISS factor (Figure 7J). Importantly, combined kinase inhibition suppressed multiple CISS factors, including *Timp1*, in a basal-like PDAC-specific manner *in vivo* (Figure 7K), which was directly correlated with increased survival, tumor regression, and response to ICB[43] (Figures S7D and S7E). Finally, intratumoral NK cells enriched upon TIMP-1/CISS-targeting therapy (Figures 7L–7O), particularly in basal-like PDAC and when combined with ICB (Figure 7M), showed enhanced IL-2 and mTOR-pathway activity and increased cytotoxicity genes (Figures 7N and 7O), paralleling *ctx*^{hi} NK cells (Figures 5B–5F and 6A–6C) and the TIMP-1-dependent suppression observed in human PDAC. Together, these findings proved pharmacological inhibition of upstream kinase pathways as effective strategy to target TIMP-1/CISS, supporting its translational potential to restrain PDAC progression and enhance NK cell activity.

DISCUSSION

During malignant progression, cancer cells acquire molecular mechanisms to orchestrate a growth-permissive environment and evade immune control.[4–6] Refining our understanding of cancer-borne immunosuppression has widely improved diagnostic and therapeutic options, including patient-tailored immunotherapies.[57] However, many highly lethal cancers, such as PDAC, remain refractory to these clinical advances,[9,58] reflecting an incomplete understanding of overarching principles of cancer-borne immunosuppression. Here, we elucidated a tumor-progression-dependent CISS, whose presence robustly correlated with poor survival and immunosuppressive TMEs across multiple human cancers. Moreover, we identified CISS and its dominant factor TIMP-1 as therapeutically targetable using clinically approved kinase inhibitors in PDAC.

Our findings contribute to closing an essential gap in understanding immunosuppression as an early and constantly evolving capacity of pancreatic epithelial cells, enabling them to regulate their immune microenvironment at each phase of transformation and malignant progression.[14,21,25] Intriguingly, while CISS already arose in pre-malignant transitions and closely resembled early epithelial inflammation, its elevation was most pronounced in most progressed basal-like PDAC. This corroborates the hypothesis that cancer cells orchestrate immunosuppression by co-opting hard-wired gene expression programs from normal cells[7] and reinforces the crucial role of inflammation for PDAC progression.[26–29] In fact, CISS comprised various known tumor-derived

Figure 4. CISS-prevalent TIMP-1 is causal for PDAC-cell-induced NK cell suppression

(A) PCA integrating epithelial (red) and immune (blue) cell fractions, fractions of CISS-expressing epithelial cells, and epithelial *TIMP1*.

(B) Pearson correlation between PCA-standardized variables (A) (*$p < 0.05$, **$p < 0.01$; ***$p < 0.001$; ****$p < 0.0001$).

(C) Comparison of correlations between epithelial and immune subtypes (A and B) by paired Student's t test between indicated groups.

(D–F) Workflow (D) to identify canonical pathways [GSEA; (E)] and immune cell profiles [CIBERSORTx; (F)] correlated with *TIMP1* expression (TCGA-PAAD). For GSEA, genes were ranked by correlation coefficients (Spearman) with *TIMP1* expression. Reference gene set: C2:CP.

(G–I) Workflow (G) for MIA PaCa-2-cell-derived secreted factors suppressing NK cell killing of K562 targets (H) and degranulation (I) at indicated effector-to-target ratios (E:T). Comparison to control media [no cancer conditioning; shared with (O,P)] by unpaired Student's t tests.

(J) Workflow for CRISPR-Cas9-based TIMP-1 knockout in MIA PaCa-2 cells and RNA-seq.

(K) PCA of RNA-seq data [$n = 3$ per cell line; (J)].

(L) TIMP-1-dependent DEGs (DESeq2) in MIA PaCa-2 cells. Intersection shows DEGs between TIMP-1 WT and TIMP-1 KO (1 and 2) cells, independent of CRISPR-Cas9 (CRISPR Control).

(M) GSEA of TIMP-1-dependent biological processes in MIA PaCa-2 cells. log2 fold-changes of DEGs (L) between means of TIMP-1-competent ("TIMP-1 WT"/"CRISPR Control") cells and TIMP-1-deficient ("TIMP-1 KO 1/2") cells. Reference gene set: GO:BP. Enriched gene sets (FDR q < 0.05) were categorized (see Figure S4G).

(N–P) Workflow (N) of TIMP-1-dependent NK cell suppression of CRISPR-Cas9-derived MIA PaCa-2 cell lines (J) on K562 target cell killing (O) and degranulation (P) at indicated E:T. Statistics by one-way ANOVA and Tukey test for indicated groups. Data in (H, I, K, O, and P) showing biological replicates as box and whiskers plots (H, I, O, and P). (D, G, J, and N) Created with BioRender.com.

CellPress
OPEN ACCESS

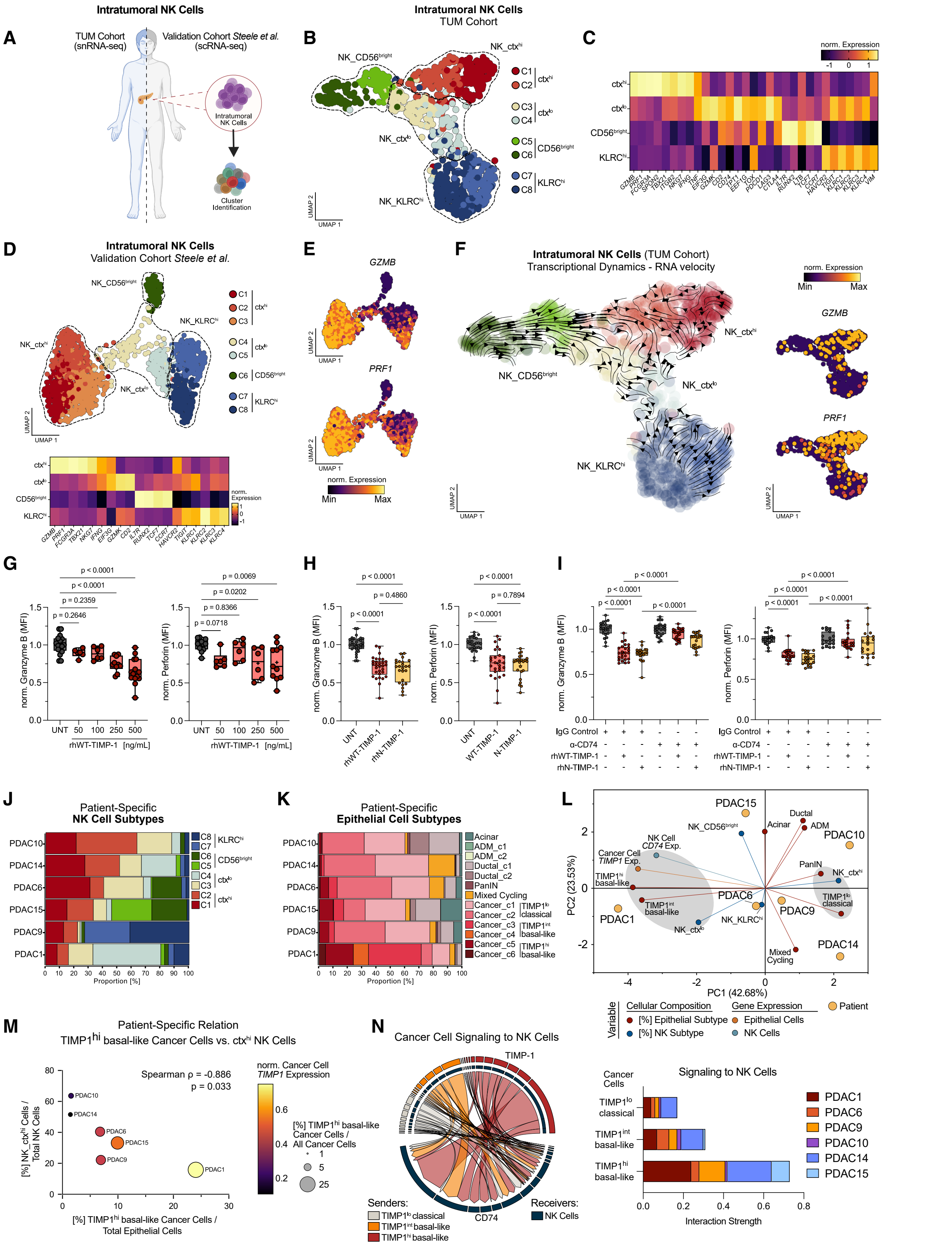

(legend on next page)

secreted factors with pro-tumor immunoregulatory activity, such as TIMP-1,[39,48,59] TGFB1,[60] SPP1,[61] or VCAN,[62] for which we revealed dynamic, context-dependent expression patterns during PDAC progression.

While most tumors in our patient cohort were enriched for the classical PDAC subtype, we could pinpoint particularly strong immunoregulatory potential for basal-like PDAC cells, even if present in lower numbers, supporting previous reports that highlighted their particular aggressiveness.[21,25,43] We elucidated TIMP-1 as an important contributor to this phenomenon and as a robust determinant of progression toward basal-like cancer in patients. Consistently, TIMP-1 has been previously described as a highly abundant circulating biomarker for basal-like PDAC in pre-clinical models.[63] Our study advances these findings on a functional and clinical level, substantiating TIMP-1 as a robust marker of PDAC progression, diagnostically detectable through liquid biospies,[48,64] and a PDAC-subtype-specific therapeutic target. Thus, our findings on TIMP-1^{hi}/CISS^{hi} high-risk patients may aid in stratifying those most likely to benefit from TIMP-1/CISS-targeting therapy combined with immunotherapy, in PDAC as well as in other aggressive malignancies such as TIMP-1^{hi} triple-negative breast cancer[65] or brain metastasis.[66]

Functionally, we discovered non-canonical TIMP-1/CD74 signaling as a mechanism of NK cell suppression, reinforcing previous reports on CD74 as a receptor for immunosuppressive signals to NK cells within the TME.[67–69] Importantly, we could pinpoint TIMP-1 as necessary and sufficient for suppressing mTOR signaling and cytotoxic effectors in NK cells. This substantiates a crucial role for TIMP-1 signaling in pro-tumor immune regulation in line with previously described effects on CD8^{+} T cells[66,70] and myeloid cells, such as neutrophils[59] and monocytes.[71]

In perspective, although NK cells emerge as promising cytotoxic effectors in cancer therapy, their implementation against solid tumors remains challenging,[32] particularly in poorly NK-cell-infiltrated cancers such as PDAC.[72,73] While studies of NK cell function in PDAC are limited, recent evidence showed that active NK cells interact with malignant epithelium in human PDAC[74] and are essential for anti-tumor immunity in preclinical PDAC,[75] potentially also through crosstalk with other immune cells to coordinate multicellular immune responses.[32,33] Thus, by delineating intratumoral NK cell subsets in PDAC patients and highlighting their responsiveness to TIMP-1/CISS-targeting therapy, our study may promote further investigation into their functional significance and therapeutic potential in cancer. Collectively, we elucidate CISS as a molecular signature of pro-tumor immune regulation across human cancers, which offers a broad and exploitable rationale for future studies.

Limitations of the study

Our study establishes a strong association between TIMP-1/CISS and impaired NK cell activity across cancers. However, CISS-associated immune regulation is likely not limited to TIMP-1-dependent NK cell suppression and may involve various immune cells and their concerted interplay for anti-tumor immunity. This remains to be established in future studies, e.g., built on context-specific upregulation of the diverse pro-tumor CISS factors[39,48,59–62] described here. Moreover, the functional role of individual CISS factors at different phases of premalignancy and cancer progression remains to be defined. While we observed CISS induction in epithelial inflammation and pre-malignant transitions, we primarily focused on advanced TIMP-1^{hi}/CISS^{hi} basal-like PDAC; whether targeting CISS can intercept cancer at a pre-malignant state remains to be tested. Further, it is important to note that immunosuppression is also shaped by stromal TME cells, such as cancer-associated fibroblasts,[2] whose contribution to TIMP-1/CISS activity warrants further investigation. Finally, it should be noted that, for constructing risk scores for RFS and MFS (STAR Methods), patients who died without documented recurrence were censored at the time of death, since the cause of death in such cases was not uniformly annotated.

RESOURCE AVAILABILITY

Lead contact

Requests for further information and resources should be directed to and will be fulfilled by the lead contact, Prof. Dr. Achim Krüger (achim.krueger@tum.de).

Materials availability

All unique/stable materials generated in this study are available from the lead contact.

Figure 5. CISS-prevalent TIMP-1 is sufficient to suppress cytotoxic capacity in NK cells via CD74 signaling

(A–F) Workflow (A) to identify NK cell clusters in TUM Cohort (B, C, and F) and Steele Cohort[22] (D and E). (B and D) NK cell UMAP embeddings, post-hoc annotations, and selected marker genes (*Z* scores) (C and D, bottom). ctx, cytotoxicity. KLRC, killer cell lectin like receptor C gene family. (E and F, right) *GZMB* and *PRF1* expression on the UMAP (B and D). (F, left) RNA velocity to infer transcriptional dynamics in NK cell clusters.

(G–I) NK cell granzyme B and perforin levels upon exposure to (G) rhWT-TIMP-1 (granzyme B: UNT, $n = 17$; 50 ng/mL, $n = 6$; 100 ng/mL, $n = 6$; 250 ng/mL, $n = 10$; 500 ng/mL, $n = 14$; perforin: UNT, $n = 12$; 50 ng/mL, $n = 6$; 100 ng/mL, $n = 6$; 250 ng/mL, $n = 6$; 500 ng/mL, $n = 11$). (H) 500 ng/mL WT-TIMP-1 vs. equimolar N-TIMP-1 (granzyme B: UNT, $n = 32$; WT-TIMP-1, $n = 30$; N-TIMP-1, $n = 24$; perforin: UNT, $n = 27$; WT-TIMP-1, $n = 27$; N-TIMP-1, $n = 24$); (I) 500 ng/mL WT-TIMP-1 vs. equimolar N-TIMP-1 with or without α-CD74 antibody milatuzumab or immunoglobulin G (IgG) control (granzyme B: IgG alone, $n = 23$; IgG + WT-TIMP-1, $n = 25$; IgG + N-TIMP-1, $n = 20$; α-CD74 alone, $n = 26$; α-CD74 + WT-TIMP-1, $n = 24$; α-CD74 + N-TIMP-1, $n = 20$; perforin: IgG alone, $n = 19$; IgG + WT-TIMP-1, $n = 20$; IgG + N-TIMP-1, $n = 20$; α-CD74 alone, $n = 19$; α-CD74 + WT-TIMP-1, $n = 20$; α-CD74 + N-TIMP-1, $n = 20$). Data (G–I) pooled from four independent experiments show biological replicates (box and whiskers plots) derived from six healthy donors. Statistics by one-way ANOVA and Dunnett test (G) or one-way ANOVA and Tukey test (H and I) across indicated groups.

(J and K) Proportions of NK (J) and epithelial (K) subsets within samples (TUM Cohort).

(L) PCA of NK and epithelial subsets (J and K), NK cell *CD74*, and cancer cell *TIMP1* expression (small dots) across patients (large dots). Exp., expression. (M) Spearman correlation between TIMP1^{hi} basal-like cancer cells and ctx^{hi} NK cells. (N) Inferred cancer cell-to-NK cell signaling by CellChat in tumors (J and K) (also see Figure S5H). (A) Created with BioRender.com.

CellPress
OPEN ACCESS

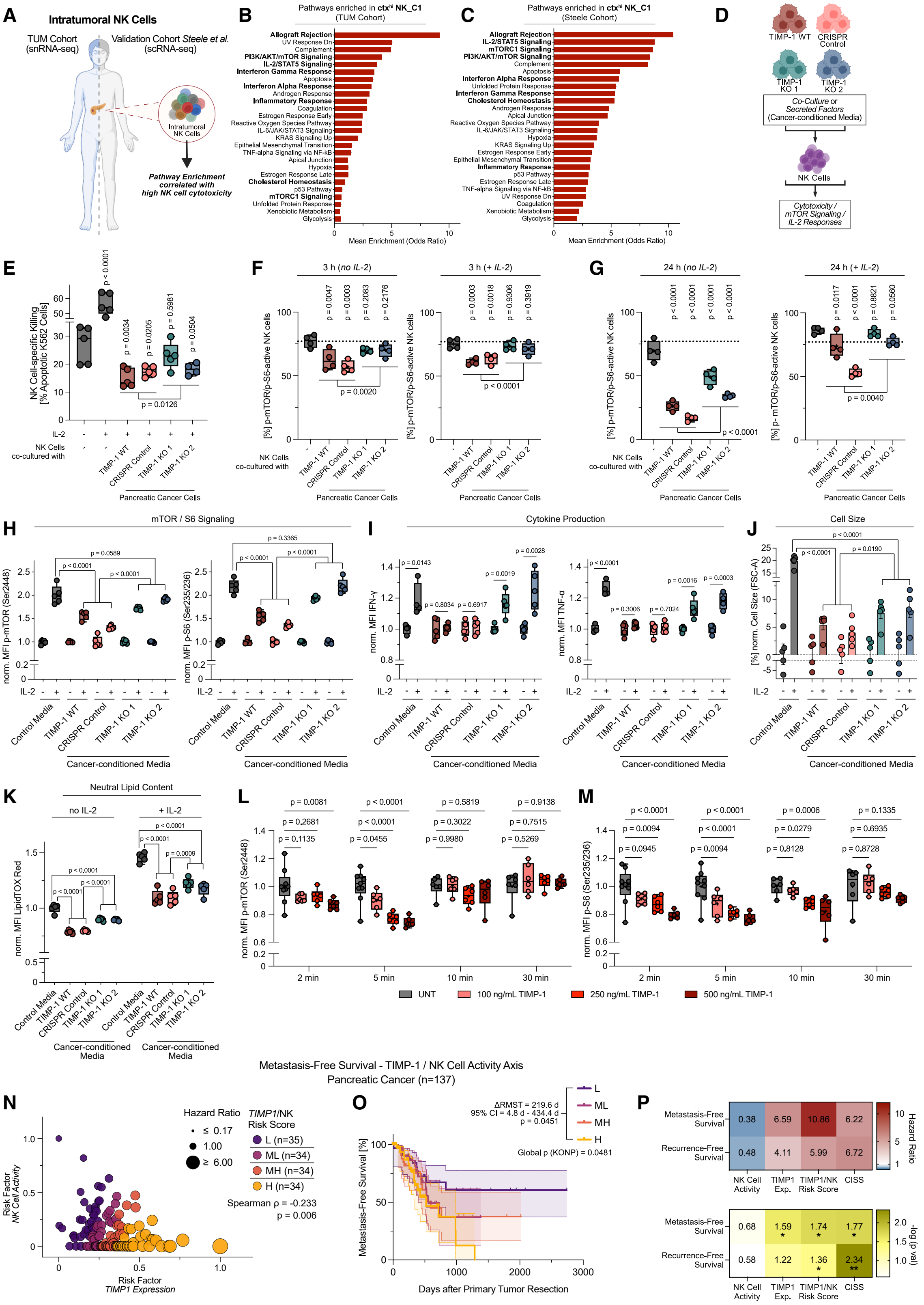

(legend on next page)

Data and code availability

- Bulk RNA-seq data from pancreatic tissues (GSE290898) and cell lines (GSE290413) are deposited in the Gene Expression Omnibus (GEO) database. Raw (PRJNA1230557) and processed (GSE291124) deidentified snRNA-seq data are deposited in the Sequence Read Archive (SRA) and GEO, respectively. Re-analyzed publicly available datasets have been retrieved as indicated (see STAR Methods).
- This paper does not report original code. All analyses using publicly available software/codes are indicated (see STAR Methods).
- Any additional information required to reanalyze the data reported in this paper is available from the lead contact upon request.

ACKNOWLEDGMENTS

We thank Chris D. Hermann for preliminary analytical support and Annika Fröhlich and Lars-Henrik Joost for technical assistance (all Institute of Experimental Oncology and Therapy Research). This work was supported by the Deutsche Forschungsgemeinschaft, Bonn, Germany (KR2047/15-1, to A.K.), the Else Kröner Clinician Scientist Professorship for Translational Pancreatic Surgery (to I.E.D.), and the Klaus Tschira Boost Fund, a joint initiative of the German Scholars Organization and the Klaus Tschira Foundation (to S.B.).

AUTHOR CONTRIBUTIONS

Conceptualization, J.F. and A.K.; investigation, J.F., C.M.R., M.H., V.B., B.T., A.S., D.H., D.B., K.Ç., K.S., and S.B.; formal analysis, J.F., C.M.R., M.H., V.B., B.T., D.M., A.S., D.H., D.B., D.L., J.M.d.V., L.H., R.K.-R., K.S., J.P.B., and A.K.; methodology, J.F., C.M.R., V.B., B.T., A.S., D.H., D.B., D.L., J.M.V., L.H., R.K.-R., K.S., and S.B.; resources, C.M.R., A.S., D.H., D.L., J.M.d.V., L.H., R.K.-R., A.H., R.Ö., P.A.K., K.S., R.R., F.J.T., F.X.R., S.B., D.S., I.E.D., and A.K.; funding acquisition, S.B., I.E.D., and A.K.; supervision, D.S., I.E.D., and A.K.; writing, J.F. and A.K. All authors contributed to feedback and proofreading.

DECLARATION OF INTERESTS

The authors declare no competing interests.

DECLARATION OF GENERATIVE AI AND AI-ASSISTED TECHNOLOGIES IN THE WRITING PROCESS

During the preparation of the manuscript, the authors used ChatGPT in order to reduce length and increase brevity. After using this tool, the authors reviewed and edited the content as needed and take full responsibility for the content of the publication.

STAR★METHODS

Detailed methods are provided in the online version of this paper and include the following:

- KEY RESOURCES TABLE
- EXPERIMENTAL MODEL AND STUDY PARTICIPANT DETAILS
  - PDAC patients and blood donors
  - Animal models
  - Cell lines
- METHOD DETAILS
  - Steele Cohort data retrieval
  - Cao Cohort data retrieval
  - Abdollahi Cohort data retrieval
  - The Cancer Genome Atlas (TCGA) cohorts
  - Mouse RNA-seq data retrieval
  - Identification of the CISS profile
  - Survival analyses
  - Bulk RNA-seq of tissues and cell lines
  - Bulk RNA-seq data processing
  - Gene Set Enrichment Analyses
  - Immune cell type abundance using CIBERSORTx
  - Sample preparation for snRNA-seq
  - snRNA-seq library preparation and sequencing
  - snRNA-seq data preprocessing and quality control
  - Data integration, dimensionality reduction, clustering, and cell-type-specific analysis
  - RNA velocity analysis
  - Gene set scoring with UCell
  - Copy-number variations (CNVs) in epithelial cell types
  - Pathway enrichment analysis in scRNA-/snRNA-seq datasets
  - Principal component analyses (PCA) of tumor heterogeneity
  - Inference of cell-cell communication
  - Cell line transcriptome screening
  - TIMP-1-dependent functions in MIA PaCa-2 cells
  - Intracellular TIMP-1 measurement in PDAC cells
  - Primary human NK cells
  - NK cell signaling assays
  - NK cell cytotoxicity assay
- QUANTIFICATION AND STATISTICAL ANALYSIS
  - Statistical analyses

SUPPLEMENTAL INFORMATION

Supplemental information can be found online at https://doi.org/10.1016/j.xcrm.2025.102546.

Figure 6. TIMP-1-dependent suppression of NK cell mTOR signaling links PDAC immunosuppression to clinical risk profiles

(A–C) Workflow (A) to identify enriched pathways in NK_ctx^{hi} C1 cluster vs. all other NK clusters in TUM Cohort (B) and Steele Cohort (C). DEGs (adj. $p < 0.05$) by Wilcoxon rank-sum test and auROC analysis. Mean odds ratios by pathway enrichment using Enrichr (Hallmark reference gene sets; see Figures S6A and S6B, STAR Methods). AKT, protein kinase B; IL-2, interleukin-2; mTORC1, mechanistic target of rapamycin complex 1; PI3K, phosphoinositide 3-kinase; STAT5, signal transducer and activator of transcription 5; UV, ultraviolet.

(D–K) Workflow (D) to identify TIMP-1-dependent MIA PaCa-2-cell-mediated suppression of mTOR-signaling and IL-2 responses in NK cells, using co-culture [3 h (F); 24h (E and G)] or cancer-cell-conditioned media [24 h (H and I); 72 h (J and K)]. NK cell suppression with or without IL-2 activation assessed by (E) K562 killing (E:T 1:3; killing for 3 h); (F–H) p-mTOR(Ser2448)/p-S6(Ser235/236) signaling; (I) intracellular NK cell IFN-γ and TNF-α; (J) cell growth; (K) neutral lipid content; norm. to IL-2-free controls (H–J). Statistics between indicated groups: one-way ANOVA and Dunnett test (E–G, upper), one-way ANOVA and Tukey test (H, J, and K), unpaired Student's *t* tests (E–G, lower; I).

(L and M) NK cell (L) p-mTOR and (M) p-S6 levels upon exposure to rhWT-TIMP-1. Statistics by two-way ANOVA and Dunnett test. Data (E–M) shown as biological replicates [box and whiskers plots (E–I, and K–M) or mean ± SEM (J)].

(N–P) Cox-regression-based *TIMP1*/NK risk score for recurrence-free (RFS) or metastasis-free survival (MFS) (TCGA-PAAD, $n = 137$), (N) For MFS, patient hazard ratios (HRs), and risk groups separated by quartiles. Statistics between linear predictors of *TIMP1* expression and NK cell activity by Spearman correlation. (O) For MFS, survival probabilities by Kaplan-Meier curves (±95% CI). Statistics: global differences by KONP test, restricted mean survival time (RMST; $\tau = 0.9$) between high (H) and low (L) risk groups by Wald test. (P) Heatmaps showing HRs (upper) and significance (lower) for the *TIMP1*/NK score, both factors individually, and CISS by Cox regression analyses and G squared log likelihood ratio (*$p < 0.05$; **$p < 0.01$). (A and D) Created with BioRender.com.

CellPress
OPEN ACCESS

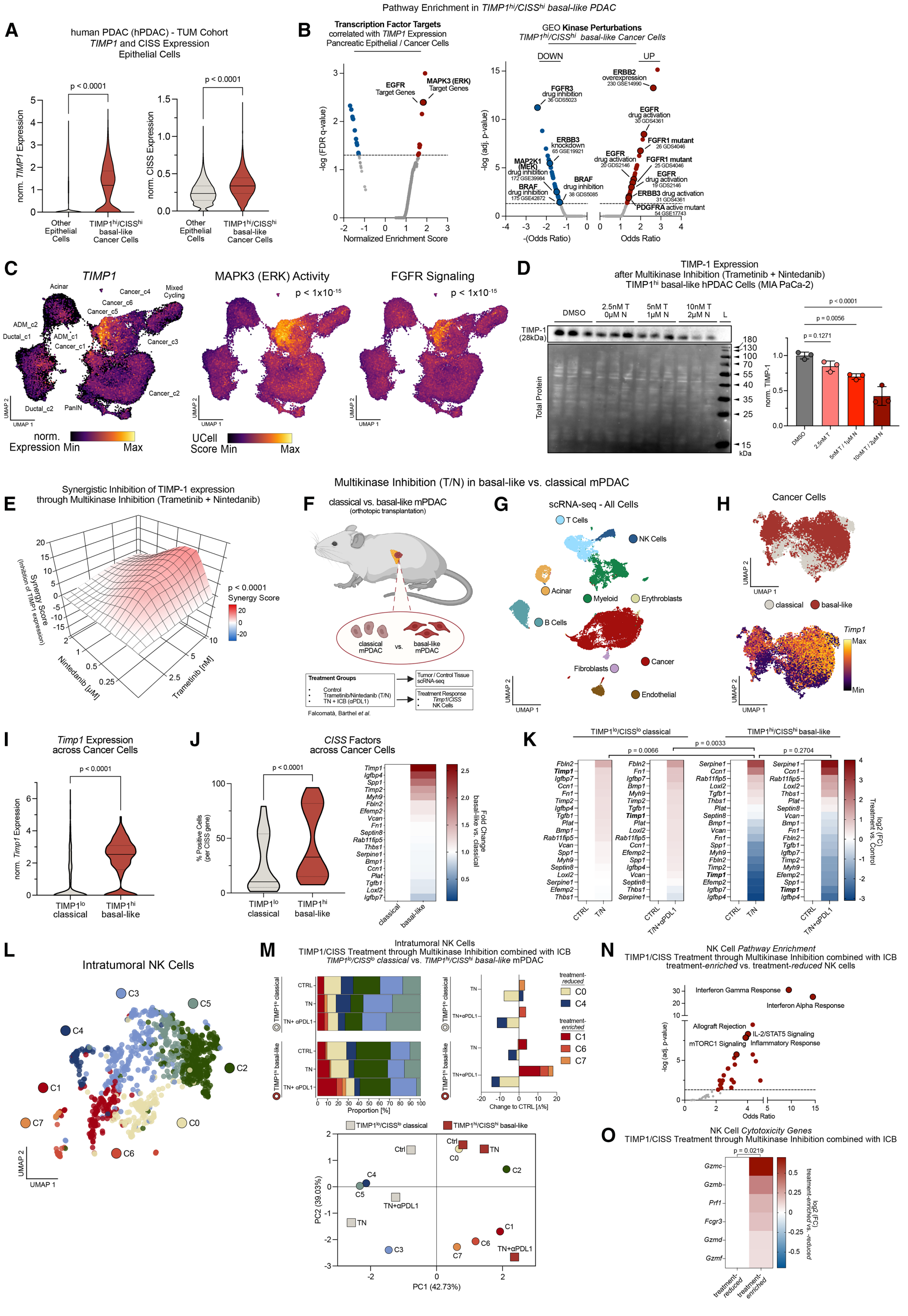

(legend on next page)

Received: April 30, 2025
Revised: September 24, 2025
Accepted: December 9, 2025
Published: January 20, 2026

REFERENCES

1. Garner, H., and de Visser, K.E. (2020). Immune crosstalk in cancer progression and metastatic spread: a complex conversation. Nat. Rev. Immunol. *20*, 483–497. https://doi.org/10.1038/s41577-019-0271-z.
2. Hingorani, S.R. (2023). Epithelial and stromal co-evolution and complicity in pancreatic cancer. Nat. Rev. Cancer *23*, 57–77. https://doi.org/10.1038/s41568-022-00530-w.
3. de Visser, K.E., and Joyce, J.A. (2023). The evolving tumor microenvironment: From cancer initiation to metastatic outgrowth. Cancer Cell *41*, 374–403. https://doi.org/10.1016/j.ccell.2023.02.016.
4. Galassi, C., Chan, T.A., Vitale, I., and Galluzzi, L. (2024). The hallmarks of cancer immune evasion. Cancer Cell *42*, 1825–1863. https://doi.org/10.1016/j.ccell.2024.09.010.
5. Hanahan, D., and Weinberg, R.A. (2011). Hallmarks of cancer: the next generation. Cell *144*, 646–674. https://doi.org/10.1016/j.cell.2011.02.013.
6. van Weverwijk, A., and de Visser, K.E. (2023). Mechanisms driving the immunoregulatory function of cancer cells. Nat. Rev. Cancer *23*, 193–215. https://doi.org/10.1038/s41568-022-00544-4.
7. Hanahan, D., Michielin, O., and Pittet, M.J. (2025). Convergent inducers and effectors of T cell paralysis in the tumour microenvironment. Nat. Rev. Cancer *25*, 41–58. https://doi.org/10.1038/s41568-024-00761-z.
8. Sharma, P., Goswami, S., Raychaudhuri, D., Siddiqui, B.A., Singh, P., Nagarajan, A., Liu, J., Subudhi, S.K., Poon, C., Gant, K.L., et al. (2023). Immune checkpoint therapy-current perspectives and future directions. Cell *186*, 1652–1669. https://doi.org/10.1016/j.cell.2023.03.006.
9. Morrison, A.H., Byrne, K.T., and Vonderheide, R.H. (2018). Immunotherapy and Prevention of Pancreatic Cancer. Trends Cancer *4*, 418–428. https://doi.org/10.1016/j.trecan.2018.04.001.
10. Hidalgo, M., Cascinu, S., Kleeff, J., Labianca, R., Löhr, J.M., Neoptolemos, J., Real, F.X., Van Laethem, J.L., and Heinemann, V. (2015). Addressing the challenges of pancreatic cancer: future directions for improving outcomes. Pancreatology *15*, 8–18. https://doi.org/10.1016/j.pan.2014.10.001.
11. Halbrook, C.J., Lyssiotis, C.A., Pasca di Magliano, M., and Maitra, A. (2023). Pancreatic cancer: Advances and challenges. Cell *186*, 1729–1754. https://doi.org/10.1016/j.cell.2023.02.014.
12. Karamitopoulou, E. (2019). Tumour microenvironment of pancreatic cancer: immune landscape is dictated by molecular and histopathological features. Br. J. Cancer *121*, 5–14. https://doi.org/10.1038/s41416-019-0479-5.
13. Ho, W.J., Jaffee, E.M., and Zheng, L. (2020). The tumour microenvironment in pancreatic cancer - clinical challenges and opportunities. Nat. Rev. Clin. Oncol. *17*, 527–540. https://doi.org/10.1038/s41571-020-0363-5.
14. Falcomatà, C., Bärthel, S., Schneider, G., Rad, R., Schmidt-Supprian, M., and Saur, D. (2023). Context-Specific Determinants of the Immunosuppressive Tumor Microenvironment in Pancreatic Cancer. Cancer Discov. *13*, 278–297. https://doi.org/10.1158/2159-8290.CD-22-0876.
15. Clark, C.E., Hingorani, S.R., Mick, R., Combs, C., Tuveson, D.A., and Vonderheide, R.H. (2007). Dynamics of the immune reaction to pancreatic cancer from inception to invasion. Cancer Res. *67*, 9518–9527. https://doi.org/10.1158/0008-5472.CAN-07-0175.
16. Ying, H., Dey, P., Yao, W., Kimmelman, A.C., Draetta, G.F., Maitra, A., and DePinho, R.A. (2016). Genetics and biology of pancreatic ductal adenocarcinoma. Genes Dev. *30*, 355–385. https://doi.org/10.1101/gad.275776.115.
17. Siegel, R.L., Kratzer, T.B., Giaquinto, A.N., Sung, H., and Jemal, A. (2025). Cancer statistics, 2025. CA Cancer J. Clin. *75*, 10–45. https://doi.org/10.3322/caac.21871.
18. Collisson, E.A., Sadanandam, A., Olson, P., Gibb, W.J., Truitt, M., Gu, S., Cooc, J., Weinkle, J., Kim, G.E., Jakkula, L., et al. (2011). Subtypes of pancreatic ductal adenocarcinoma and their differing responses to therapy. Nat. Med. *17*, 500–503. https://doi.org/10.1038/nm.2344.
19. Moffitt, R.A., Marayati, R., Flate, E.L., Volmar, K.E., Loeza, S.G.H., Hoadley, K.A., Rashid, N.U., Williams, L.A., Eaton, S.C., Chung, A.H., et al. (2015). Virtual microdissection identifies distinct tumor- and stroma-specific subtypes of pancreatic ductal adenocarcinoma. Nat. Genet. *47*, 1168–1178. https://doi.org/10.1038/ng.3398.
20. Bailey, P., Chang, D.K., Nones, K., Johns, A.L., Patch, A.M., Gingras, M.C., Miller, D.K., Christ, A.N., Bruxner, T.J.C., Quinn, M.C., et al. (2016). Genomic analyses identify molecular subtypes of pancreatic cancer. Nature *531*, 47–52. https://doi.org/10.1038/nature16965.
21. Connor, A.A., and Gallinger, S. (2022). Pancreatic cancer evolution and heterogeneity: integrating omics and clinical data. Nat. Rev. Cancer *22*, 131–142. https://doi.org/10.1038/s41568-021-00418-1.
22. Steele, N.G., Carpenter, E.S., Kemp, S.B., Sirihorachai, V.R., The, S., Delrosario, L., Lazarus, J., Amir, E.A.D., Gunchick, V., Espinoza, C., et al. (2020). Multimodal Mapping of the Tumor and Peripheral Blood

Figure 7. Multikinase inhibition targets TIMP-1 and CISS and enhances NK cell cytotoxicity in TIMP1^{hi}/CISS^{hi} basal-like PDAC *in vivo*

(A) TIMP1^{hi}/CISS^{hi} basal-like PDAC in patients based on TIMP1/CISS expression (see Figure 3). Statistics by Mann-Whitney tests.
(B) Transcription factor targets (TFTs) and kinase perturbations correlated with TIMP1/CISS in basal-like PDAC. (Left) Genes correlating (Spearman; $p < 0.05$) with *TIMP1* expression were ranked by coefficients for GSEA (reference: C3:TFT). DEGs (Wilcoxon rank-sum test and auROC analysis) between TIMP1^{hi}/CISS^{hi} basal-like cancer and other epithelial cells tested for pathway enrichment by Enrichr ("Kinase perturbations from GEO UP" and "DOWN").
(C) UMAP embedding of *TIMP1* expression (see Figure 3J), ERK activity, and FGFR signaling. ERK activity (TFT:MAPK3_Target_Genes) and FGFR signaling (Reactome_Signaling_by_FGFR) calculated by UCell and correlated to *TIMP1* expression (Spearman).
(D) Western blot ($n = 3$; biological replicates) of intracellular TIMP-1 in MIA PaCa-2 cells upon treatment with trametinib (T) and nintedanib (N). Statistics by one-way ANOVA and Dunnett test.
(E) ZIP synergy map of TIMP-1 inhibition (intracellular TIMP-1) in MIA PaCa-2 cells using trametinib or nintedanib (see Figure S7A).
(F) Workflow to assess *in vivo* effect of trametinib, nintedanib, and *a*-PDL1 treatment on TIMP1/CISS and NK cells in orthotopic classical and basal-like PDAC transplantation mouse models.[43]
(G–J) UMAP embedding of all cells (G) or cancer cells (H) from scRNA-seq of PDAC tumors derived from (F) and post-hoc cell-type annotations (G), cancer cell type (H, upper), or *Timp1* expression (H, lower). (I) *Timp1* and (J) CISS expression in cancer cells. Statistics by Mann-Whitney tests.
(K) CISS factors in PDAC cells upon indicated treatments vs. controls from snRNA-seq data (F–J). Changes in CISS factors calculated by pseudobulk limma-voom workflow. Statistics: one-way ANOVA for matched data (genes) and Dunnett test between indicated groups.
(L and M) UMAP embedding of NK cell clusters (M) and proportions across treatments (F,G), assessed by frequencies (M, upper) and PCA (M, lower).
(N and O) Pathways enriched (N; Enrichr using Hallmark reference gene set) and cytotoxicity gene expression (O) between treatment-*enriched* and -*reduced* NK clusters. Changes in cytotoxicity genes (O) calculated by Wilcoxon rank-sum test and auROC analysis. Statistics by paired Student's *t* test. (F) Created with BioRender.com.

Immune Landscape in Human Pancreatic Cancer. Nat. Cancer *1*, 1097–1112. https://doi.org/10.1038/s43018-020-00121-4.

23. Hwang, W.L., Jagadeesh, K.A., Guo, J.A., Hoffman, H.I., Yadollahpour, P., Reeves, J.W., Mohan, R., Drokhlyansky, E., Van Wittenberghe, N., Ashenberg, O., et al. (2022). Single-nucleus and spatial transcriptome profiling of pancreatic cancer identifies multicellular dynamics associated with neoadjuvant treatment. Nat. Genet. *54*, 1178–1191. https://doi.org/10.1038/s41588-022-01134-8.
24. Cui Zhou, D., Jayasinghe, R.G., Chen, S., Herndon, J.M., Iglesia, M.D., Navale, P., Wendl, M.C., Caravan, W., Sato, K., Storrs, E., et al. (2022). Spatially restricted drivers and transitional cell populations cooperate with the microenvironment in untreated and chemo-resistant pancreatic cancer. Nat. Genet. *54*, 1390–1405. https://doi.org/10.1038/s41588-022-01157-1.
25. Chan-Seng-Yue, M., Kim, J.C., Wilson, G.W., Ng, K., Figueroa, E.F., O'Kane, G.M., Connor, A.A., Denroche, R.E., Grant, R.C., McLeod, J., et al. (2020). Transcription phenotypes of pancreatic cancer are driven by genomic events during tumor evolution. Nat. Genet. *52*, 231–240. https://doi.org/10.1038/s41588-019-0566-9.
26. Alonso-Curbelo, D., Ho, Y.J., Burdziak, C., Maag, J.L.V., Morris, J.P., 4th, Chandwani, R., Chen, H.A., Tsanov, K.M., Barriga, F.M., Luan, W., et al. (2021). A gene-environment-induced epigenetic program initiates tumorigenesis. Nature *590*, 642–648. https://doi.org/10.1038/s41586-020-03147-x.
27. Del Poggetto, E., Ho, I.L., Balestrieri, C., Yen, E.Y., Zhang, S., Citron, F., Shah, R., Corti, D., Diaferia, G.R., Li, C.Y., et al. (2021). Epithelial memory of inflammation limits tissue damage while promoting pancreatic tumorigenesis. Science *373*, eabj0486. https://doi.org/10.1126/science.abj0486.
28. Yuan, S., Almagro, J., and Fuchs, E. (2024). Beyond genetics: driving cancer with the tumour microenvironment behind the wheel. Nat. Rev. Cancer *24*, 274–286. https://doi.org/10.1038/s41568-023-00660-9.
29. Stone, M.L., and Beatty, G.L. (2019). Cellular determinants and therapeutic implications of inflammation in pancreatic cancer. Pharmacol. Ther. *201*, 202–213. https://doi.org/10.1016/j.pharmthera.2019.05.012.
30. Hingorani, S.R., Wang, L., Multani, A.S., Combs, C., Deramaudt, T.B., Hruban, R.H., Rustgi, A.K., Chang, S., and Tuveson, D.A. (2005). Trp53R172H and KrasG12D cooperate to promote chromosomal instability and widely metastatic pancreatic ductal adenocarcinoma in mice. Cancer Cell *7*, 469–483. https://doi.org/10.1016/j.ccr.2005.04.023.
31. Cao, L., Huang, C., Cui Zhou, D., Hu, Y., Lih, T.M., Savage, S.R., Krug, K., Clark, D.J., Schnaubelt, M., Chen, L., et al. (2021). Proteogenomic characterization of pancreatic ductal adenocarcinoma. Cell *184*, 5031–5052.e26. https://doi.org/10.1016/j.cell.2021.08.023.
32. Vivier, E., Rebuffet, L., Narni-Mancinelli, E., Cornen, S., Igarashi, R.Y., and Fantin, V.R. (2024). Natural killer cell therapies. Nature *626*, 727–736. https://doi.org/10.1038/s41586-023-06945-1.
33. Coënon, L., Geindreau, M., Ghiringhelli, F., Villalba, M., and Bruchard, M. (2024). Natural Killer cells at the frontline in the fight against cancer. Cell Death Dis. *15*, 614. https://doi.org/10.1038/s41419-024-06976-0.
34. Mantovani, A., Allavena, P., Marchesi, F., and Garlanda, C. (2022). Macrophages as tools and targets in cancer therapy. Nat. Rev. Drug Discov. *21*, 799–820. https://doi.org/10.1038/s41573-022-00520-5.
35. Shaul, M.E., and Fridlender, Z.G. (2019). Tumour-associated neutrophils in patients with cancer. Nat. Rev. Clin. Oncol. *16*, 601–620. https://doi.org/10.1038/s41571-019-0222-4.
36. Schlesinger, Y., Yosefov-Levi, O., Kolodkin-Gal, D., Granit, R.Z., Peters, L., Kalifa, R., Xia, L., Nasereddin, A., Shiff, I., Amran, O., et al. (2020). Single-cell transcriptomes of pancreatic preinvasive lesions and cancer reveal acinar metaplastic cells' heterogeneity. Nat. Commun. *11*, 4516. https://doi.org/10.1038/s41467-020-18207-z.
37. Peng, J., Sun, B.F., Chen, C.Y., Zhou, J.Y., Chen, Y.S., Chen, H., Liu, L., Huang, D., Jiang, J., Cui, G.S., et al. (2019). Single-cell RNA-seq highlights intra-tumoral heterogeneity and malignant progression in pancreatic ductal adenocarcinoma. Cell Res. *29*, 725–738. https://doi.org/10.1038/s41422-019-0195-y.
38. Yamamoto, N., Dobersch, S., Loveless, I., Samraj, A.N., Jang, G.H., Haraguchi, M., Kang, L.I., Ruzinova, M.B., Vij, K.R., Mudd, J.L., et al. (2025). HMGA2 Expression Predicts Subtype, Survival, and Treatment Outcome in Pancreatic Ductal Adenocarcinoma. Clin. Cancer Res. *31*, 733–745. https://doi.org/10.1158/1078-0432.CCR-24-2200.
39. Schoeps, B., Frädrich, J., and Krüger, A. (2023). Cut loose TIMP-1: an emerging cytokine in inflammation. Trends Cell Biol. *33*, 413–426. https://doi.org/10.1016/j.tcb.2022.08.005.
40. Abdollahi, A., Schwager, C., Kleeff, J., Esposito, I., Domhan, S., Peschke, P., Hauser, K., Hahnfeldt, P., Hlatky, L., Debus, J., et al. (2007). Transcriptional network governing the angiogenic switch in human pancreatic cancer. Proc. Natl. Acad. Sci. USA *104*, 12890–12895. https://doi.org/10.1073/pnas.0705505104.
41. Gandhi, S., de la Fuente, J., Murad, M.H., and Majumder, S. (2022). Chronic Pancreatitis Is a Risk Factor for Pancreatic Cancer, and Incidence Increases With Duration of Disease: A Systematic Review and Meta-analysis. Clin. Transl. Gastroenterol. *13*, e00463. https://doi.org/10.14309/ctg.0000000000000463.
42. Diaferia, G.R., Balestrieri, C., Prosperini, E., Nicoli, P., Spaggiari, P., Zerbi, A., and Natoli, G. (2016). Dissection of transcriptional and cis-regulatory control of differentiation in human pancreatic cancer. EMBO J. *35*, 595–617. https://doi.org/10.15252/embj.201592404.
43. Falcomatà, C., Bärthel, S., Widholz, S.A., Schneeweis, C., Montero, J.J., Toska, A., Mir, J., Kaltenbacher, T., Heetmeyer, J., Swietlik, J.J., et al. (2022). Selective multi-kinase inhibition sensitizes mesenchymal pancreatic cancer to immune checkpoint blockade by remodeling the tumor microenvironment. Nat. Cancer *3*, 318–336. https://doi.org/10.1038/s43018-021-00326-1.
44. Rebuffet, L., Melsen, J.E., Escalière, B., Basurto-Lozada, D., Bhandoola, A., Björkström, N.K., Bryceson, Y.T., Castriconi, R., Cichocki, F., Colonna, M., et al. (2024). High-dimensional single-cell analysis of human natural killer cell heterogeneity. Nat. Immunol. *25*, 1474–1488. https://doi.org/10.1038/s41590-024-01883-0.
45. Huang, C., and Bi, J. (2021). Expression Regulation and Function of T-Bet in NK Cells. Front. Immunol. *12*, 761920. https://doi.org/10.3389/fimmu.2021.761920.
46. Melsen, J.E., van Ostaijen-Ten Dam, M.M., Schoorl, D.J.A., Schol, P.J., van den Homberg, D.A.L., Lankester, A.C., Lugthart, G., and Schilham, M.W. (2022). Single-cell transcriptomics in bone marrow delineates CD56(dim)-GranzymeK(+) subset as intermediate stage in NK cell differentiation. Front. Immunol. *13*, 1044398. https://doi.org/10.3389/fimmu.2022.1044398.
47. Prokopchuk, O., Grünwald, B., Nitsche, U., Jäger, C., Prokopchuk, O.L., Schubert, E.C., Friess, H., Martignoni, M.E., and Krüger, A. (2018). Elevated systemic levels of the matrix metalloproteinase inhibitor TIMP-1 correlate with clinical markers of cachexia in patients with chronic pancreatitis and pancreatic cancer. BMC Cancer *18*, 128. https://doi.org/10.1186/s12885-018-4055-9.
48. Hermann, C.D., Schoeps, B., Eckfeld, C., Munkhbaatar, E., Kniep, L., Prokopchuk, O., Wirges, N., Steiger, K., Häußler, D., Knolle, P., et al. (2021). TIMP1 expression underlies sex disparity in liver metastasis and survival in pancreatic cancer. J. Exp. Med. *218*, e20210911. https://doi.org/10.1084/jem.20210911.
49. Albini, A., Gallazzi, M., Palano, M.T., Carlini, V., Ricotta, R., Bruno, A., Stetler-Stevenson, W.G., and Noonan, D.M. (2021). TIMP1 and TIMP2 Downregulate TGFbeta Induced Decidual-like Phenotype in Natural Killer Cells. Cancers (Basel) *13*, 4955. https://doi.org/10.3390/cancers13194955.
50. Meng, Q., Malinovskii, V., Huang, W., Hu, Y., Chung, L., Nagase, H., Bode, W., Maskos, K., and Brew, K. (1999). Residue 2 of TIMP-1 is a major determinant of affinity and specificity for matrix metalloproteinases but effects of substitutions do not correlate with those of the corresponding P1' residue of substrate. J. Biol. Chem. *274*, 10184–10189. https://doi.org/10.1074/jbc.274.15.10184.

51. Schoeps, B., Eckfeld, C., Flüter, L., Keppler, S., Mishra, R., Knolle, P., Bayerl, F., Böttcher, J., Hermann, C.D., Häußler, D., and Krüger, A. (2021). Identification of invariant chain CD74 as a functional receptor of tissue inhibitor of metalloproteinases-1 (TIMP-1). J. Biol. Chem. *297*, 101072. https://doi.org/10.1016/j.jbc.2021.101072.

52. Lambert, J., Oc, S., Worssam, M.D., Häußler, D., Solomon, C.U., Figg, N.L., Baxter, R., Imaz, M., Taylor, J.C.K., Foote, K., et al. (2024). Network-based prioritization and validation of regulators of vascular smooth muscle cell proliferation in disease. Nat. Cardiovasc. Res. *3*, 714–733. https://doi.org/10.1038/s44161-024-00474-4.

53. Marçais, A., Marotel, M., Degouve, S., Koenig, A., Fauteux-Daniel, S., Drouillard, A., Schlums, H., Viel, S., Besson, L., Allatif, O., et al. (2017). High mTOR activity is a hallmark of reactive natural killer cells and amplifies early signaling through activating receptors. eLife *6*, e26423. https://doi.org/10.7554/eLife.26423.

54. Gasteiger, G., Hemmers, S., Firth, M.A., Le Floc'h, A., Huse, M., Sun, J.C., and Rudensky, A.Y. (2013). IL-2-dependent tuning of NK cell sensitivity for target cells is controlled by regulatory T cells. J. Exp. Med. *210*, 1167–1178. https://doi.org/10.1084/jem.20122462.

55. Marçais, A., Cherfils-Vicini, J., Viant, C., Degouve, S., Viel, S., Fenis, A., Rabilloud, J., Mayol, K., Tavares, A., Bienvenu, J., et al. (2014). The metabolic checkpoint kinase mTOR is essential for IL-15 signaling during the development and activation of NK cells. Nat. Immunol. *15*, 749–757. https://doi.org/10.1038/ni.2936.

56. Gotthardt, D., and Sexl, V. (2016). STATs in NK-Cells: The Good, the Bad, and the Ugly. Front. Immunol. *7*, 694. https://doi.org/10.3389/fimmu.2016.00694.

57. Esfahani, K., Roudaia, L., Buhlaiga, N., Del Rincon, S.V., Papneja, N., and Miller, W.H., Jr. (2020). A review of cancer immunotherapy: from the past, to the present, to the future. Curr. Oncol. *27*, S87–S97. https://doi.org/10.3747/co.27.5223.

58. Balachandran, V.P., Beatty, G.L., and Dougan, S.K. (2019). Broadening the Impact of Immunotherapy to Pancreatic Cancer: Challenges and Opportunities. Gastroenterology *156*, 2056–2072. https://doi.org/10.1053/j.gastro.2018.12.038.

59. Schoeps, B., Eckfeld, C., Prokopchuk, O., Böttcher, J., Häußler, D., Steiger, K., Demir, I.E., Knolle, P., Soehnlein, O., Jenne, D.E., et al. (2021). TIMP1 Triggers Neutrophil Extracellular Trap Formation in Pancreatic Cancer. Cancer Res. *81*, 3568–3579. https://doi.org/10.1158/0008-5472.CAN-20-4125.

60. Batlle, E., and Massagué, J. (2019). Transforming Growth Factor-beta Signaling in Immunity and Cancer. Immunity *50*, 924–940. https://doi.org/10.1016/j.immuni.2019.03.024.

61. Shurin, M.R. (2018). Osteopontin controls immunosuppression in the tumor microenvironment. J. Clin. Investig. *128*, 5209–5212. https://doi.org/10.1172/JCI124918.

62. Hirani, P., Gauthier, V., Allen, C.E., Wight, T.N., and Pearce, O.M.T. (2021). Targeting Versican as a Potential Immunotherapeutic Strategy in the Treatment of Cancer. Front. Oncol. *11*, 712807. https://doi.org/10.3389/fonc.2021.712807.

63. Swietlik, J.J., Bärthel, S., Falcomatà, C., Fink, D., Sinha, A., Cheng, J., Ebner, S., Landgraf, P., Dieterich, D.C., Daub, H., et al. (2023). Cell-selective proteomics segregates pancreatic cancer subtypes by extracellular proteins in tumors and circulation. Nat. Commun. *14*, 2642. https://doi.org/10.1038/s41467-023-38171-8.

64. Prokopchuk, O., Hermann, C.D., Schoeps, B., Nitsche, U., Prokopchuk, O.L., Knolle, P., Friess, H., Martignoni, M.E., and Krüger, A. (2021). A novel tissue inhibitor of metalloproteinases-1/liver/cachexia score predicts prognosis of gastrointestinal cancer patients. J. Cachexia Sarcopenia Muscle *12*, 378–392. https://doi.org/10.1002/jcsm.12680.

65. Cheng, G., Fan, X., Hao, M., Wang, J., Zhou, X., and Sun, X. (2016). Higher levels of TIMP-1 expression are associated with a poor prognosis in triple-negative breast cancer. Mol. Cancer *15*, 30. https://doi.org/10.1186/s12943-016-0515-5.

66. Priego, N., de Pablos-Aragoneses, A., Perea-García, M., Pieri, V., Hernández-Oliver, C., Álvaro-Espinosa, L., Rojas, A., Sánchez, O., Steindl, A., Caleiras, E., et al. (2025). TIMP1 Mediates Astrocyte-Dependent Local Immunosuppression in Brain Metastasis Acting on Infiltrating CD8+ T Cells. Cancer Discov. *15*, 179–201. https://doi.org/10.1158/2159-8290.CD-24-0134.

67. Netskar, H., Pfefferle, A., Goodridge, J.P., Sohlberg, E., Dufva, O., Teichmann, S.A., Brownlie, D., Michaëlsson, J., Marquardt, N., Clancy, T., et al. (2024). Pan-cancer profiling of tumor-infiltrating natural killer cells through transcriptional reference mapping. Nat. Immunol. *25*, 1445–1459. https://doi.org/10.1038/s41590-024-01884-z.

68. Zhu, G.Q., Tang, Z., Huang, R., Qu, W.F., Fang, Y., Yang, R., Tao, C.Y., Gao, J., Wu, X.L., Sun, H.X., et al. (2023). CD36(+) cancer-associated fibroblasts provide immunosuppressive microenvironment for hepatocellular carcinoma via secretion of macrophage migration inhibitory factor. Cell Discov. *9*, 25. https://doi.org/10.1038/s41421-023-00529-z.

69. Ka-Yue Chow, L., Lai-Shun Chung, D., Tao, L., Chan, K.F., Tung, S.Y., Cheong Ngan, R.K., Ng, W.T., Wing-Mui Lee, A., Yau, C.C., Lai-Wan Kwong, D., et al. (2022). Epigenomic landscape study reveals molecular subtypes and EBV-associated regulatory epigenome reprogramming in nasopharyngeal carcinoma. EBioMedicine *86*, 104357. https://doi.org/10.1016/j.ebiom.2022.104357.

70. Oelmann, E., Herbst, H., Zühlsdorf, M., Albrecht, O., Nolte, A., Schmitmann, C., Manzke, O., Diehl, V., Stein, H., and Berdel, W.E. (2002). Tissue inhibitor of metalloproteinases 1 is an autocrine and paracrine survival factor, with additional immune-regulatory functions, expressed by Hodgkin/Reed-Sternberg cells. Blood *99*, 258–267. https://doi.org/10.1182/blood.v99.1.258.

71. Eckfeld, C., Schoeps, B., Häußler, D., Frädrich, J., Bayerl, F., Böttcher, J.P., Knolle, P., Heisz, S., Prokopchuk, O., Hauner, H., et al. (2023). TIMP-1 is a novel ligand of Amyloid Precursor Protein and triggers a proinflammatory phenotype in human monocytes. J. Cell Biol. *222*, e202206095. https://doi.org/10.1083/jcb.202206095.

72. Marcon, F., Zuo, J., Pearce, H., Nicol, S., Margielewska-Davies, S., Farhat, M., Mahon, B., Middleton, G., Brown, R., Roberts, K.J., and Moss, P. (2020). NK cells in pancreatic cancer demonstrate impaired cytotoxicity and a regulatory IL-10 phenotype. OncoImmunology *9*, 1845424. https://doi.org/10.1080/2162402X.2020.1845424.

73. Yoon, J.H., Yoon, H.N., Kang, H.J., Yoo, H., Choi, M.J., Chung, J.Y., Seo, M., Kim, M., Lim, S.O., Kim, Y.J., et al. (2024). Empowering pancreatic tumor homing with augmented anti-tumor potency of CXCR2-tethered CAR-NK cells. Mol. Ther. Oncol. *32*, 200777. https://doi.org/10.1016/j.omton.2024.200777.

74. Malchiodi, Z.X., Lekan, A.A., Suter, R.K., Deshpande, A., Peran, I., Harris, B.T., Duttargi, A., Chien, M.J., Hariharan, S., Wetherill, L., et al. (2025). NK cell co-localization with epithelial cells in pancreatic cancer is influenced by fibroblasts and ECM components. bioRxiv. https://doi.org/10.1101/2024.05.23.593868.

75. Fitzgerald, A.A., Wang, S., Agarwal, V., Marcisak, E.F., Zuo, A., Jablonski, S.A., Loth, M., Fertig, E.J., MacDougall, J., Zhukovsky, E., et al. (2021). DPP inhibition alters the CXCR3 axis and enhances NK and CD8+ T cell infiltration to improve anti-PD1 efficacy in murine models of pancreatic ductal adenocarcinoma. J. Immunother. Cancer *9*, e002837. https://doi.org/10.1136/jitc-2021-002837.

76. Häußler, D., Manevski, D., Frädrich, J., Brunner, V., Prokopchuk, O., Sommer, A., Toledo, B., Knolle, P., Martignoni, M.E., Friess, H., et al. (2025). Extent of N-glycosylation of the metalloproteinase inhibitor and cytokine TIMP-1 determines pancreatic cancer cell proliferation and survival via CD63. J. Biol. Chem. *301*, 110211. https://doi.org/10.1016/j.jbc.2025.110211.

77. Rothfuss, C., Baumann, T., Donakonda, S., Brauchle, B., Marcinek, A., Urban, C., Mergner, J., Pedde, A.M., Hirschberger, A., Krupka, C., et al. (2025). Two-layered immune escape in AML is overcome by Fcgamma

receptor activation and inhibition of PGE2 signaling in NK cells. Blood *145*, 1395–1406. https://doi.org/10.1182/blood.2024025706.

78. Galaxy Community (2024). The Galaxy platform for accessible, reproducible, and collaborative data analyses: 2024 update. Nucleic Acids Res. *52*, W83–W94. https://doi.org/10.1093/nar/gkae410.
79. Andrews, S. (2010). FastQC: a quality control tool for high throughput sequence data. https://www.bioinformatics.babraham.ac.uk/projects/fastqc/.
80. Martin, M. (2011). Cutadapt removes adapter sequences from high-throughput sequencing reads. EMBnet. j. *17*, 10. https://doi.org/10.14806/ej.17.1.200.
81. Dobin, A., Davis, C.A., Schlesinger, F., Drenkow, J., Zaleski, C., Jha, S., Batut, P., Chaisson, M., and Gingeras, T.R. (2013). STAR: ultrafast universal RNA-seq aligner. Bioinformatics *29*, 15–21. https://doi.org/10.1093/bioinformatics/bts635.
82. Liao, Y., Smyth, G.K., and Shi, W. (2014). featureCounts: an efficient general purpose program for assigning sequence reads to genomic features. Bioinformatics *30*, 923–930. https://doi.org/10.1093/bioinformatics/btt656.
83. Ewels, P., Magnusson, M., Lundin, S., and Käller, M. (2016). MultiQC: summarize analysis results for multiple tools and samples in a single report. Bioinformatics *32*, 3047–3048. https://doi.org/10.1093/bioinformatics/btw354.
84. Love, M.I., Huber, W., and Anders, S. (2014). Moderated estimation of fold change and dispersion for RNA-seq data with DESeq2. Genome Biol. *15*, 550. https://doi.org/10.1186/s13059-014-0550-8.
85. Subramanian, A., Tamayo, P., Mootha, V.K., Mukherjee, S., Ebert, B.L., Gillette, M.A., Paulovich, A., Pomeroy, S.L., Golub, T.R., Lander, E.S., and Mesirov, J.P. (2005). Gene set enrichment analysis: a knowledge-based approach for interpreting genome-wide expression profiles. Proc. Natl. Acad. Sci. USA *102*, 15545–15550. https://doi.org/10.1073/pnas.0506580102.
86. Shannon, P., Markiel, A., Ozier, O., Baliga, N.S., Wang, J.T., Ramage, D., Amin, N., Schwikowski, B., and Ideker, T. (2003). Cytoscape: a software environment for integrated models of biomolecular interaction networks. Genome Res. *13*, 2498–2504. https://doi.org/10.1101/gr.1239303.
87. Newman, A.M., Steen, C.B., Liu, C.L., Gentles, A.J., Chaudhuri, A.A., Scherer, F., Khodadoust, M.S., Esfahani, M.S., Luca, B.A., Steiner, D., et al. (2019). Determining cell type abundance and expression from bulk tissues with digital cytometry. Nat. Biotechnol. *37*, 773–782. https://doi.org/10.1038/s41587-019-0114-2.
88. ParseBiosciences (2024). Trailmaker™. http://app.trailmaker.parsebiosciences.com.
89. Bergen, V., Lange, M., Peidli, S., Wolf, F.A., and Theis, F.J. (2020). Generalizing RNA velocity to transient cell states through dynamical modeling. Nat. Biotechnol. *38*, 1408–1414. https://doi.org/10.1038/s41587-020-0591-3.
90. La Manno, G., Soldatov, R., Zeisel, A., Braun, E., Hochgerner, H., Petukhov, V., Lidschreiber, K., Kastriti, M.E., Lönnerberg, P., Furlan, A., et al. (2018). RNA velocity of single cells. Nature *560*, 494–498. https://doi.org/10.1038/s41586-018-0414-6.
91. Andreatta, M., and Carmona, S.J. (2021). UCell: Robust and scalable single-cell gene signature scoring. Comput. Struct. Biotechnol. J. *19*, 3796–3798. https://doi.org/10.1016/j.csbj.2021.06.043.
92. Kuleshov, M.V., Jones, M.R., Rouillard, A.D., Fernandez, N.F., Duan, Q., Wang, Z., Koplev, S., Jenkins, S.L., Jagodnik, K.M., Lachmann, A., et al. (2016). Enrichr: a comprehensive gene set enrichment analysis web server 2016 update. Nucleic Acids Res. *44*, W90–W97. https://doi.org/10.1093/nar/gkw377.
93. Jin, S., Guerrero-Juarez, C.F., Zhang, L., Chang, I., Ramos, R., Kuan, C.H., Myung, P., Plikus, M.V., and Nie, Q. (2021). Inference and analysis of cell-cell communication using CellChat. Nat. Commun. *12*, 1088. https://doi.org/10.1038/s41467-021-21246-9.
94. Zheng, S., Wang, W., Aldahdooh, J., Malyutina, A., Shadbahr, T., Tanoli, Z., Pessia, A., and Tang, J. (2022). SynergyFinder Plus: Toward Better Interpretation and Annotation of Drug Combination Screening Datasets. Genom. Proteom. Bioinform. *20*, 587–596. https://doi.org/10.1016/j.gpb.2022.01.004.
95. Galili, T. (2015). dendextend: an R package for visualizing, adjusting and comparing trees of hierarchical clustering. Bioinformatics *31*, 3718–3720. https://doi.org/10.1093/bioinformatics/btv428.
96. Gray, B. (2024). Subdistribution Analysis of Competing Risks. https://cran.r-project.org/web/packages/cmprsk/cmprsk.pdf.
97. Gorfine, M., Schlesinger, M., and Hsu, L. (2020). K-sample omnibus non-proportional hazards tests based on right-censored data. Stat. Methods Med. Res. *29*, 2830–2850. https://doi.org/10.1177/0962280220907355.
98. Han, K., and Jung, I. (2022). Restricted Mean Survival Time for Survival Analysis: A Quick Guide for Clinical Researchers. Korean J. Radiol. *23*, 495–499. https://doi.org/10.3348/kjr.2022.0061.
99. Korsunsky, I., Nathan, A., Millard, N., and Raychaudhuri, S. (2019). Presto scales Wilcoxon and auROC analyses to millions of observations. bioRxiv. https://doi.org/10.1101/653253.
100. Ritchie, M.E., Phipson, B., Wu, D., Hu, Y., Law, C.W., Shi, W., and Smyth, G.K. (2015). limma powers differential expression analyses for RNA-sequencing and microarray studies. Nucleic Acids Res. *43*, e47. https://doi.org/10.1093/nar/gkv007.
101. Tu, J., Huang, Z., Wang, Y., Wang, M., Yin, Z., Mei, X., Li, M., and Li, L. (2021). Transcriptome analysis of the procession from chronic pancreatitis to pancreatic cancer and metastatic pancreatic cancer. Sci. Rep. *11*, 3409. https://doi.org/10.1038/s41598-021-83015-4.
102. Batut, B.F., M.; Heydarian, M.; Erxleben, A.; Videm, P.; Blank, C.; Doyle, M; Soranzo, N.; van Heusden, P.; Delisle, L. Reference-based RNA-Seq data analysis (Galaxy Training Materials). https://training.galaxyproject.org/training-material/topics/transcriptomics/tutorials/ref-based/tutorial.html.
103. Reimand, J., Isserlin, R., Voisin, V., Kucera, M., Tannus-Lopes, C., Rostamianfar, A., Wadi, L., Meyer, M., Wong, J., Xu, C., et al. (2019). Pathway enrichment analysis and visualization of omics data using g:Profiler, GSEA, Cytoscape and EnrichmentMap. Nat. Protoc. *14*, 482–517. https://doi.org/10.1038/s41596-018-0103-9.
104. Liberzon, A., Subramanian, A., Pinchback, R., Thorvaldsdóttir, H., Tamayo, P., and Mesirov, J.P. (2011). Molecular signatures database (MSigDB) 3.0. Bioinformatics *27*, 1739–1740. https://doi.org/10.1093/bioinformatics/btr260.
105. Crinier, A., Milpied, P., Escaliere, B., Piperoglou, C., Galluso, J., Balsamo, A., Spinelli, L., Cervera-Marzal, I., Ebbo, M., Girard-Madoux, M., et al. (2018). High-Dimensional Single-Cell Analysis Identifies Organ-Specific Signatures and Conserved NK Cell Subsets in Humans and Mice. Immunity *49*, 971–986.e975. https://doi.org/10.1016/j.immuni.2018.09.009.
106. Collins, P.L., Cella, M., Porter, S.I., Li, S., Gurewitz, G.L., Hong, H.S., Johnson, R.P., Oltz, E.M., and Colonna, M. (2019). Gene Regulatory Programs Conferring Phenotypic Identities to Human NK Cells. Cell *176*, 348–360.e12. https://doi.org/10.1016/j.cell.2018.11.045.
107. Smith, S.L., Kennedy, P.R., Stacey, K.B., Worboys, J.D., Yarwood, A., Seo, S., Solloa, E.H., Mistretta, B., Chatterjee, S.S., Gunaratne, P., et al. (2020). Diversity of peripheral blood human NK cells identified by single-cell RNA sequencing. Blood Adv. *4*, 1388–1406. https://doi.org/10.1182/bloodadvances.2019000699.
108. Jung, K.K., Liu, X.W., Chirco, R., Fridman, R., and Kim, H.R.C. (2006). Identification of CD63 as a tissue inhibitor of metalloproteinase-1 interacting cell surface protein. EMBO J. *25*, 3934–3942. https://doi.org/10.1038/sj.emboj.7601281.
109. Yadav, B., Wennerberg, K., Aittokallio, T., and Tang, J. (2015). Searching for Drug Synergy in Complex Dose-Response Landscapes Using an Interaction Potency Model. Comput. Struct. Biotechnol. J. *13*, 504–513. https://doi.org/10.1016/j.csbj.2015.09.001.

STAR★METHODS

KEY RESOURCES TABLE

REAGENT or RESOURCE	SOURCE	IDENTIFIER
Antibodies		
TIMP-1 anti-human mAb (rabbit)	Cell Signaling	CAT# 8946; clone D10E6; RRID: AB_10891805
HRP-conjugated anti-rabbit IgG (H + L) polyclonal Ab (goat)	ThermoFisher Scientific	CAT# 31462; RRID: AB_228338
AlexaFluor Plus 405-conjugated anti-rabbit IgG, (H + L), polyclonal Ab (goat)	ThermoFisher Scientific	CAT# A48254; RRID: AB_2890548
CD56 PE-Cyanine7 anti-human mAb (mouse)	BioLegend	CAT# 398808; clone QA18A21; RRID: AB_2894510
CD56 anti-human Alexa Fluor 488, mAB (mouse)	BioLegend	CAT# 362517; RRID: AB_2564092
CD3 eFluor450 anti-human, mAb (mouse)	ThermoFisher Scientific	CAT# 48-0032-82; clone OKT3; RRID: AB_1272193
CD74 mAb milatuzumab	ThermoFisher Scientific	CAT# MA5-41757; RRID: AB_2910900
IgG Isotype control human, polyclonal Ab	ThermoFisher Scientific	CAT# 02-7102; RRID: AB_2532958
granzyme B PE anti-human, mAb (mouse)	ThermoFisher Scientific	CAT# 12-8896-42; clone GB11; RRID: AB_2724394
Perforin PE anti-human, mAb (mouse)	Biolegend	CAT# 353304; clone B-D48; RRID: AB_2616860
mTOR *p*-Ser2448 PE anti-human/mouse, mAb (mouse)	ThermoFisher Scientific	CAT# 12-9718-42; clone MRRBY; RRID: AB_2572724
S6 p-Ser235/236 PerCP-eFluor710 anti-human/mouse, mAB (mouse)	ThermoFisher Scientific	CAT# 46-9007-42; clone cupk43k; RRID: AB_2573858
STAT3 p-Tyr705 FITC anti-human/mouse, mAb (mouse)	ThermoFisher Scientific	CAT# 11-9033-42; clone LUVNKLA; RRID: AB_2572522
IFN-γ FITC anti-human, mAb (mouse)	ThermoFisher Scientific	CAT# 11-7319-82; clone 4S.B3; RRID: AB_465415
TNF-a PE anti-human, mAb (mouse)	BD Pharmingen	CAT# 559321; clone Mab11; RRID: AB_397219
CD107a PerCP-Cyanine5.5 anti-human, mAb (mouse)	BioLegend	CAT# 328615; clone H4A3; RRID: AB_1227509
Biological samples		
PDAC patient samples TUM Cohort	This study	N/A
KPC mouse pancreas samples	This study	N/A
Human blood samples	This study	N/A
Chemicals, peptides, and recombinant proteins		
β-mercaptoethanol	PanReac AppliChem	CAT# 60-24-2
Nuclease-free water	ThermoFisher Scientific	CAT# 10977035
Trypan Blue	ThermoFisher Scientific	CAT# 15250061
RPMI1640	ThermoFisher Scientific	CAT# 11875093
Penicillin-Streptomycin	ThermoFisher Scientific	CAT# 15140122
Fetal Bovine Serum	ThermoFisher Scientific	CAT# A5209502
eBioscience™ Cell Proliferation Dye eFluor450	ThermoFisher Scientific	CAT# 65-0842-85
Trametinib	Selleckchem	CAT# S2673
Nintedanib	Sigma-Aldrich	CAT# SML2848
Brefeldin A	ThermoFisher Scientific	CAT# 00-4506-51
SYPRO Ruby Protein stain	ThermoFisher Scientific	CAT# S11791
IC Fixation Buffer	ThermoFisher Scientific	CAT# 00-8222-49
PolymorphPrep™	Progen	CAT# 1895
Recombinant TIMP-1 variants	Schoeps et al.[59]	N/A
Batimastat	Sigma-Aldrich	CAT# SML0041
TruStain FcX™	BioLegend	CAT# 422302
Recombinant human IL-2	BioLegend	CAT# 589104
HCS LipidTOX™ Red Neutral Lipid Stain	ThermoFisher Scientific	CAT# H34476
Annexin V FITC	BioLegend	CAT# 640906
eBioscience 7-AAD viability dye	ThermoFisher Scientific	CAT# 00-6993-50

(*Continued on next page*)

CellPress
OPEN ACCESS

Continued

REAGENT or RESOURCE	SOURCE	IDENTIFIER
**Critical commercial assays**		
RNeasy Plus Mini Kit	Qiagen	CAT# 74104
Chromium Nuclei Isolation Kit with RNase Inhibitor	10x Genomics	CAT# 1000494
Chromium Next GEM Single Cell 3′ Kit v3.1	10x Genomics	CAT# 1000268
Chromium Dual Index Kit TT Set A	10x Genomics	CAT# 1000215
Chromium Next GEM Chip G Single Cell Kit	10x Genomics	CAT# 1000120
HS DNA Kit	Agilent	CAT# 5067-4626
MojoSort^{TM} Human NK Cell Isolation Kit	BioLegend	CAT# 480054
Invitrogen^{TM} Intracellular Fixation & Permeabilization Buffer Set	ThermoFisher Scientific	CAT# 88-8824-00
**Deposited data**		
PDAC patient snRNA sequencing	This study	GEO: GSE291124; SRA: PRJNA1230557
KPC pancreatic tissue bulk RNA sequencing	This study	GEO: GSE290898
MIA PaCa-2 bulk RNA sequencing	This study	GEO: GSE290413
PDAC patient scRNA sequencing	Steele et al.[22]	GEO: GSE155698
PDAC patient proteome	Cao et al.[31]	CPTAC data portal: PDC000270
Pancreatic tissue (pancreatic tumors, chronic pancreatitis, and healthy donors) transcription profiling by array	Abdollahi et al.[40]	EBI ArrayExpress: E-EMBL-6
Patient tumor sample bulk RNA sequencing	TCGA	http://firebrowse.org; BLCA, BRCA, COADREAD, GBM, HNSC, KIPAN, LIHC, LUAD, PAAD, PRAD, SKCM, STES, THCA
Murine orthotopic PDAC scRNA sequencing	Falcomata, Bärthel et al.[43]	EBI ArrayExpress: E-MTAB-9954
Murine pancreatitis bulk RNA sequencing (day 1 and day 7 after cerulein-induced pancreatitis)	Del-Poggetto et al.[27]	GEO: GSE180212
Murine pancreatitis (day 2 after cerulein-induced pancreatitis) and PDAC bulk RNA sequencing	Alonso-Curbelo et al.[26]	GEO: GSE132330
PC cell lines microarray	Moffitt et al.[19]	GEO: GSE71729
PC cell line bulk RNA sequencing	Diaferia et al.[42]	GEO: GSE64558
**Experimental models: cell lines**		
MIA PaCa-2	ATCC	CRL-1420
CRISPR-Cas9-derived MIA PaCa-2 cell lines	Häußler et al.[76]; This study	N/A
K562	Rothfuß et al.[77]	N/A
**Experimental models: organisms/strains**		
Mouse C57BL/6 Pdx-1$^{+/Cre}$; Kras$^{+/LSL-G12D}$; Trp53$^{+/LSL-R172H}$	Hingorani et al.[30]	N/A
**Software and algorithms**		
Biological DataBase network	bioDBnet	https://biodbnet-abcc.ncifcrf.gov/db/dbOrtho.php
GraphPad Prism v.10.4.1	GraphPad	https://www.graphpad.com/
FlowJo v.10.10.0	BD Biosciences	https://www.flowjo.com
R and RStudio v.4.4.2	R Consortium	https://posit.co/products/open-source/rstudio/
Galaxy v24.0	Galaxy Project[78]	https://usegalaxy.eu
FastQC v.0.12.1	Andrews[79]	https://github.com/s-andrews/FastQC
Cutadapt v.5.1	Martin[80]	https://github.com/marcelm/cutadapt
STAR v.2.7.11b	Dobin et al.[81]	https://github.com/alexdobin/STAR/releases
featureCounts	Liao et al.[82]	https://scienceparkstudygroup.github.io/ibed-bioinformatics-page/source/core_tools/featurecounts.html
MultiQC v.1.27	Ewels et al.[83]	https://github.com/orgs/MultiQC/repositories

(*Continued on next page*)

Continued

REAGENT or RESOURCE	SOURCE	IDENTIFIER
DESeq2 v.1.40.2	Love et al.[84]	https://github.com/thelovelab/DESeq2
GSEA v.4.2.3	Subramanian et al.[85]	https://www.gsea-msigdb.org/gsea/index.jsp
Cytoscape v.3.9.1	Shannon et al.[86]	https://cytoscape.org/
CIBERSORTx platform	Newman et al.[87]	https://cibersortx.stanford.edu/index.php
Trailmaker	ParseBiosciences[88]	http://app.trailmaker.parsebiosciences.com
Cell Ranger v7.0.1 and v9.0.1	10x Genomics	https://www.10xgenomics.com/support/software/cell-ranger/latest
scVelo v.0.3.3	Bergen et al.[89]	https://github.com/theislab/scvelo
velocyto v.0.17.17	La Manno et al.[90]	https://github.com/velocyto-team/velocyto.py
Python v3.12.7	Python Software Foundation	https://www.python.org/
Seurat-Disk v.0.0.0.9021	Paul Hoffman	https://mojaveazure.github.io/seurat-disk/
UCell v.2.10.1	Andreatta et al.[91]	https://github.com/carmonalab/UCell
inferCNV v.1.18.1	Broad Institute	https://github.com/broadinstitute/infercnv)
Enrichr	Kuleshov et al.[92]	https://maayanlab.cloud/Enrichr/
CellChat	Jin et al.[93]	https://github.com/sqjin/CellChat
SynergyFinder+	Zheng et al.[94]	synergyfinder.org
Affinity Designer v.1.10.8	Affinity	https://affinity.serif.com/de/designer/
Venny 2.1	BioinfoGP	https://bioinfogp.cnb.csic.es/tools/venny/
R dendextend package v.1.19.1	Galili[95]	https://talgalili.github.io/dendextend/
R cmprsk package v.2.2–12	Gray[96]	https://cran.r-universe.dev/cmprsk
R KONPsurv package v1.0.4	Gorfine, Schlesinger et al.[97]	https://cran.r-universe.dev/KONPsurv
R survRM2 package v1.0-4	Han et al.[98]	https://cran.r-universe.dev/survRM2
R presto package v.1.0.0	Korsunsky et al.[99]	https://github.com/immunogenomics/presto
R limma package v.3.50.3	Ritchie et al.[100]	https://www.bioconductor.org/packages/release/bioc/html/limma.html

EXPERIMENTAL MODEL AND STUDY PARTICIPANT DETAILS

PDAC patients and blood donors

Ethical approval for PDAC patients (TUM Cohort) and healthy blood donors enrolled in this study was obtained from the ethics committee of the Medical Faculty of the Technical University of Munich, Germany (TUM Cohort: ‘2023-245-S-KH’; healthy blood donors: ‘#183/18S’, ‘#409/16S’, ‘#395/17S’). Female and male PDAC patients and blood donors were enrolled. Written consent was obtained from all individuals before surgery or blood sampling. The analysis was performed on pseudonymized patient information. Primary tumor samples were collected from treatment-naïve patients with PDAC who underwent surgical resection between 2015 and 2023 in the Department of Surgery (TUM Universitätsklinikum rechts der Isar). Diagnosis of PDAC was verified by definitive histological examination of surgical specimens or retrieved biopsies. Resected tumor samples were snap frozen and stored at -80°C before processing for snRNA-seq.

Animal models

Animal experiments were performed according to the Animal Research Reporting of *in vivo* experiment guidelines and in compliance with the *Tierschutzgesetz des Freistaates Bayern* and upon ethical approval by the *Regierung von Oberbayern* (‘Vet_02-20-80’). Both female and male mice were employed in this study and kept at the animal facility of TUM Universitätsklinikum rechts der Isar (Munich, Germany) at RT (21°C) under specific pathogen-free conditions. Mice were maintained in filter-topped cages with autoclaved food and water.

Mice of the transgenic KPC (Pdx-1$^{+/Cre}$; Kras$^{+/LSL-G12D}$; Trp53$^{+/LSL-R172H}$; C57BL/6 background)[30] PDAC model were employed as described previously.[48,59] We ensured comparability of KPC-derived pancreatic tissues with histologically identical PDAC progression by classification and grading of murine PDAC lesions according to the most recent consensus classification by experienced comparative pathologist (K. Steiger) blinded to sample identity. KPC mice with PDAC grades G2, G3, or G4 were classified as advanced-stage PDAC, whereas mice with pancreatic remodeling and preinvasive lesions but without invasive PDAC G2/G3/G4 lesions were classified as early PDAC. Mice resulting from KPC strain breeding, which did not carry Pdx-1$^{+/Cre}$ but only Kras$^{+/LSL-G12D}$ and/or Trp53$^{+/LSL-R172H}$-mutations, or which carried no mutation at all, served as control animals. Pancreatic tissues were harvested and

stored at −80°C until further use. Data from orthotopic PDAC transplantation mouse models and *in vivo* treatments were derived from animal experiments we had previously conducted and reported.[43] The scRNA-seq dataset obtained from orthotopic PDAC transplantation models has been previously deposited in the EBIArrayExpress repository (accession number: E-MTAB-9954).

Cell lines

The human PDAC cell line MIA PaCa-2 was obtained commercially from ATCC (CRL-1420). The NK-sensitive target cell line K562 was kindly provided by Bastian Höchst (Institute of Molecular Immunology, School of Medicine and Health, Technical University of Munich). No further cell line authentication was performed. All cell lines were cultured in RPMI 1640 (ThermoFisher Scientific) supplemented with 10% FCS (ThermoFisher Scientific) and 1% P/S (ThermoFisher Scientific) at 37°C, 5% CO_2. For the NK cell cytotoxicity assay, K562 cells were labeled using the eBioscience Cell Proliferation Dye eFluor450 (1:5000; ThermoFisher Scientific) according to the manufacturer's instructions. For the NK cell signaling assays in co-culture, MIA PaCa-2 cells were labeled using the same eFluor450 labeling protocol, accordingly.

METHOD DETAILS

Steele Cohort data retrieval

The Steele Cohort comprised pancreatic tumor samples from 16 PDAC patients and tumor-adjacent/normal pancreas control samples from three donors.[22] Deidentified scRNA-seq data from pancreatic tumor and control samples was retrieved from the NIH Gene Expression Omnibus (GEO) database (accession number: GSE155698).

Cao Cohort data retrieval

The Cao Cohort comprised pancreatic tumor samples from 140 PDAC patients, 67 tumor-adjacent tissue control samples, and 8 normal pancreas control samples.[31] Log2 normalized proteome data was retrieved from the CPTAC data portal (accession number: PDC000270).

Abdollahi Cohort data retrieval

The Abdollahi Cohort[40] comprised pancreatic tissue samples from 9 patients with pancreatic tumors, 9 patients with chronic pancreatitis, and 9 healthy controls (www.ebi.ac.uk/arrayexpress, accession number: E-EMBL-6). Processed gene expression profiling data was retrieved from ref.[101]

The Cancer Genome Atlas (TCGA) cohorts

Bulk RNA-seq data of tumor samples from the TCGA Cohorts were retrieved from the FireBrowse database (http://firebrowse.org; 'illuminahiseq_rnaseqv2-RSEM_genes_normalized' files). The cohorts comprised patients with bladder urothelial carcinoma (BLCA), breast invasive carcinoma (BRCA), colorectal adenocarcinoma (COADREAD), glioblastoma multiforme (GBM), head and neck squamous cell carcinoma (HNSC), kidney cancer (KIPAN), liver hepatocellular carcinoma (LIHC), lung adenocarcinoma (LUAD), pancreatic adenocarcinoma (PAAD), prostate adenocarcinoma (PRAD), skin cutaneous melanoma (SKCM), stomach and esophageal carcinoma (STES), or thyroid carcinoma (THCA), respectively. For analysis of gene expression data in each patient cohort, we selected data only from primary tumor samples (sample type code '01', i.e., 'primary solid tumor') and excluded data derived from non-primary tumor samples.

Mouse RNA-seq data retrieval

For RNA-seq analysis of isolated pancreatic epithelial cells from pancreatitis- or PDAC-bearing mice, we retrieved publicly available data from two external cohorts.[26,27] Data from mice at day 1 or day 7 after cerulein-induced pancreatitis were retrieved from the Del-Poggetto Cohort (GEO accession number: GSE180212), data from pancreatitis-bearing mice at day 2 or PDAC-afflicted (i.e., $KP^{fl}C$; Ptf1a-cre; RIK; LSL-$Kras^{G12D}$; $p53^{fl/+}$) mice were retrieved from the Alonso-Curbelo Cohort (GEO accession number: GSE132330). To identify consistently upregulated DEGs across different disease conditions (vs. respective healthy controls as described in the source publications), we (1) extracted all significantly DEGs (up- or dowregulated; adj. p value <0.05) across conditions (visualized as Venn diagram using Venny 2.1; https://bioinfogp.cnb.csic.es/tools/venny/), (2) filtered for DEGs that were always upregulated or always downregulated across all four conditions in both cohorts, and then (3) identified secreted factors among consistently upregulated DEGs by examining the subcellular location of genes (listed as 'secreted' or 'secretory vesicle') using the UniProt database (https://www.uniprot.org; RRID:SCR_002380), as described previously.[48]

Identification of the CISS profile

To identify the CISS gene expression profile in pancreatic tumors (TCGA-PAAD), we (1) converted the 36 secreted factors identified in mice to human gene orthologs using the biological DataBase network (https://biodbnet-abcc.ncifcrf.gov/db/dbOrtho.php), (2) conducted a Spearman correlation analysis of normalized mRNA expression levels of these 36 factors from TCGA-PAAD bulk RNA-seq data, and (3) identified a subset of 19 highly correlating factors within the 36-gene pattern by performing hierarchical clustering of Spearman correlation coefficients using the hclust() function in the base R stats package (R v.4.4.2) and using the dendrogram

function in the dendextend package (v.1.19.1). In each downstream analysis, genes with two aliases (*BAG6* and *BAT3*; *CYR61* and *CCN1; SEPT8* and *SEPTIN8*) were labeled dependent on the respective gene annotation derived from each dataset. The composition of the CISS cluster (19 secreted factors) identified in the TCGA-PAAD Cohort served as a reference for the PDAC proteome validation cohort (Cao Cohort) and the other TCGA cancer cohorts.

For each patient within a cohort, a CISS expression score was calculating by first normalizing individual gene expression for each of the 19 genes to the interval [0,1]. Specifically, we subtracted the minimum gene expression value observed among all patients and divided the result by the corresponding gene's maximum (background-adjusted) value. We then calculated a preliminary score for each patient by taking the mean of these normalized expressions across all 19 genes. Finally, we re-normalized mean values to [0,1] by subtracting the minimum preliminary score (across all patients) and dividing by the resulting maximum. This final CISS score was used for subsequent analyses.

For identification of biological pathways and immune cell abundance associated with CISS expression in pancreatic cancer (Figures 2D–2F), high $CISS^{hi}$ (top 25% quartile of CISS expression) patients were compared to $CISS^{lo}$ (bottom 25% quartile of CISS expression) patients. Differences in gene expression between $CISS^{hi}$ and $CISS^{lo}$ patients was calculated using the Mann-Whitney test and subsequent correction for multiple testing using the two-stage step-up method by Benjamini, Krieger, and Yekutieli (cutoff value: false discovery rate [FDR] q-value = 0.01). $CISS^{hi}$ versus $CISS^{lo}$ fold-change of gene expression was calculated by division of the medians of each population.

Survival analyses

We performed survival analyses for cancer patients in TCGA Cohorts based on clinical metadata from the FireBrowse database (http://firebrowse.org). To estimate cancer-specific survival after primary tumor resection in relation to CISS expression in TCGA Cohorts, we applied a competing risks framework for patients with available survival data, in which death was defined as the event of interest (cause 1) and non-cancer-related deaths were treated as competing events. To quantify associations between CISS and cancer-specific mortality, we employed the *cmprsk* R package (v.2.2–12) to estimate cumulative incidence functions (CIFs) using *cuminc()* and to perform Fine-Gray regression analysis using *crr()*. CIFs and subdistribution hazard ratios (sHRs) were reported with 95% confidence intervals. We used two complementary covariate specifications as follows: (1) Patient-specific CISS score (standardized from 0 to 1 as described above to enable cross-cohort comparability) as a continuous predictor, and (2) CISS groups as a categorical predictor, defined by quartiles of the score ($CISS^{lo}$ for scores from 0 to 0.25; $CISS^{medlo}$ from 0.25 to 0.5; $CISS^{medhi}$ from 0.5 to 0.75; $CISS^{hi}$ from 0.75 to 1), with the $CISS^{lo}$ group as reference. We omitted the PRAD Cohort from CISS-associated survival analyses, as no deaths were documented in the $CISS^{medlo}$, $CISS^{medhi}$, or $CISS^{hi}$ groups, and only 10 deaths were documented in the $CISS^{lo}$ group (n = 423), rendering group-wise competing risks analyses not estimable.

To construct patient-specific risk scores for recurrence-free survival (RFS) and metastasis-free survival (MFS) in relation to CISS expression, TIMP-1 expression, and NK cell activity in the TCGA-PAAD Cohort, we performed Cox proportional hazards regression analyses using GraphPad Prism software (v.10.4.1). RFS was defined as the time from primary tumor resection to any documented recurrence (local or distant), and MFS as the time to specifically distant metastasis. Patients were excluded if: (i) a definite non-cancer cause of death was known, (ii) the time to either event was 0 days, or (iii) recurrence information was unavailable ('NA'). One patient was further excluded as a definite outlier (confirmed via Grubbs' test, alpha = 0.01), due to TIMP-1 transcript levels 45% above all other patients. Patients who died without documented recurrence were censored at the time of death, since the cause of death in these cases was not uniformly annotated. For the final cohort (n = 137), we performed independent Cox regression analyses on four standardized predictors (0–1 as described above): (1) CISS expression, (2) TIMP-1 expression ('T1'), (3) the relative fraction of activated NK cells ('aNK'; see Determining immune cell type abundance with CIBERSORTx), or (4) a combined TIMP1/NK risk score, derived from the patient-specific linear predictor (LP) of a multivariate Cox regression including both 'T1' and 'aNK' as covariates. Patient-specific risk estimates were calculated as hazard ratios by exponentiating the LP values. For the combined TIMP1/NK risk score, patients were stratified into four risk groups according to quartiles: low risk (L) = 35; moderately low risk (ML) = 34; moderately high risk (MH) = 35; high risk (H) = 34).

For descriptive visualization of survival probabilities, Kaplan-Meier curves were plotted using GraphPad Prism software (v.10.4.1). Global differences across patient survival curves were tested with the KONP omnibus test (Cauchy-combo p values, KONPsurv R package v1.0.4). Pairwise comparison between groups was quantified by the restricted mean survival time difference (ΔRMST), estimated to a restriction time τ (set to 0.9 x the minimum of the maximum observed follow-up in the two groups) using survRM2 R package (v1.0-4). Differences in mouse survival curves were calculated using log rank Mantel-Cox test (GraphPad Prism software v.10.4.1).

Bulk RNA-seq of tissues and cell lines

For bulk RNA-seq of murine pancreatic tissues, total RNA was isolated using the RNeasy Plus Mini Kit (Qiagen) following the manufacturer's instructions. Frozen tissue samples ($\leq$30 mg) were lysed in 600 μL RLT buffer supplemented with 10 μL β-mercaptoethanol (PanReac AppliChem) per mL of buffer. Mechanical homogenization using zirconium beads was performed for 5 s to ensure efficient tissue disruption. RNA was eluted in 30 μL of nuclease-free water (ThermoFisher Scientific) and stored at -80°C until further use.

For bulk RNA-seq of human PC cell lines, cells were grown to approximately 70% confluency. Total RNA was isolated using the RNeasy Plus Mini Kit (Qiagen) following the manufacturer's instructions. RNA was eluted in 30 μL of nuclease-free water (ThermoFisher Scientific) and stored at −80°C until further use.

For both murine tissues and human cell lines, bulk RNA-seq dual indexed library preparation was performed by Novogene GmbH using the NGS RNA Library Prep Set (PT042; Novogene GmbH). All barcoded libraries were checked with a Qubit fluorometer (ThermoFisher Scientific) and real-time PCR for quantification, and on a Bioanalyzer (Agilent) for size distribution detection. Quantified libraries were pooled (separately for murine tissues and human cell lines) and sequenced on a NovaSeq X Plus platform (Illumina) with paired-end, 150-bp reads (PE150), targeting 15G raw data per sample (murine tissues), or 9G raw data per sample (human cell lines), respectively.

Bulk RNA-seq data processing

All data processing steps were performed using the Galaxy platform[78] (https://usegalaxy.org; v24.0). FASTQ files were uploaded to Galaxy and quality control for raw reads was performed using the FastQC tool[79] (v.0.12.1) to evaluate read quality, GC content, sequence duplication levels, and adapter contamination. Poor-quality reads and adapter sequences were removed using Cutadapt[80] (v.5.1) with parameters set to trim bases ≤20 bp read length. The reads were mapped to the reference genomes (Mus musculus GRCm39 or Homo sapiens GRCh38) using STAR[81] (v.2.7.11b) and the number of reads per annotated gene were counted using featureCounts.[82] For each step, quality reports were aggregated using MultiQC[83] (v.1.27). DESeq2[84] (v.1.40.2) was used to normalize read counts to extract the DEGs between groups. The complete reproducible workflow can be accessed at https://training.galaxyproject.org/training-material/topics/transcriptomics/tutorials/ref-based/tutorial.html, ref.[102]

Gene Set Enrichment Analyses

Gene Set Enrichment Analyses (GSEA; v.4.2.3)[85] were performed as described previously.[48,103] As reference gene sets, we used C5: GO Biological Processes, C2: Canonical Pathways, and C3: Transcription Factor Targets from the Molecular Signatures Database (MSigDB),[104] as indicated in the respective figure legend. For visualization of GSEA results, we grouped gene sets into broader categories or employed Cytoscape (v.3.9.1) to generate an enrichment map (cutoff values: FDR q-value = 0.05; normalized enrichment score [NES] >1.75 or < -1.75; number of edges per cluster ≥3), as indicated.

Immune cell type abundance using CIBERSORTx

Relative immune cell type abundances in bulk RNA-seq datasets were inferred by digital cytometry using the CIBERSORTx platform[87] (https://cibersortx.stanford.edu/index.php). Normalized gene expression matrices were deconvoluted using standard settings and the LM22 (22 human immune cell subtypes) signature matrix file.

Sample preparation for snRNA-seq

For preparation of single-nucleus suspensions from banked frozen PDAC tumors, approximately 30–60 mg of each tumor were first cut into small pieces using a blade on glass dishes maintained at −80°C (dry ice). Nuclei suspensions were then generated individually using the Chromium Nuclei Isolation Kit with RNase Inhibitor (10x Genomics, 1000494) according to the manufacturer's instructions. After isolation, nuclei were assessed for singularization and integrity using trypan blue staining, quantified (Neubauer chamber), and immediately used for snRNA-seq library preparation.

snRNA-seq library preparation and sequencing

To prepare snRNA-seq libraries, we used the Chromium Next GEM Single Cell 3′ Kit v3.1 (10x Genomics, 1000268) with the Chromium Next GEM Chip G Single Cell Kit (10x Genomics, 1000120), and the Dual Index Kit TT Set A (10x Genomics, 1000215) according to the manufacturer's instructions. Per sample, approximately 20,000 to 35,000 individual nuclei were loaded per lane on a 10x Chromium chip to generate gel beads in emulsion (GEMs) and target a single-nucleus resolution of 12,500 to 22,000 individual nuclei. cDNA and sequencing libraries were assessed for sample size and quality using the HS DNA Kit (Agilent) on an Agilent Bioanalyzer 2100. The prepared single-nucleus gene expression libraries were sequenced on an Illumina NovaSeq 6000 (Novogene GmbH, PE150-S4; paired end, dual index) targeting 50,000 read pairs per nucleus.

snRNA-seq data preprocessing and quality control

We performed alignment of the snRNA-seq data to the human reference genome (GRCh38-2020-A), cell filtering, and counting of barcodes and unique molecular identifiers using the 10x Genomics Cell Ranger software v7.0.1 with intronic reads included. Filtered feature-barcode matrices were then controlled for quality, processed, explored, and visualized using Trailmaker (https://app.trailmaker.parsebiosciences.com/)[88] according to the developers' recommendations. Matrices were uploaded to Trailmaker, and a set of quality filters was applied before subsequent analyses to remove barcodes corresponding to the following categories: (i) low number of total transcripts per nucleus (<1500); (ii) nuclei from possible dying, dead, or stressed cells with high proportions of mitochondrial content setting a threshold on a per sample basis (threshold range: 12 to 18.72%); (iii) outliers in the distribution of number of genes versus number of transcripts by fitting a linear regression model on a per sample basis (p-values between 0.001 and 0.000001); (iv) nuclei with high probability of being doublets using (1) the scDblFinder method on a per sample basis (threshold range: 37.8%–75.7%)

and (2; only in subsequent cell-specific analyses) cluster marker gene expression by removing nuclei of a cluster co-expressing discrepant lineage markers; (v) a cluster of very low gene complexity nuclei with uniformly low numbers of detected genes (threshold in Trailmaker: Log_{10} number of genes <3.16).

For re-analysis of scRNA-seq data from the Steele Cohort, filtered feature barcode-matrices were retrieved as described above and subjected to quality control to remove barcodes corresponding to the following categories: (i) possible dying, dead, or stressed cells with high proportions of mitochondrial content setting a threshold on a per sample basis (threshold range: 7.77 to 50%); (ii) outliers in the distribution of number of genes versus number of transcripts by fitting a linear regression model on a per sample basis (*p*-values between 0.07 and 0.000001); (iii) nuclei with high probability of being doublets using the scDblFinder method on a per sample basis (threshold range: 37.3%–88.8%). For analysis of intratumoral NK cell populations, only NK cells from tumor samples were used.

For analysis of our previously published orthotopic PDAC mouse model scRNA-seq dataset,[43] raw FASTQ data alignment to the mouse reference genome (GRCm39 2024-A), cell filtering, and counting of barcodes and unique molecular identifiers were done using the 10x Genomics Cell Ranger software v9.0.1 with intronic reads included. Filtered barcode-matrices were subjected to quality to remove barcodes corresponding to the following categories: (i) low number of total transcripts per nucleus (<200); (ii) possible dying, dead, or stressed cells with high proportions of mitochondrial content ($\geq$10%); (iii) nuclei with high probability of being doublets using the scDblFinder method on a per sample basis (threshold range: 50.0%–82.7%) and (2; only in subsequent cell-specific analyses) cluster marker gene expression by removing nuclei of a cluster co-expressing discrepant lineage markers.

Data integration, dimensionality reduction, clustering, and cell-type-specific analysis

For our snRNA-seq dataset, we performed data integration on the global dataset using Harmony (HVGs = 7,000), normalization using LogNormalize, and dimensionality reduction with principal component analysis (PCA) selecting for 30 principal components. We excluded ribosomal and mitochondrial gene categories for data integration to avoid bias that can introduce within-cell-type heterogeneity and obscure the differences in expression between cell types. We then performed Uniform Manifold Approximation and Projection (UMAP) embedding to visualize the corresponding Louvain clusters (resolution set to 0.8). To identify the broad cell types, we combined information on previously published marker genes for cell types in human PDAC[22–24] with cluster-specific marker genes from our dataset that were identified by comparing nuclei of each cluster to all other nuclei using the presto package implementation (v.1.0.0) of the Wilcoxon rank-sum test with auROC analysis.

For the Steele Cohort scRNA-seq dataset, we performed integration on the global dataset using Harmony (HVGs = 2,000). Normalization, dimensionality reduction using PCA, UMAP embedding, Louvain clustering, and identification of main cell types was performed as described above.

For the orthotopic PDAC mouse scRNA-seq dataset, we performed integration on the global dataset using Harmony (HVGs = 4,000). Normalization, dimensionality reduction using PCA, UMAP embedding, and identification of main cell types was performed as described above. Cells were clustered using the Leiden clustering (resolution set to 0.8).

For epithelial cell analyses, we extracted and subdivided epithelial cells into subtypes and cellular states using Louvain clustering and marker genes as described in the results, including the following: SPINK1, GP2, CPA1, CPA2, AMY2A, AMY2B, CELA3A (Acinar); SCTR, CFTR, SLC4A4, GLIS3, BICC1 (Ductal); COL1A1, COL1A2, COL3A1, COL4A1, COL6A3, TIMP3, MUC6 (PanIN-like); DLEU1, KRT7, KRT8, KRT19, EPCAM, FXYD3, S100A6 (Cancer); GATA6, SYTL2, CDH1, residual pancreatic (acinar and ductal) lineage markers (classical Cancer); SNAI2, ZEB1, HMGA2, VIM, S100A4, KRT5, MT1E, MT2A, loss of pancreatic lineage markers (basal-like Cancer); MKI67, TOP2A, CIT, CDK1 (Cycling).

For analyses of all other cell types in sn/scRNA-seq datasets, we extracted each cell compartment from the identified major cell types and annotated each cell subtype based on Louvain clustering and marker genes as indicated. For analyses of NK cell subtypes, samples were excluded if $\leq$ 10 total NK cells were retrieved. Marker genes for identifying human $CD56^{dim}$ and $CD56^{bright}$ NK cell subsets based transcriptional signatures in sn/scRNA-seq data were curated from previously published studies.[67,105–107]

Analysis of DEGs between two clusters was performed using the presto package[99] implementation (v.1.0.0) of the Wilcoxon rank-sum test with auROC analysis. Analysis of DEGs in one cluster between two different samples/groups was performed using the pseudobulk approach within the voom pipeline from the limma package (v.3.50.3).[100]

RNA velocity analysis

We inferred transcriptional dynamics and latent time of cell state progression by RNA velocity analysis with scVelo (v.0.3.3)[89] in Python (v3.12.7), according to the developers' tutorial. In brief, BAM files from Cell Ranger outputs were converted to loom files as input for RNA velocity using velocyto (v.0.17.17).[90] For each subset of cells analyzed with RNA velocity, Seurat objects were exported from Trailmaker and converted to anndata objects using Seurat-Disk (v.0.0.0.9021) in R. RNA velocity analysis performed in the dynamical mode on the top 2,500 HVGs with default settings.

Gene set scoring with UCell

To score gene sets on epithelial/cancer cell clusters in the snRNA-seq dataset, we employed UCell[91] (v.2.10.1) according to the developers' tutorial. Gene sets as input for UCell analysis were derived from the Molecular Signatures Database (MSigDB)[104] for MAPK3 (ERK) signaling pathway (C3: Transcription Factor Targets: 'MAPK3 Target Genes') and FGFR signaling pathway (C2:CP:Reactome: 'Reactome_Signaling_by_FGFR'), respectively.

Copy-number variations (CNVs) in epithelial cell types

We inferred large-scale chromosomal CNVs in our snRNA-seq dataset within epithelial cell-derived nuclei using inferCNV (v.1.18.1; https://github.com/broadinstitute/infercnv) with default parameters. The reference gene order file (hg38_gencode_v27) was retrieved from the TrinityCTAT depository (https://data.broadinstitute.org/Trinity/CTAT/cnv/). To enable comparability with the assigned epithelial cell clusters agnostic of patient dependence, we pooled nuclei within one cluster from all samples, and selected acinar, ADM, and ductal_c1 cells as the reference normal set. We selected putatively non-malignant metaplastic ductal_c2 and PanIN-like cells, as well as malignant cancer cell clusters as the observational sets, to confirm absence of large-scale CNVs in non-malignant and presence in malignant cells. All clusters were trimmed to an upper threshold of 10,000 nuclei.

Pathway enrichment analysis in scRNA-/snRNA-seq datasets

To identify significantly enriched pathways in NK cells associated with their cytotoxicity profile in both scRNA-/snRNA-seq datasets, we first identified DEGs between ctx^{hi} NK_C1 and all other clusters as described above. Significantly (adj. p val <0.05) enriched genes were then investigated for pathway enrichment with the Enrichr[92] implementation using the MSigDB Hallmark reference gene set and filtered for gene sets that were (1) found across both datasets, (2) enriched $\geq$ two times (TUM Cohort) and (3) $\geq$ four times (Steele Cohort).

Principal component analyses (PCA) of tumor heterogeneity

To profile (1) intratumoral epithelial subsets based on CISS factor expression, (2) inter- and intratumoral heterogeneity based on epithelial and immune cell subtypes, (3) NK cell subsets based on CD56^{dim} and CD56^{bright} gene expression signatures, (4) and epithelial and NK cell subtypes and respective gene expression in the snRNA-seq dataset, or (5) NK cell clusters in the mouse PDAC scRNA-seq dataset, we performed dimensionality reduction by PCA. For (1), we positioned each cell type by the relative fraction of cells [%] with non-zero expression for each CISS gene, and the overall CISS expression normalized to acinar cells. For (2), we positioned each tumor sample by its cellular composition using the relative fraction [%] of each epithelial and immune cell subtype compared to the total number of cells within the sample, by the fraction of CISS expressing epithelial cells, and by its normalized TIMP-1 expression across all epithelial cells. For (3), we positioned each NK cell cluster by the z-scaled gene expression of CD56^{dim} and CD56^{bright} marker genes. For (4), we positioned each tumor sample by the relative fraction [%] of each cellular subtype (e.g., TIMP-1^{hi} basal-like cancer cells) within the respective compartment (e.g., epithelial cells), the normalized cancer cell TIMP-1 expression, and the norm. NK cell CD74 expression. For (5), we positioned each treatment group by its NK cell composition using the relative fraction [%] of each NK cell cluster compared to the total number of NK cells within that group. For each PCA visualization, the first two principal components were selected.

Inference of cell-cell communication

We inferred cell-cell communication between cancer and NK cells using CellChat,[93] according to the developer's tutorials. We selected 'selected signaling' interactions and updated the ligand-receptor interaction reference database to include TIMP-1/CD74[51,52] and TIMP-1/CD63[59,108] signaling pathways. We used a 'truncated mean' (trim = 0.15) threshold to only identify interactions, if genes are expressed in $\geq$15% of cells.

Cell line transcriptome screening

To screen TIMP-1 mRNA expression levels of human PC cell lines, we re-analyzed data from previously published transcriptome studies.[19,42] We retrieved microarray data from the Moffitt et al. study under GEO accession number GSE71729 and FPKM-normalized RNA-seq data from the Diaferia et al. study under GEO accession number GSE64558. To compare TIMP-1 expression across cell lines from both datasets, we used the MIA PaCa-2 cell line (present in both datasets) as a normalization reference and then applied Z score scaling.

TIMP-1-dependent functions in MIA PaCa-2 cells

To identify TIMP-1-dependent functions in MIA PaCa-2 cells, we performed TIMP-1 knockout using the Mission CRISPR-Cas9 plasmid system (Sigma Aldrich) with two guide RNAs (gRNAs) designed to target different regions of exon 4. Generation of the TIMP-1 knockout cell lines and knockout validation have been described previously.[76] We employed non-manipulated MIA PaCa-2 cells ('TIMP-1 WT'), two validated knockout clones ('TIMP-1 KO 1', 'TIMP-1 KO 2'), and one control line ('CRISPR Control') that acquired only a silent point mutation (g.64C>T) without altering the amino acid sequence, as confirmed by Sanger sequencing as described previously.[76]

To identify TIMP-1-dependent genes in MIA PaCa-2 cells that were independent of the CRISPR-Cas9 gene editing procedure, we performed bulk RNA-seq of all four cell lines (each n = 3). We used DESeq2 (as described above) to identify genes that were differentially expressed in 'TIMP-1 WT' cells compared to both 'TIMP-1 KO 1' and 'TIMP-1 KO 2' cells, but not different between 'TIMP-1 WT' and 'CRISPR Control' cells. Fold-changes of DEGs were then calculated between the mean of pooled TIMP-1-competent ('TIMP-1 WT' and 'CRISPR Control') cells and the mean of pooled TIMP-1-deficient ('TIMP-1 KO 1' and 'TIMP-1 KO 2') cells.

Intracellular TIMP-1 measurement in PDAC cells

Intracellular TIMP-1 levels in MIA PaCa-2 cells were measured using Western Blot or flow cytometry analysis, as indicated. For analysis of TIMP-1 levels after treatment with DMSO (ThermoFisher Scientific) control, Trametinib (Selleckchem), and/or Nintedanib (Sigma-Aldrich) at indicated concentrations, MIA PaCa-2 cells were seeded overnight and then treated as indicated for 6 h (in the presence of Brefeldin A (ThermoFisher Scientific) for the last 4 h). Western blot analysis to determine intracellular TIMP-1 levels was performed as described previously.[76] TIMP-1 levels were detected using sequential primary and secondary antibodies as described previously[76] (primary: rabbit α-human TIMP-1, clone D10E6, #8946, 1:1000, Cell Signaling Technology; secondary: HRP-conjugated goat α-rabbit IgG (H + L), polyclonal, #31462, 1:5000, ThermoFisher Scientific) and normalized to total protein load determined by SYPRO Ruby Protein staining (ThermoFisher Scientific), as described previously.[71] For flow cytometry-based analysis, intracellular TIMP-1 levels were stained using the Two-step Protocol for Fixation/Methanol (ThermoFisher Scientific). In brief, cells were fixed with IC Fixation Buffer (ThermoFisher Scientific) for 30 min at RT, permeabilized by resuspension in ice-cold 100% methanol (Carl Roth), and stored at −20°C overnight. Cells were then washed with FACS buffer and stained for intracellular TIMP-1 using sequential primary and secondary antibodies (primary: rabbit α-human TIMP-1, clone D10E6, #8946, 1:100, Cell Signaling Technology; secondary: AlexaFluor Plus 405-conjugated goat α-rabbit IgG (H + L), polyclonal, 1:100, ThermoFisher Scientific). Each staining step was performed for 60 min at RT, with washes in FACS buffer between steps. For each inhibitor treatment, a respective unstained control was used to detect TIMP-1^{+} cells above background. The resulting values were used to calculate minimal and maximal inhibition of TIMP-1 expression as indicated, as input for calculation of drug synergy (ZIP synergy score[109]) using the online software SynergyFinder+[94] (synergyfinder.org).

Primary human NK cells

We isolated human peripheral blood mononuclear cells (PBMCs) from whole blood samples of healthy donors by density gradient centrifugation using Polymorphprep (Progen), according to the manufacturer's protocol. NK cells were purified from PBMCs by MACS-based negative selection using the MojoSort Human NK Cell Isolation Kit (BioLegend) according to the manufacturer's instructions. NK cells were cultured and stimulated in RPMI 1640 (ThermoFisher Scientific) supplemented with 10% FCS (ThermoFisher Scientific) and 1% P/S (ThermoFisher Scientific). In flow cytometry-based assays employing PBMC-derived NK cells, NK cells were identified as CD56-positive (PE-Cyanine7; mouse α-human, clone QA18A21; 1:100 in FACS buffer; BioLegend *or* Alexa Fluor 488; mouse α-human, clone 5.1H11; 1:100 in FACS buffer; BioLegend) and CD3-negative (eFluor450; mouse α-human, clone OKT3; 1:100 in FACS buffer; ThermoFisher Scientific).

NK cell signaling assays

To stimulate primary human NK cells with TIMP-1 variants, we produced and purified endotoxin-free rhTIMP-1 variants as described previously.[51,59,71] For measurement of granzyme B and perforin expression, NK cells were stimulated for 6 h with rhTIMP-1 (at indicated concentrations); 1 μM Batimastat (Sigma-Aldrich), or respective vehicle controls in the presence (last 4 h) of Brefeldin A (ThermoFisher Scientific). For CD74 interference experiments, NK cells were preincubated with monoclonal α-CD74 antibody milatuzumab (5 μg/mL; ThermoFisher Scientific) or human IgG control antibody (5 μg/mL; ThermoFisher Scientific) for 1 h before rhTIMP-1 stimulations. Intracellular granzyme B and perforin were stained using the Invitrogen Intracellular Fixation & Permeabilization Buffer Set (ThermoFisher Scientific). In brief, NK cells were fixed, incubated for 10 min with Human TruStain FcX (1:20 in FACS buffer; BioLegend) at 4°C to block unspecific Fc receptor binding, permeabilized, and stained for intracellular granzyme B (PE; mouse α-human, clone GB11; 1:50 in permeabilization buffer; ThermoFisher Scientific) or perforin (PE; mouse α-human, clone B-D48; 1:50 in permeabilization buffer; BioLegend) for 2 h at 4°C.

For phospho-signaling assays, NK cells were stimulated with rhTIMP-1, co-cultured with eFluor450-labeled MIA PaCa-2 cell lines (NK 3:1 MIA PaCa-2) in the presence or absence of rhIL-2 (200 IU/mL; BioLegend) (durations indicated), or exposed to cancer-conditioned media derived from unlabeled MIA PaCa-2 cell lines as described below. Intracellular phospho-proteins were stained using the Two-step Protocol for Fixation/Methanol (ThermoFisher Scientific). In brief, cells were fixed with IC Fixation Buffer (ThermoFisher Scientific) for 30 min at RT, transferred to new plates (in case of co-culture; only non-adherent NK cells), resuspended in ice-cold 100% methanol (Carl Roth), and stored at −20°C overnight. NK cells were then washed with FACS buffer, incubated for 10 min with Human TruStain FcX (1:20 in FACS buffer; BioLegend) at 4°C, and stained for intracellular phospho-mTOR (*p*-Ser2448; PE; mouse α-human/mouse, clone MRRBY; 1:50 in FACS buffer; ThermoFisher Scientific), phospho-S6 (*p*-Ser235/236; PerCP-eFluor710; mouse α-human/mouse, clone cupk43k; 1:50 in FACS buffer; ThermoFisher Scientific), or phospho-STAT3 (*p*-Tyr705; FITC; mouse α-human/mouse, clone LUVNKLA; 1:50 in FACS buffer; ThermoFisher Scientific) for 60 min at RT. For co-cultures, NK cells (eFluor450^{neg}) were distinguished from residual co-transferred MIA PaCa-2 cells (eFluor450^{pos}).

For effects of PDAC cell line-conditioned media on NK cell phenotypes, unlabeled MIA PaCa-2 cell lines were cultivated for 48 h without NK cells and conditioned supernatants were harvested and employed for experiments or stored at – 80°C until further use. Primary NK cells were then cultivated in cancer-conditioned media or fresh control media for indicated durations, in the presence or absence of rhIL-2 (200 IU/mL; BioLegend), and stained as described below. For measurement of intracellular IFN-γ (FITC; mouse α-human, clone 4S.B3; 1:50 in permeabilization buffer; ThermoFisher Scientific) and TNF-α levels (PE; mouse α-human, clone Mab11; 1:50 in permeabilization buffer; BD Pharmingen), Brefeldin A (ThermoFisher Scientific) was added for the last 4 h of incubation, and staining was performed as described above (for granzyme B and perforin). For measurement of neutral lipid content, NK

cells were washed, and stained with HCS LipidTOX Red Neutral Lipid Stain (1:1000 in FACS buffer; ThermoFisher Scientific) for 30 min at RT.

Flow cytometry was performed using an SA3800 Spectral Analyzer (Sony Biotechnologies) or SP6800 Spectral Analyzer (Sony Biotechnologies) and data was analyzed using the FlowJo software (v.10.10.0, BD Biosciences). The relative fraction [%] of *p*-mTOR/p-S6-active NK cells was calculated as follows:

$$1 - \text{p-mTOR}^{neg}/\text{p-S6}^{neg}\ \text{NK cells}$$

NK cell cytotoxicity assay

To determine the suppressive activity of MIA PaCa-2 cell lines on NK cell cytotoxicity, NK cells were either exposed to MIA PaCa-2 cell line-conditioned media or fresh control media (as described above) for 24 h, or co-cultured with MIA PaCa-2 cell lines (NK 3:1 MIA PaCa-2) in the presence or absence of rhIL-2 (200 IU/mL; BioLegend) for 24 h. NK cells were then exposed to the eFluor450-labeled NK-sensitive K562 target cell line at indicated effector-to-target ratios for 3 h. Killing activity of NK cells was analyzed by flow cytometry. Cells were incubated for 10 min with Human TruStain FcX (1:20 in FACS buffer; BioLegend) at 4°C and then stained for NK cell marker CD56 (PE-Cyanine7; mouse α-human, clone QA18A21; 1:100 in FACS buffer; BioLegend) and degranulation marker CD107a (PerCP-Cyanine5.5; mouse α-human, clone H4A3; 1:100 in FACS buffer; BioLegend) for 15 min at 4°C. To assess K562 cell death, cells were washed, stained with Annexin V (FITC; 1:40 in Annexin V Binding Buffer; BioLegend) for 15 min at RT, and resuspended in Annexin V binding buffer containing 7-AAD (1:20; ThermoFisher Scientific) for flow cytometry using an SA3800 Spectral Analyzer (Sony Biotechnologies). Data was analyzed using the FlowJo software (v.10.10.0, BD Biosciences). NK cell-specific induction of K562 cell apoptosis was determined by correcting for background apoptosis of K562 cells cultured without NK cells. 7-AAD^{pos} dead NK cells were excluded from the analysis.

QUANTIFICATION AND STATISTICAL ANALYSIS

Statistical analyses

Sample sizes are indicated for human specimens. Tumor samples from the TUM Cohort were allocated to experimental groups based on tumor grade. No statistical method was used to predetermine sample sizes of experimental groups. If possible, experiments were randomized and investigators were blinded to experimental allocation and outcome assessment. The GraphPad Prism software (v.10.4.1) or R (v.4.4.2) were used for statistical analyses, unless otherwise indicated. Affinity Designer (v.1.10.8). was used for graphical data visualization. Statistical analyses were performed in a two-tailed manner. Normal distribution of experimental groups was tested by Shapiro-Wilk tests. Parametric and nonparametric (in the absence of normal distribution) statistical tests were used to assess statistical significance, and corrected for multiple hypothesis testing if applicable, as indicated in the figure legends. The significance level was set to 0.05. Data are shown as mean ± s.d., mean ±95% CI, box and whiskers plots, or violin plots, as indicated in the figure legends.

Cell Reports Medicine, Volume *7*

Supplemental information

Multimodal profiling of pancreatic cancer reveals a TIMP-1-dominated secretory profile determining pro-tumor immunoinstruction in human cancers

Julian Frädrich, Carmen Mota Reyes, Michel Hendel, Vanessa Brunner, Batu Toledo, Damjan Manevski, Alexander Sommer, Daniel Häußler, Dominik Beck, Daniele Lucarelli, Jaime Martínez de Villareal, Lennard Halle, Raphael Kfuri-Rubens, Kaan Çifcibaşı, Anna Hirschberger, Rupert Öllinger, Percy A. Knolle, Katja Steiger, Roland Rad, Fabian J. Theis, Francisco X. Real, Stefanie Bärthel, Jan P. Böttcher, Dieter Saur, Ihsan Ekin Demir, and Achim Krüger

Fig. S1

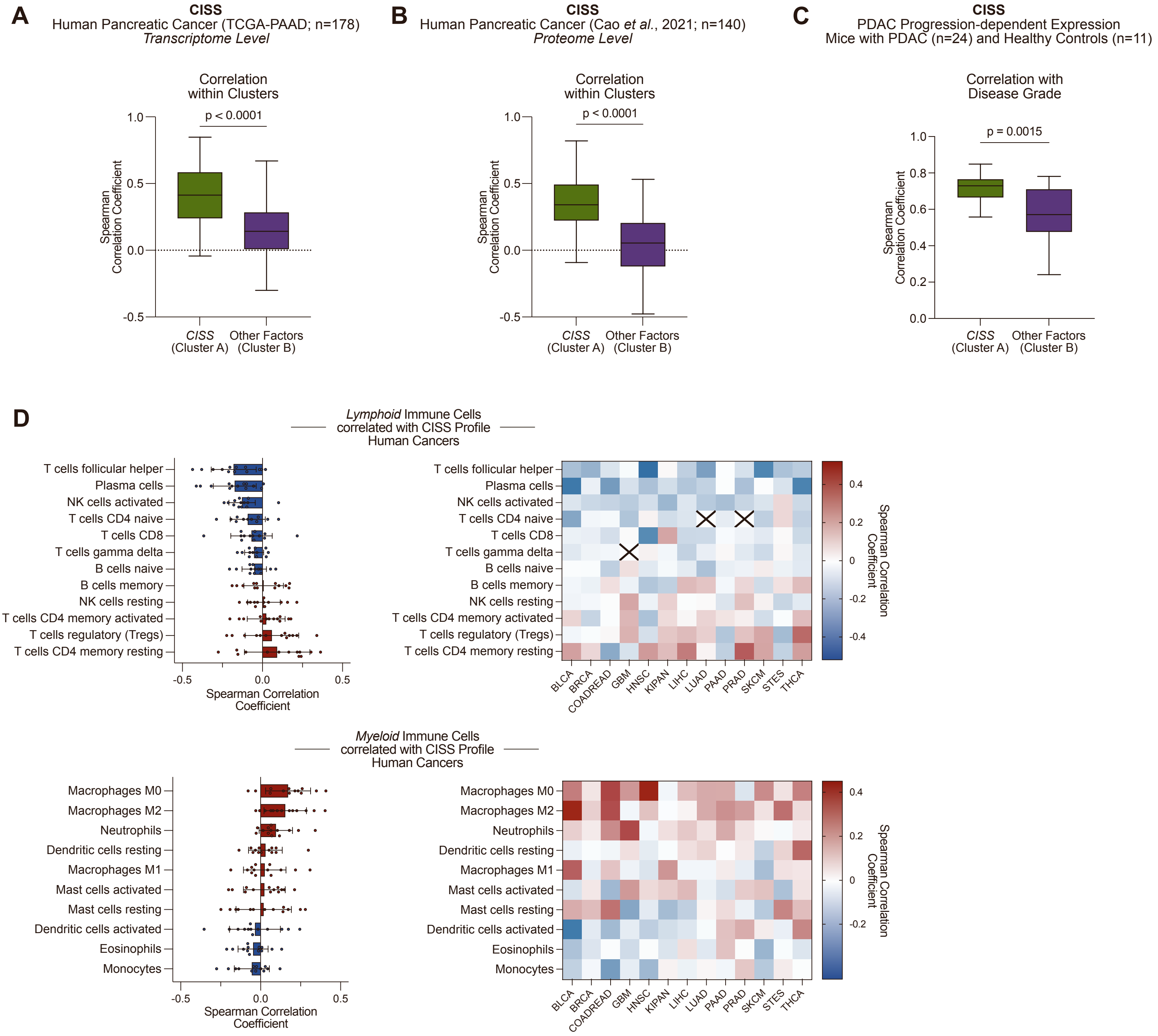
A
CISS
Human Pancreatic Cancer (TCGA-PAAD; n=178)
Transcriptome Level
Correlation within Clusters
p < 0.0001
Spearman Correlation Coefficient
CISS (Cluster A)
Other Factors (Cluster B)
B
CISS
Human Pancreatic Cancer (Cao et al., 2021; n=140)
Proteome Level
Correlation within Clusters
p < 0.0001
Spearman Correlation Coefficient
CISS (Cluster A)
Other Factors (Cluster B)
C
CISS
PDAC Progression-dependent Expression
Mice with PDAC (n=24) and Healthy Controls (n=11)
Correlation with Disease Grade
p = 0.0015
Spearman Correlation Coefficient
CISS (Cluster A)
Other Factors (Cluster B)
D
Lymphoid Immune Cells correlated with CISS Profile Human Cancers
T cells follicular helper
Plasma cells
NK cells activated
T cells CD4 naive
T cells CD8
T cells gamma delta
B cells naive
B cells memory
NK cells resting
T cells CD4 memory activated
T cells regulatory (Tregs)
T cells CD4 memory resting
Spearman Correlation Coefficient
BLCA
BRCA
COADREAD
GBM
HNSC
KIPAN
LIHC
LUAD
PAAD
PRAD
SKCM
STES
THCA
Myeloid Immune Cells correlated with CISS Profile Human Cancers
Macrophages M0
Macrophages M2
Neutrophils
Dendritic cells resting
Macrophages M1
Mast cells activated
Mast cells resting
Dendritic cells activated
Eosinophils
Monocytes
Spearman Correlation Coefficient

Fig. S1 Statistical evaluation of CISS in human PDAC and its correlation with PDAC progression in mice, related to Fig. 2. (A) Statistical comparison of Spearman correlation factors between CISS (Cluster A) and the other 17 factors (Cluster B) from TCGA-PAAD RNA-seq data (see *Fig. 2A*). Statistical comparison was performed using Mann-Whitney test. **(B)** Statistical comparison of Spearman correlation factors between CISS (Cluster A) and the other 17 factors (Cluster B) from PDAC tumor proteomics data (Cao cohort[S1]; see *Fig. 2B*). Statistical comparison was performed using Mann-Whitney test. **(C)** The 36 secreted factors (including the 19 CISS factors) in murine KPC tumors and control pancreata from Fig. 1 were correlated with disease grades (replaced by numerical values: 0 = control pancreas; 1 = early PDAC; 2 = grade 2; 3 = grade 3; 4 = grade 4) using Spearman correlation. For statistics, Spearman correlation coefficients were compared between CISS factors and the other 17 factors using unpaired Student's t test. Data represented as box and whiskers plots. **(D, Left)** Spearman correlation coefficient for each immune cell subtype in all entities (dots), separated by lymphoid and myeloid cells. Data showing all individual points. **(D, Right)** Heatmap visualization of left panels across human cancer entities. X indicates cell type was not detected. Also see *Fig. 2I.*

Fig. S2

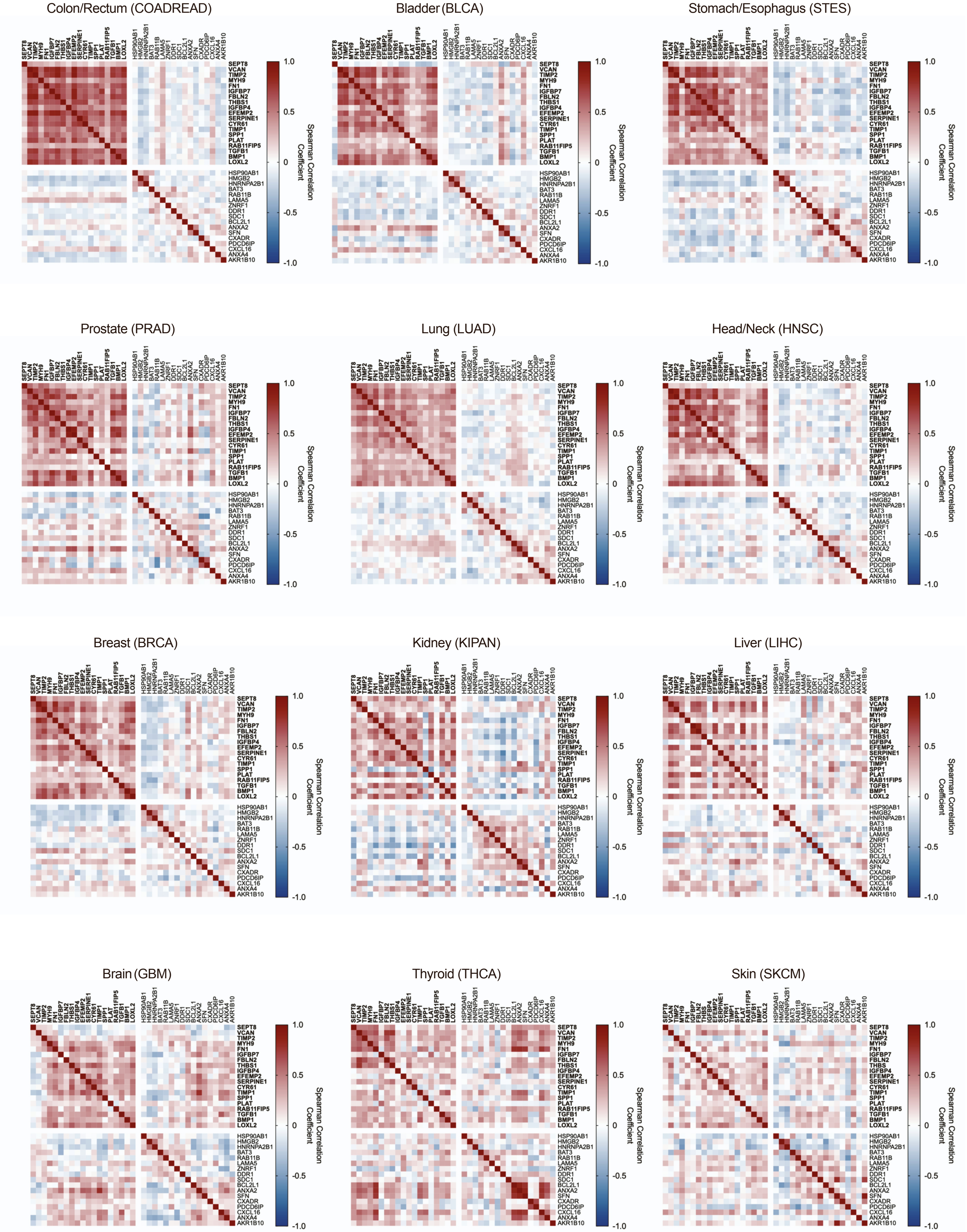

Colon/Rectum (COADREAD)
Bladder (BLCA)
Stomach/Esophagus (STES)
Prostate (PRAD)
Lung (LUAD)
Head/Neck (HNSC)
Breast (BRCA)
Kidney (KIPAN)
Liver (LIHC)
Brain (GBM)
Thyroid (THCA)
Skin (SKCM)
Spearman Correlation Coefficient
1.0
0.5
0
-0.5
-1.0
SEPT8
VCAN
TIMP2
MYH9
FN1
IGFBP7
FBLN2
THBS1
IGFBP4
EFEMP2
SERPINE1
CYR61
TIMP1
SPP1
PLAT
RAB11FIP5
TGFB1
BMP1
LOXL2
HSP90AB1
HMGB2
HNRNPA2B1
BAT3
RAB11B
LAMA5
ZNRF1
DDR1
SDC1
BCL2L1
ANXA2
SFN
CXADR
PDCD6IP
CXCL16
ANXA4
AKR1B10

Fig. S2 Pan-Cancer validation of CISS across human solid cancers, related to Fig. 2. Correlation analysis of mRNA expression levels of the 36 secreted factors (see *Fig. 2A*) in bulk tumor RNA-seq data from TCGA cohorts (see *Fig. 2G,H*). Order of genes (including the CISS factors in the upper left cluster) are aligned with the TCGA-PAAD reference cohort (see *Fig. 2A*). BLCA, bladder urothelial carcinoma (n = 408); BRCA, breast invasive carcinoma (n = 1,093); COADREAD, colorectal adenocarcinoma (n = 379); GBM, glioblastoma multiforme (n = 152); HNSC, head and neck squamous cell carcinoma (n = 520); KIPAN, Pan-kidney cancer cohort (n = 889); LIHC, liver hepatocellular carcinoma (n = 371); LUAD, lung adenocarcinoma (n = 515); PAAD, pancreatic adenocarcinoma (n = 178); PRAD, prostate adenocarcinoma (n = 497); SKCM, skin cutaneous melanoma (n = 103); STES, stomach and esophageal carcinoma (n = 599); THCA, thyroid carcinoma (n = 501).

Fig. S3

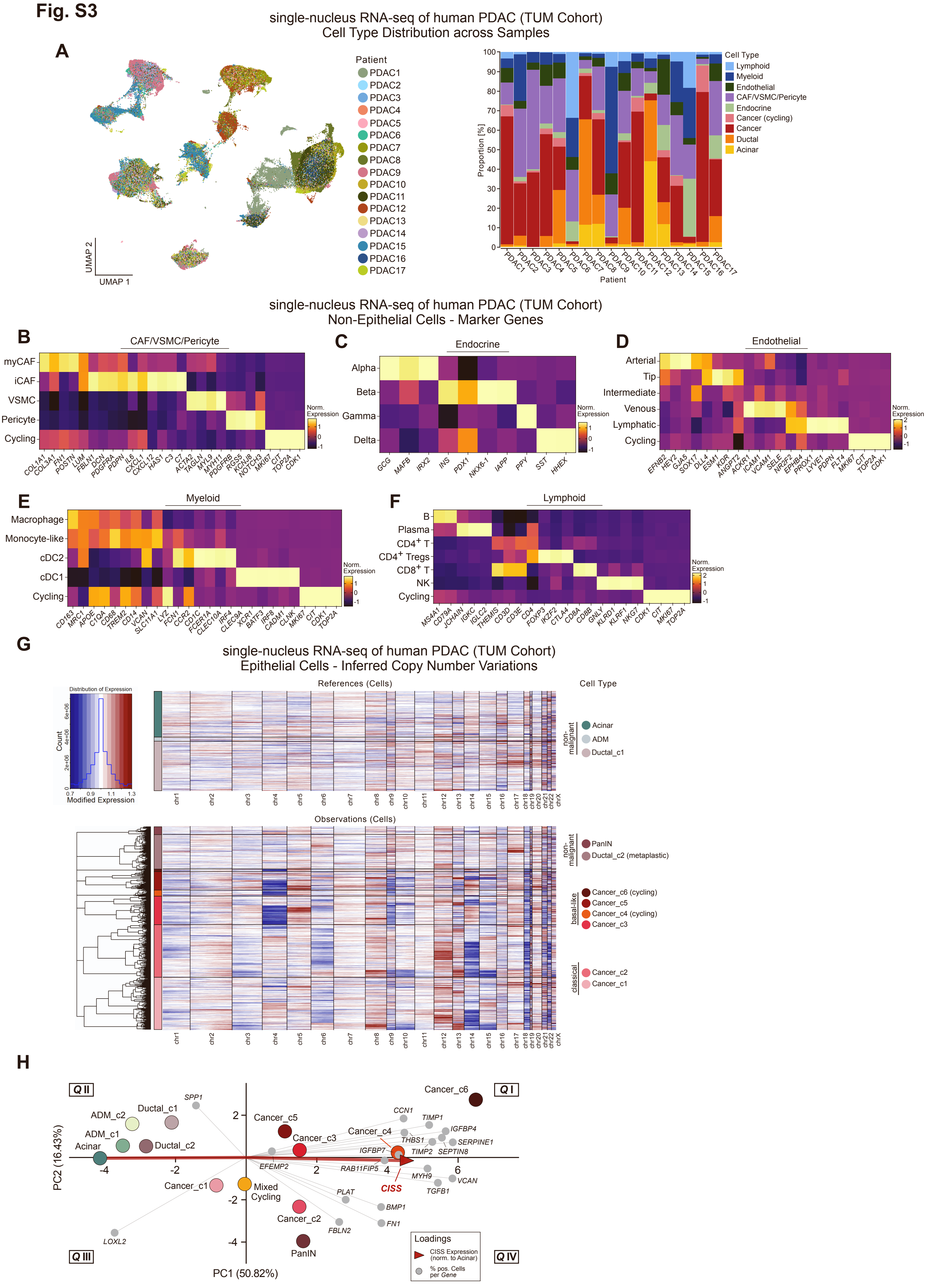
single-nucleus RNA-seq of human PDAC (TUM Cohort)
Cell Type Distribution across Samples
A
UMAP 1
UMAP 2
Patient
PDAC1
PDAC2
PDAC3
PDAC4
PDAC5
PDAC6
PDAC7
PDAC8
PDAC9
PDAC10
PDAC11
PDAC12
PDAC13
PDAC14
PDAC15
PDAC16
PDAC17
Proportion [%]
Cell Type
Lymphoid
Myeloid
Endothelial
CAF/VSMC/Pericyte
Endocrine
Cancer (cycling)
Cancer
Ductal
Acinar
single-nucleus RNA-seq of human PDAC (TUM Cohort)
Non-Epithelial Cells - Marker Genes
B
CAF/VSMC/Pericyte
myCAF
iCAF
VSMC
Pericyte
Cycling
COL1A1 COL3A1 FN1 POSTN LUM FBLN1 DCN PDGFRA PDPN IL6 CXCL1 CXCL12 HAS1 C3 C7 ACTA2 TAGLN MYL9 MYH11 PDGFRB RGS5 KCNJ8 NOTCH3 MKI67 CIT TOP2A CDK1
Norm. Expression
C
Endocrine
Alpha
Beta
Gamma
Delta
GCG MAFB IRX2 INS PDX1 NKX6-1 IAPP PPY SST HHEX
D
Endothelial
Arterial
Tip
Intermediate
Venous
Lymphatic
Cycling
EFNB2 HEY2 GJA5 SOX17 DLL4 ESM1 KDR ANGPT2 ACKR1 ICAM1 VCAM1 SELE NR2F2 EPHB4 PROX1 LYVE1 PDPN FLT4 MKI67 CIT TOP2A CDK1
E
Myeloid
Macrophage
Monocyte-like
cDC2
cDC1
Cycling
CD163 MRC1 APOE C1QA CD68 TREM2 CD14 VCAN SLC11A1 LYZ FCN1 CCR2 CD1C FCER1A CLEC10A IRF4 CLEC9A XCR1 BATF3 IRF8 CADM1 CLNK MKI67 CIT CDK1 TOP2A
F
Lymphoid
B
Plasma
CD4+ T
CD4+ Tregs
CD8+ T
NK
Cycling
MS4A1 CD79A JCHAIN IGKC IGLC2 THEMIS CD3D CD3E CD4 FOXP3 IKZF2 CTLA4 CD8A CD8B GNLY KLRD1 KLRF1 NKG7 CDK1 CIT MKI67 TOP2A
G
single-nucleus RNA-seq of human PDAC (TUM Cohort)
Epithelial Cells - Inferred Copy Number Variations
Distribution of Expression
Count
Modified Expression
References (Cells)
Cell Type
non-malignant
Acinar
ADM
Ductal_c1
Observations (Cells)
PanIN
Ductal_c2 (metaplastic)
basal-like
Cancer_c6 (cycling)
Cancer_c5
Cancer_c4 (cycling)
Cancer_c3
classical
Cancer_c2
Cancer_c1
chr1 chr2 chr3 chr4 chr5 chr6 chr7 chr8 chr9 chr10 chr11 chr12 chr13 chr14 chr15 chr16 chr17 chr18 chr19 chr20 chr21 chr22 chrX
H
Q II
Q I
Q III
Q IV
PC1 (50.82%)
PC2 (16.43%)
Acinar
ADM_c1
ADM_c2
Ductal_c1
Ductal_c2
Cancer_c1
Mixed Cycling
Cancer_c2
PanIN
Cancer_c3
Cancer_c4
Cancer_c5
Cancer_c6
SPP1
LOXL2
CCN1
TIMP1
IGFBP4
THBS1
SERPINE1
IGFBP7
TIMP2
SEPTIN8
EFEMP2
RAB11FIP5
MYH9
VCAN
TGFB1
CISS
PLAT
BMP1
FN1
FBLN2
Loadings
CISS Expression (norm. to Acinar)
% pos. Cells per Gene

Fig. S3 Cell types, inferred CNVs, and CISS expression across PDAC patient tumors, related to Fig. 3. (A) (Left) UMAP embedding of single-nucleus profiles (dots) derived from snRNA-seq of treatment-naïve PDAC patient tumors (n = 17) colored by patients (color legend, right). Post-hoc cell-type annotations are shown in *Fig. 3A*. (Right) Cell type distributions shown by proportions (y axis) of each cell type (color legend, right) across patient tumor samples (columns, x axis). **(B-F)** snRNA-seq captured heterogenous stromal (B), endocrine (C), endothelial (D), myeloid immune (E), and lymphoid immune (F) cell subsets in PDAC patient tumors (see *Fig. 3C*). Heatmap visualization of selected marker gene expression (z-scores) across identified cell subsets. **(G)** Inferred copy number variations (CNVs) by inferCNV analysis of epithelial subsets as annotated in *Fig. 3D,E*. Inferred amplifications (red) and deletions (blue) based on gene expression (color bar) are shown in each chromosomal locus (columns) from each cell (rows), clustered by annotated epithelial cell types (color legend, right). Putatively malignant, Ductal_c2, PanIN-like cells were used as observational groups (lower panel) and compared to Acinar, ADM, Ductal_c1 cells as reference cells (upper panel). **(H)** Principal component analysis (PCA) of intratumoral epithelial subsets (see *Fig. 3D-H*), based on total CISS (red triangle) and individual CISS factors (grey dots). PC scores of each cell subtype (colored dots) are indicated.

Fig. S4

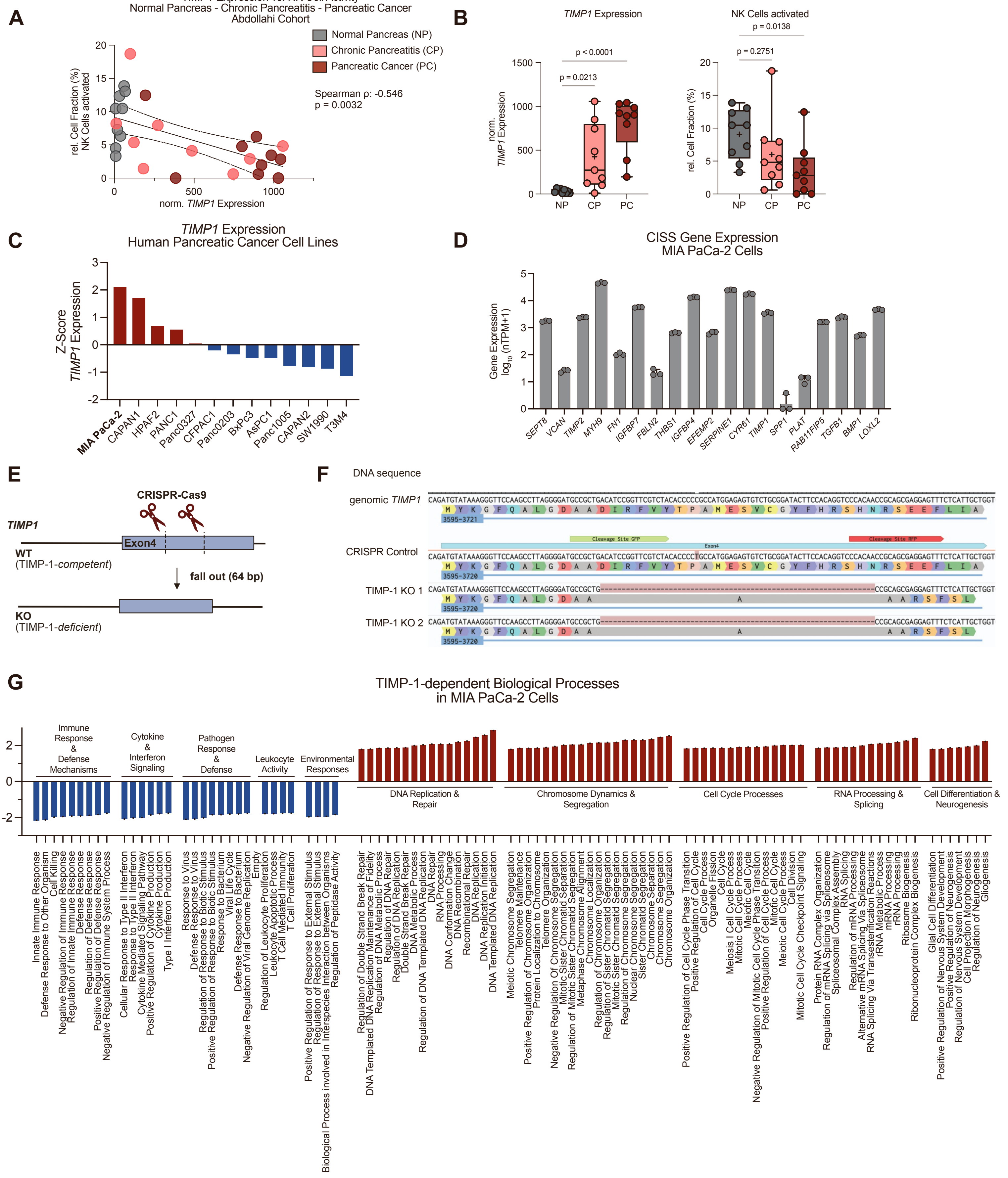
A
TIMP1 Expression vs. NK Cell Activity
Normal Pancreas - Chronic Pancreatitis - Pancreatic Cancer
Abdollahi Cohort
Normal Pancreas (NP)
Chronic Pancreatitis (CP)
Pancreatic Cancer (PC)
Spearman ρ: -0.546
p = 0.0032
rel. Cell Fraction (%) NK Cells activated
norm. TIMP1 Expression
B
TIMP1 Expression
NK Cells activated
p < 0.0001
p = 0.0213
p = 0.0138
p = 0.2751
norm. TIMP1 Expression
rel. Cell Fraction (%)
NP
CP
PC
C
TIMP1 Expression
Human Pancreatic Cancer Cell Lines
Z-Score TIMP1 Expression
MIA PaCa-2
CAPAN1
HPAF2
PANC1
Panc0327
CFPAC1
Panc0203
BxPc3
AsPC1
Panc1005
CAPAN2
SW1990
T3M4
D
CISS Gene Expression
MIA PaCa-2 Cells
Gene Expression log10 (nTPM+1)
SEPT8
VCAN
TIMP2
MYH9
FN1
IGFBP7
FBLN2
THBS1
IGFBP4
EFEMP2
SERPINE1
CYR61
TIMP1
SPP1
PLAT
RAB11FIP5
TGFB1
BMP1
LOXL2
E
CRISPR-Cas9
TIMP1
WT
(TIMP-1-competent)
Exon4
fall out (64 bp)
KO
(TIMP-1-deficient)
F
DNA sequence
genomic TIMP1
CRISPR Control
TIMP-1 KO 1
TIMP-1 KO 2
Cleavage Site GFP
Cleavage Site RFP
Exon4
3595-3721
3595-3720
G
TIMP-1-dependent Biological Processes
in MIA PaCa-2 Cells
NES
Immune Response & Defense Mechanisms
Cytokine & Interferon Signaling
Pathogen Response & Defense
Leukocyte Activity
Environmental Responses
DNA Replication & Repair
Chromosome Dynamics & Segregation
Cell Cycle Processes
RNA Processing & Splicing
Cell Differentiation & Neurogenesis
Innate Immune Response
Defense Response to Other Organism
Cell Killing
Negative Regulation of Immune Response
Regulation of Innate Immune Response
Defense Response
Positive Regulation of Defense Response
Regulation of Defense Response
Negative Regulation of Immune System Process
Cellular Response to Type II Interferon
Response to Type II Interferon
Cytokine Mediated Signaling Pathway
Positive Regulation of Cytokine Production
Cytokine Production
Type I Interferon Production
Response to Virus
Defense Response to Virus
Regulation of Response to Biotic Stimulus
Positive Regulation of Response to Biotic Stimulus
Response to Bacterium
Viral Life Cycle
Defense Response to Bacterium
Negative Regulation of Viral Genome Replication
Empty
Regulation of Leukocyte Proliferation
Leukocyte Apoptotic Process
T Cell Mediated Immunity
T Cell Proliferation
Positive Regulation of Response to External Stimulus
Regulation of Response to External Stimulus
Biological Process involved in Interspecies Interaction between Organisms
Regulation of Peptidase Activity
Regulation of Double Strand Break Repair
DNA Templated DNA Replication Maintenance of Fidelity
Regulation of DNA Metabolic Process
Regulation of DNA Repair
Regulation of DNA Replication
Regulation of Double Strand Break Repair
Double Strand Break Repair
DNA Metabolic Process
Regulation of DNA Templated DNA Replication
DNA Repair
RNA Processing
DNA Conformation Change
DNA Recombination
Recombinational Repair
DNA Replication
DNA Replication Initiation
DNA Templated DNA Replication
Meiotic Chromosome Segregation
Telomere Maintenance
Positive Regulation of Chromosome Organization
Protein Localization to Chromosome
Telomere Organization
Negative Regulation Of Chromosome Segregation
Mitotic Sister Chromatid Separation
Regulation of Mitotic Sister Chromatid Segregation
Metaphase Chromosome Alignment
Chromosome Localization
Regulation of Chromosome Organization
Regulation of Sister Chromatid Segregation
Mitotic Sister Chromatid Segregation
Regulation of Chromosome Segregation
Nuclear Chromosome Segregation
Sister Chromatid Segregation
Chromosome Segregation
Chromosome Organization
Positive Regulation of Cell Cycle Phase Transition
Positive Regulation of Cell Cycle
Cell Cycle Process
Organelle Fission
Cell Cycle
Meiosis I Cell Cycle Process
Mitotic Cell Cycle Process
Meiotic Cell Cycle
Negative Regulation of Mitotic Cell Cycle Phase Transition
Positive Regulation of Cell Cycle Process
Mitotic Cell Cycle
Meiotic Cell Cycle Process
Cell Division
Mitotic Cell Cycle Checkpoint Signaling
Protein RNA Complex Organization
Regulation of mRNA Splicing via Spliceosome
Spliceosomal Complex Assembly
RNA Splicing
Regulation of mRNA Processing
Alternative mRNA Splicing Via Spliceosome
RNA Splicing Via Transesterification Reactions
rRNA Metabolic Process
mRNA Processing
rRNA Processing
Ribosome Biogenesis
Ribonucleoprotein Complex Biogenesis
Glial Cell Differentiation
Positive Regulation of Nervous System Development
Regulation of Neurogenesis
Positive Regulation of Nervous System Development
Regulation of Nervous System Development
Cell Projection Morphogenesis
Regulation of Neurogenesis
Gliogenesis

Fig. S4 Functional role of TIMP-1 in TIMP-1^{hi} basal-like MIA PaCa-2 PC cell line, related to Fig. 4. **(A)** Validation of the correlation between *TIMP1* expression and reduced NK cell activity (see *Fig. 4D-F*) in an external patient cohort (Abdollahi *et al.*[S2]) comprising normal pancreas controls (n = 9), chronic pancreatitis (n = 9), and PC patients (n = 9). **(B)** *TIMP1* expression (left) and CIBERSORTx-determined NK cell activity (right) from (A). Significance was calculated by Kruskal-Wallis test and Dunn's test for multiple comparisons correction. **(C)** Screening of *TIMP1* expression (z-scores) data from two previously published RNA-seq datasets of PC cell lines (Moffitt *et al.*[S3], Diaferia *et al.*[S4]) revealed mesenchymal/basal-like MIA PaCa-2 cell line as model system for TIMP-1^{hi} basal-like cancer cells. **(D)** RNA-seq (see *Fig. 4J*) showed expression of all CISS factors in MIA PaCa-2 cell line. **(E)** Graphical scheme of *TIMP1* sites targeted by single-guide RNAs to generate MIA PaCa-2 TIMP-1 KO cells. bp; base pairs. **(F)** Sanger sequencing for validation of CRISPR-Cas9-induced frame-shift mutation in TIMP-1 KO 1 and KO 2 cells and absence of altered amino acid sequence in CRISPR Control cells (silent point mutation g.64C>T). **(G)** TIMP-1-dependent biological processes in MIA PaCa-2 cells revealed by GSEA (see *Fig. 4M*). NES, normalized enrichment score. Data in (A,B,D) showing all individual points and represented as z-score bars (C), mean ± s.d. (D), and NES bars (G).

Fig. S5

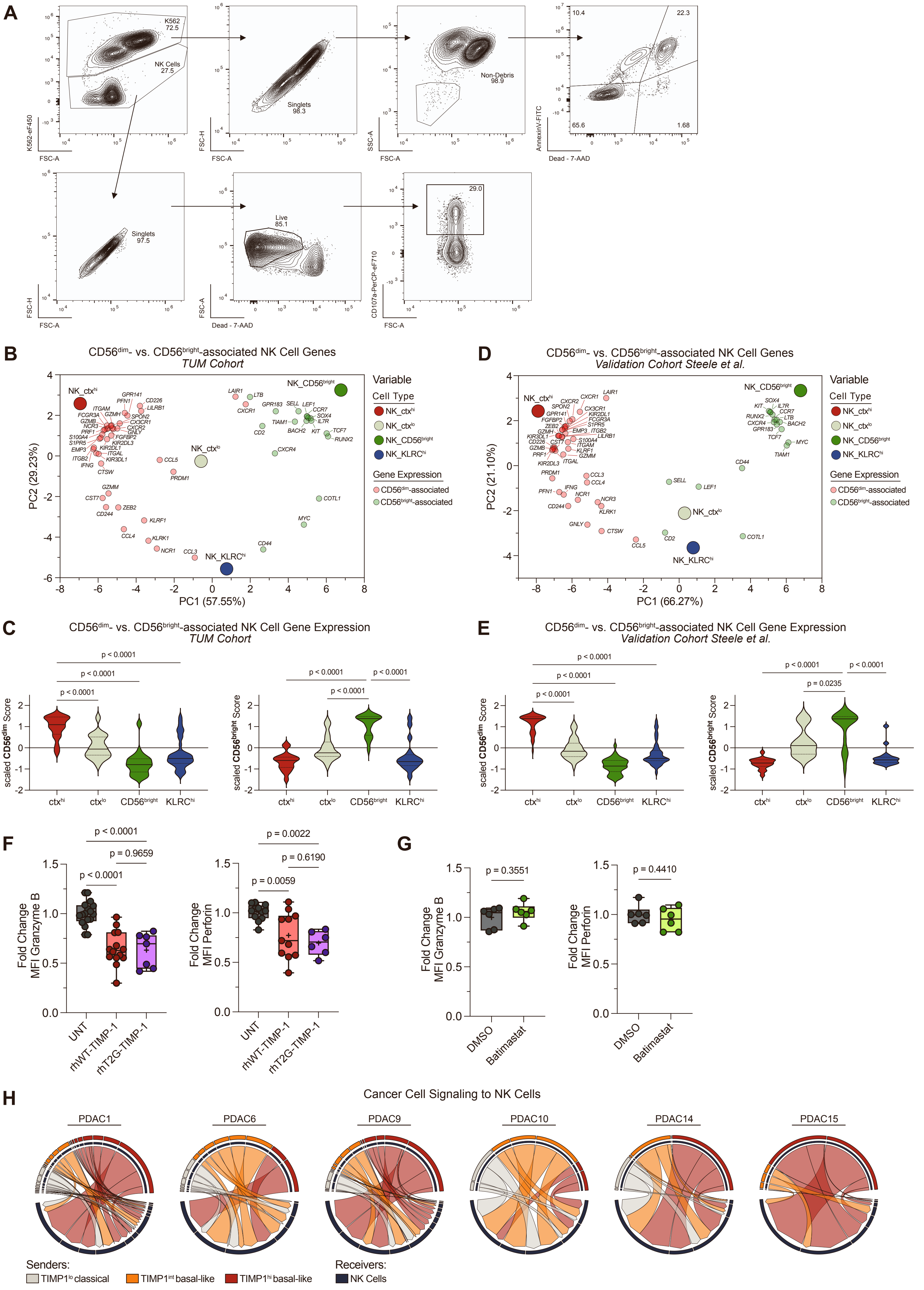
A
K562 72.5
NK Cells 27.5
Singlets 98.3
Non-Debris 98.9
10.4
22.3
65.6
1.68
Singlets 97.5
Live 85.1
29.0
K562-eF450
FSC-A
FSC-H
SSC-A
AnnexinV-FITC
Dead - 7-AAD
CD107a-PerCP-eF710
B
CD56dim- vs. CD56bright-associated NK Cell Genes
TUM Cohort
PC1 (57.55%)
PC2 (29.23%)
NK_ctxhi
NK_ctxlo
NK_CD56bright
NK_KLRChi
Variable
Cell Type
Gene Expression
CD56dim-associated
CD56bright-associated
C
CD56dim- vs. CD56bright-associated NK Cell Gene Expression
TUM Cohort
scaled CD56dim Score
scaled CD56bright Score
ctxhi
ctxlo
CD56bright
KLRChi
p < 0.0001
D
CD56dim- vs. CD56bright-associated NK Cell Genes
Validation Cohort Steele et al.
PC1 (66.27%)
PC2 (21.10%)
E
CD56dim- vs. CD56bright-associated NK Cell Gene Expression
Validation Cohort Steele et al.
p = 0.0235
F
Fold Change MFI Granzyme B
Fold Change MFI Perforin
p < 0.0001
p = 0.9659
p = 0.0022
p = 0.6190
p = 0.0059
UNT
rhWT-TIMP-1
rhT2G-TIMP-1
G
p = 0.3551
p = 0.4410
DMSO
Batimastat
H
Cancer Cell Signaling to NK Cells
PDAC1
PDAC6
PDAC9
PDAC10
PDAC14
PDAC15
Senders:
TIMP1lo classical
TIMP1int basal-like
TIMP1hi basal-like
Receivers:
NK Cells

Fig. S5 TIMP-1-dependent regulation of NK cell cytotoxic capacity, related to Fig. 4,5,6. (A) Representative flow cytometry plots showing the gating strategy for NK cell cytotoxicity assays (see *Fig. 4*, *Fig. 6*). **(B-E)** Classification of intratumoral NK cell clusters based on $CD56^{dim}$ and $CD56^{bright}$ NK cell gene expression signatures in our cohort (B,C) or Steele cohort[S5] (D,E) (see Fig. *5A-F*). $CD56^{dim}$ and $CD56^{bright}$ NK cell marker genes were curated from previously published literature[S6,S7,S8,S9], and their expression was associated with intratumoral NK cell clusters (see Fig. 5B,D) using PCA for individual genes (B,D) or as grouped expression scores (C,E). Significance (C,E) was calculated using one-way ANOVA for matched data (genes) and Dunnett test for multiple comparisons correction between indicated groups. **(F)** NK cell granzyme B and perforin expression upon exposure to 500 ng/mL recombinant human (rh) WT-TIMP-1 vs. 500 ng/mL T2G-TIMP-1 (granzyme B: UNT, n = 17; WT-TIMP-1, n = 14; T2G-TIMP-1, n = 7; perforin: UNT, n = 12; WT-TIMP-1, n = 11; T2G-TIMP-1, n = 6). UNT and WT-TIMP-1 shared with *Fig. 5G*. **(G)** NK cell granzyme B and perforin expression upon exposure to batimastat or DMSO control (granzyme B and perforin: DMSO, n = 6; Batimastat, n = 6). Significance was calculated by (F) one-way ANOVA and Tukey test for multiple comparisons correction or (G) unpaired Student's t test. **(H)** Inferred cancer cell-to-NK cell signaling using CellChat individually for each patient (also see *Fig. 5N*). Data represented as violin plots (C,E) or showing biological replicates from 3 independent experiments and represented as box and whiskers plots (F,G).

Fig. S6

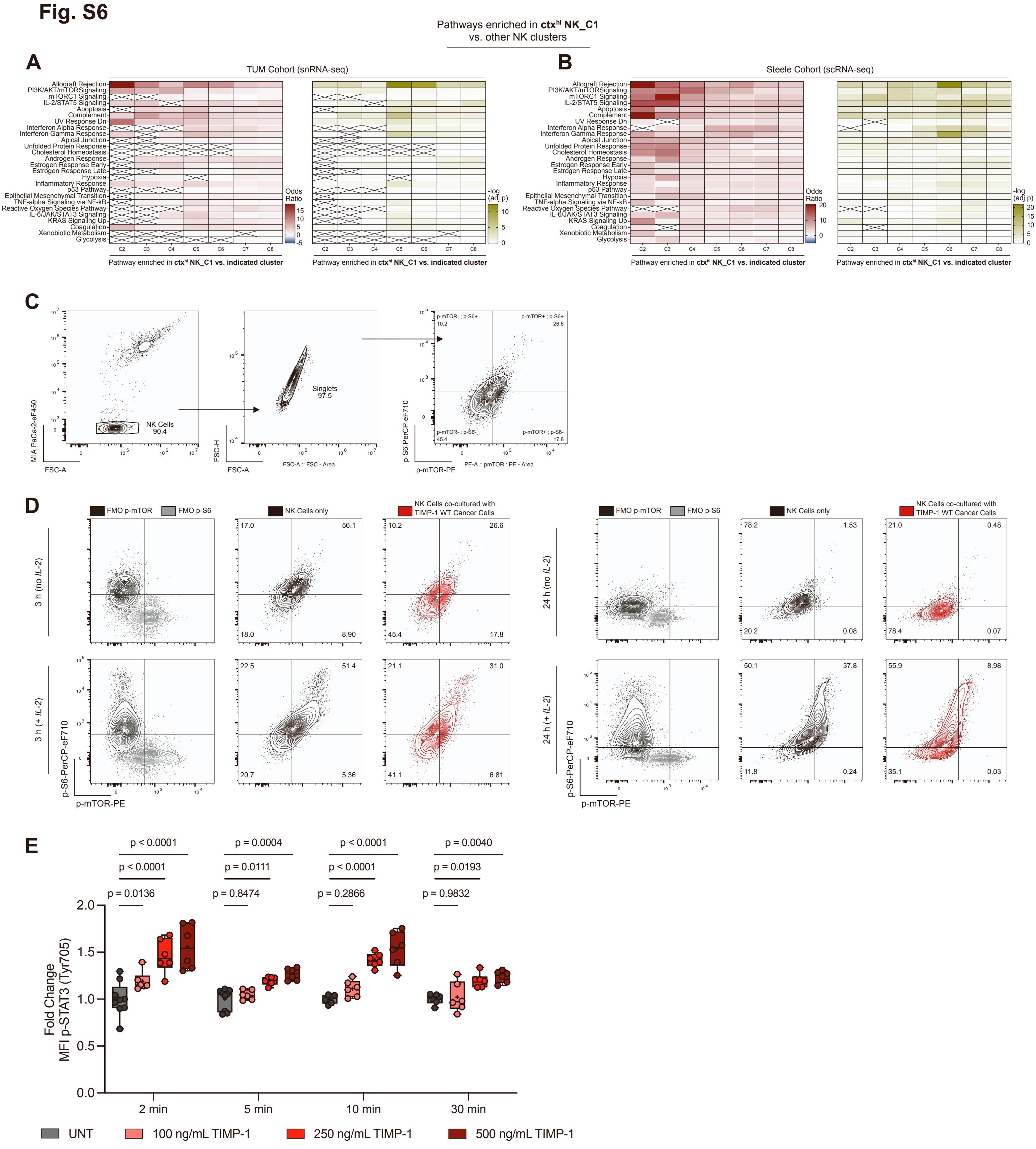
Pathways enriched in ctxhi NK_C1 vs. other NK clusters
A
TUM Cohort (snRNA-seq)
B
Steele Cohort (scRNA-seq)
Allograft Rejection
PI3K/AKT/mTORSignaling
mTORC1 Signaling
IL-2/STAT5 Signaling
Apoptosis
Complement
UV Response Dn
Interferon Alpha Response
Interferon Gamma Response
Apical Junction
Unfolded Protein Response
Cholesterol Homeostasis
Androgen Response
Estrogen Response Early
Estrogen Response Late
Hypoxia
Inflammatory Response
p53 Pathway
Epithelial Mesenchymal Transition
TNF-alpha Signaling via NF-kB
Reactive Oxygen Species Pathway
IL-6/JAK/STAT3 Signaling
KRAS Signaling Up
Coagulation
Xenobiotic Metabolism
Glycolysis
Odds Ratio
-log (adj p)
C2 C3 C4 C5 C6 C7 C8
Pathway enriched in ctxhi NK_C1 vs. indicated cluster
C
NK Cells 90.4
Singlets 97.5
p-mTOR- ; p-S6+ 10.2
p-mTOR+ ; p-S6+ 26.6
p-mTOR- ; p-S6- 45.4
p-mTOR+ ; p-S6- 17.8
MIA PaCa-2-eF450
FSC-A
FSC-H
FSC-A :: FSC - Area
p-S6-PerCP-eF710
p-mTOR-PE
PE-A :: pmTOR : PE - Area
D
FMO p-mTOR
FMO p-S6
NK Cells only
NK Cells co-cultured with TIMP-1 WT Cancer Cells
3 h (no IL-2)
3 h (+ IL-2)
24 h (no IL-2)
24 h (+ IL-2)
p-S6-PerCP-eF710
p-mTOR-PE
E
p < 0.0001
p < 0.0001
p = 0.0136
p = 0.0004
p = 0.0111
p = 0.8474
p < 0.0001
p < 0.0001
p = 0.2866
p = 0.0040
p = 0.0193
p = 0.9832
Fold Change MFI p-STAT3 (Tyr705)
2 min
5 min
10 min
30 min
UNT
100 ng/mL TIMP-1
250 ng/mL TIMP-1
500 ng/mL TIMP-1

Fig. S6 TIMP-1-dependent regulation of signaling pathways in primary NK cells, related to Fig. 6. (A,B) Pathways enriched in highly cytotoxic intratumoral NK cells in our snRNA-seq dataset (A) and scRNA-seq Steele *et al.* dataset (B) (see *Fig. 6A-C*), shown by Odds ratios (left heatmaps) and significance (right heatmaps; Fisher exact test). DEGs between NK1_c1 and indicated other clusters (columns) were calculated using the presto implementation of the Wilcoxon rank sum test and auROC analysis. Significantly (adj. p val < 0.05) enriched genes were investigated for pathway enrichment by Enrichr using the Hallmark reference gene sets. X indicates pathway was not enriched. AKT, protein kinase B; IL-2, interleukin-2; mTOR(C1), mechanistic target of rapamycin (complex 1); PI3K, phosphoinositide 3-kinase; STAT5, signal transducer and activator of transcription 5; UV, ultraviolet. **(C)** Representative flow cytometry plots showing the gating strategy for NK cell signaling assays in co-culture experiments with MIA PaCa-2 cell lines (see *Fig. 6D,F,G*) **(D)** Representative flow cytometry plots showing gating strategy to analyze intracellular p-mTOR (Ser2448) and p-S6 (Ser235/236) levels in NK cells after co-culture with MIA PaCa-2 cell lines for indicated timepoints, in the presence or absence of IL-2 (see *Fig. 6F,G*). **(E)** Quantification of NK cell p-STAT3 (Tyr705) levels upon exposure to different concentrations of recombinant human (rh) WT-TIMP-1 for different time points as indicated (2 and 5 min: UNT, n = 9; all TIMP-1 concentrations, n = 6; 10 and 30 min: all conditions, n = 6). For MFI calculations in (E), background signals of FMO controls were subtracted and fold-changes were calculated by normalizing to the UNT controls. Statistical significance was calculated compared to respective UNT controls by two-way ANOVA and Dunnett test for multiple comparison correction. STAT3, signal transducer and activator of transcription 3; UNT, untreated. Data in (E) showing biological replicates and represented as box and whiskers plots.

Fig. S7

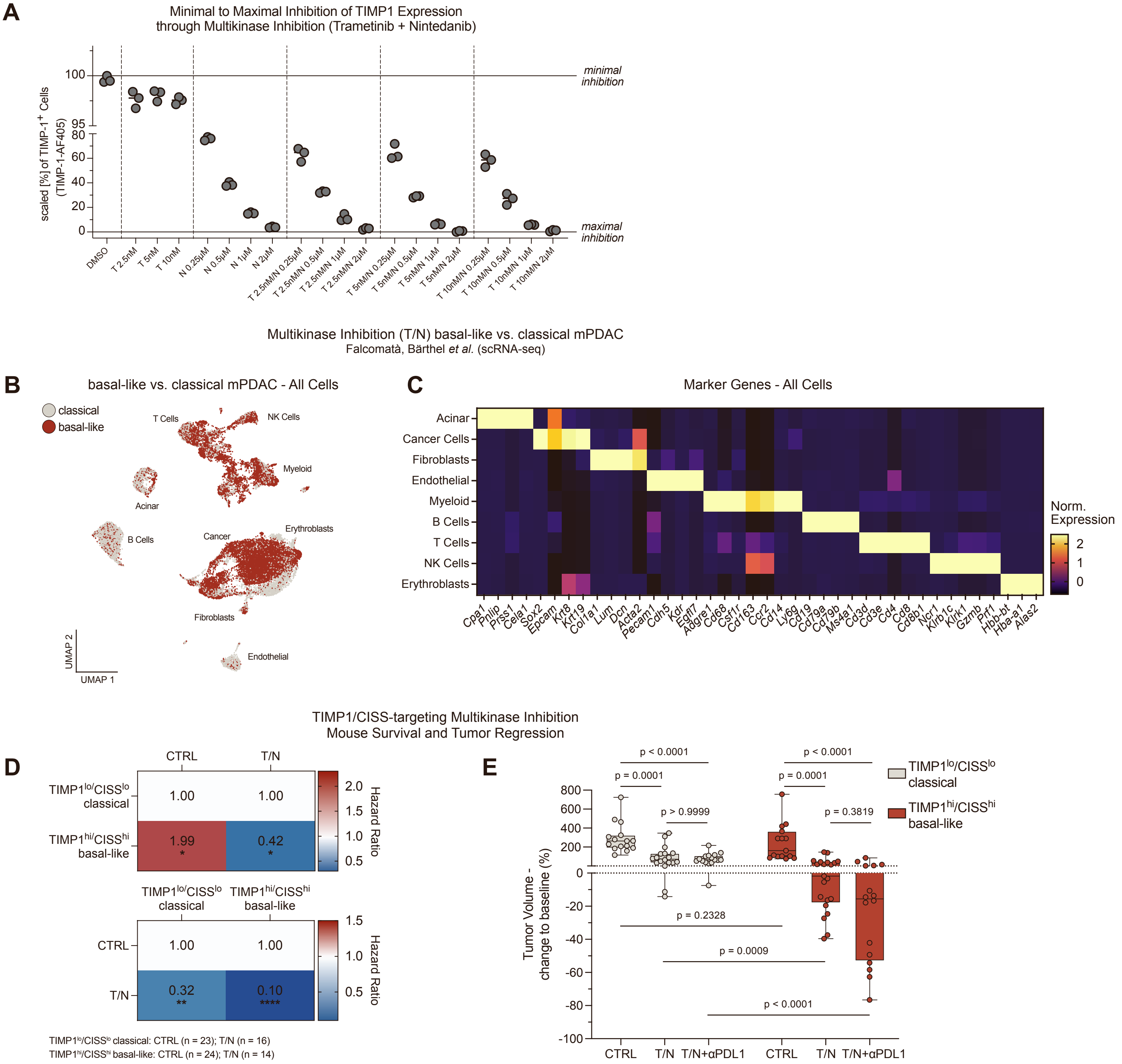
A
Minimal to Maximal Inhibition of TIMP1 Expression through Multikinase Inhibition (Trametinib + Nintedanib)
scaled [%] of TIMP-1+ Cells (TIMP-1-AF405)
minimal inhibition
maximal inhibition
DMSO
T 2.5nM
T 5nM
T 10nM
N 0.25μM
N 0.5μM
N 1μM
N 2μM
T 2.5nM/N 0.25μM
T 2.5nM/N 0.5μM
T 2.5nM/N 1μM
T 2.5nM/N 2μM
T 5nM/N 0.25μM
T 5nM/N 0.5μM
T 5nM/N 1μM
T 5nM/N 2μM
T 10nM/N 0.25μM
T 10nM/N 0.5μM
T 10nM/N 1μM
T 10nM/N 2μM
Multikinase Inhibition (T/N) basal-like vs. classical mPDAC
Falcomatà, Bärthel et al. (scRNA-seq)
B
basal-like vs. classical mPDAC - All Cells
classical
basal-like
T Cells
NK Cells
Myeloid
Acinar
B Cells
Cancer
Erythroblasts
Fibroblasts
Endothelial
UMAP 2
UMAP 1
C
Marker Genes - All Cells
Acinar
Cancer Cells
Fibroblasts
Endothelial
Myeloid
B Cells
T Cells
NK Cells
Erythroblasts
Norm. Expression
Cpa1 Pnlip Prss1 Cela1 Sox2 Epcam Krt8 Krt19 Col1a1 Lum Dcn Acta2 Pecam1 Cdh5 Kdr Egfl7 Adgre1 Cd68 Csf1r Cd163 Ccr2 Cd14 Ly6g Cd19 Cd79a Cd79b Ms4a1 Cd3d Cd3e Cd4 Cd8 Cd8b1 Ncr1 Klrb1c Klrk1 Gzmb Prf1 Hbb-bt Hba-a1 Alas2
TIMP1/CISS-targeting Multikinase Inhibition
Mouse Survival and Tumor Regression
D
CTRL
T/N
TIMP1lo/CISSlo classical
TIMP1hi/CISShi basal-like
1.00
1.00
1.99 *
0.42 *
Hazard Ratio
TIMP1lo/CISSlo classical
TIMP1hi/CISShi basal-like
CTRL
T/N
1.00
1.00
0.32 **
0.10 ****
Hazard Ratio
TIMP1lo/CISSlo classical: CTRL (n = 23); T/N (n = 16)
TIMP1hi/CISShi basal-like: CTRL (n = 24); T/N (n = 14)
E
Tumor Volume - change to baseline (%)
p < 0.0001
p = 0.0001
p > 0.9999
p < 0.0001
p = 0.0001
p = 0.3819
p = 0.2328
p = 0.0009
p < 0.0001
TIMP1lo/CISSlo classical
TIMP1hi/CISShi basal-like
CTRL
T/N
T/N+αPDL1
CTRL
T/N
T/N+αPDL1

Fig. S7 Multikinase Inhibition using Trametinib and Nintedanib in PDAC *in vitro* and *in vivo*, related to Fig. 7. (A) Intracellular TIMP-1 levels in MIA PaCa-2 cells upon treatment with indicated concentrations of MEK inhibitor Trametinib (T) and RTK inhibitor Nintedanib (N), or DMSO control, using flow cytometry (n = 3; biological replicates). [%] of TIMP-1$^+$ cells identified by comparison to respective unstained controls and scaled from minimal to maximal inhibition for identification of drug synergy (see *Fig. 7E*). **(B,C)** UMAP embedding of all cells (B) from mouse pancreas scRNA-seq data ([S10]; see *Fig. 7F,G*), colored by post-hoc cell-type annotations, and selected marker gene expression (C) for annotated cell types. **(D,E)** *In vivo* responses to treatment on mouse survival (D) and tumor regression (E; biological individuals) ([S10]; see *Fig. 7F*). For survival data (D), statistics were performed using logrank Mantel-Cox test of Kaplan-Meier survival curves for indicated comparisons (lower rows vs. upper rows). For tumor regression (E), indicated groups were compared using Kruskal-Wallis tests with Dunn's test (upper) or Mann-Whitney tests (lower).

Supplemental References

S1: Cao, L., Huang, C., Cui Zhou, D., Hu, Y., Lih, T.M., Savage, S.R., Krug, K., Clark, D.J., Schnaubelt, M., Chen, L., et al. (2021). Proteogenomic characterization of pancreatic ductal adenocarcinoma. Cell 184, 5031–5052 e5026. 10.1016/j.cell.2021.08.023.

S2: Abdollahi, A., Schwager, C., Kleeff, J., Esposito, I., Domhan, S., Peschke, P., Hauser, K., Hahnfeldt, P., Hlatky, L., Debus, J., et al. (2007). Transcriptional network governing the angiogenic switch in human pancreatic cancer. Proc Natl Acad Sci U S A 104, 12890–12895. 10.1073/pnas.0705505104.

S3: Moffitt, R.A., Marayati, R., Flate, E.L., Volmar, K.E., Loeza, S.G., Hoadley, K.A., Rashid, N.U., Williams, L.A., Eaton, S.C., Chung, A.H., et al. (2015). Virtual microdissection identifies distinct tumor- and stroma-specific subtypes of pancreatic ductal adenocarcinoma. Nat Genet 47, 1168–1178. 10.1038/ng.3398.

S4: Diaferia, G.R., Balestrieri, C., Prosperini, E., Nicoli, P., Spaggiari, P., Zerbi, A., and Natoli, G. (2016). Dissection of transcriptional and cis-regulatory control of differentiation in human pancreatic cancer. EMBO J 35, 595–617. 10.15252/embj.201592404.

S5: Steele, N.G., Carpenter, E.S., Kemp, S.B., Sirihorachai, V.R., The, S., Delrosario, L., Lazarus, J., Amir, E.D., Gunchick, V., Espinoza, C., et al. (2020). Multimodal Mapping of the Tumor and Peripheral Blood Immune Landscape in Human Pancreatic Cancer. Nat Cancer 1, 1097–1112. 10.1038/s43018-020-00121-4.

S6: Netskar, H., Pfefferle, A., Goodridge, J.P., Sohlberg, E., Dufva, O., Teichmann, S.A., Brownlie, D., Michaelsson, J., Marquardt, N., Clancy, T., et al. (2024). Pan-cancer profiling of tumor-infiltrating natural killer cells through transcriptional reference mapping. Nat Immunol 25, 1445–1459. 10.1038/s41590-024-01884-z.

S7: Crinier, A., Milpied, P., Escaliere, B., Piperoglou, C., Galluso, J., Balsamo, A., Spinelli, L., Cervera-Marzal, I., Ebbo, M., Girard-Madoux, M., et al. (2018). High-Dimensional Single-Cell Analysis Identifies Organ-Specific Signatures and Conserved NK Cell Subsets in Humans and Mice. Immunity 49, 971–986 e975. 10.1016/j.immuni.2018.09.009.

S8: Collins, P.L., Cella, M., Porter, S.I., Li, S., Gurewitz, G.L., Hong, H.S., Johnson, R.P., Oltz, E.M., and Colonna, M. (2019). Gene Regulatory Programs Conferring Phenotypic Identities to Human NK Cells. Cell 176, 348–360 e312. 10.1016/j.cell.2018.11.045.

S9: Smith, S.L., Kennedy, P.R., Stacey, K.B., Worboys, J.D., Yarwood, A., Seo, S., Solloa, E.H., Mistretta, B., Chatterjee, S.S., Gunaratne, P., et al. (2020). Diversity of peripheral blood human NK cells identified by single-cell RNA sequencing. Blood Adv 4, 1388–1406. 10.1182/bloodadvances.2019000699.

S10: Falcomatà, C., Bärthel, S., Widholz, S.A., Schneeweis, C., Montero, J.J., Toska, A., Mir, J., Kaltenbacher, T., Heetmeyer, J., Swietlik, J.J., et al. (2022). Selective multi-kinase inhibition sensitizes mesenchymal pancreatic cancer to immune checkpoint blockade by remodeling the tumor microenvironment. Nat Cancer 3, 318–336. 10.1038/s43018-021-00326-1.
